# 4,5‐Diazafluorene‐Based Donor–Acceptor Small Molecules as Charge Trapping Elements for Tunable Nonvolatile Organic Transistor Memory

**DOI:** 10.1002/advs.201800747

**Published:** 2018-09-06

**Authors:** Yang Yu, Lin‐Yi Bian, Jian‐Guo Chen, Qi‐Hao Ma, Yin‐Xiang Li, Hai‐Feng Ling, Quan‐You Feng, Ling‐Hai Xie, Ming‐Dong Yi, Wei Huang

**Affiliations:** ^1^ Centre for Molecular Systems and Organic Devices (CMSOD) Key Laboratory for Organic Electronics and Information Displays and Institute of Advanced Materials (IAM) Jiangsu National Synergetic Innovation Center for Advanced Materials (SICAM) Nanjing University of Posts & Telecommunications 9 Wenyuan Road Nanjing 210023 P. R. China; ^2^ Shaanxi Institute of Flexible Electronics (SIFE) Northwestern Polytechnical University (NPU) 127 West Youyi Road Xi'an 710072 P. R. China

**Keywords:** diazafluorene, hole blocking effects, restrain charge leakage, small molecular elements, weak intermolecular interactions

## Abstract

Three diazafluorene derivatives triphenylamine (TPA)(PDAF)*_n_* (*n* = 1, 2, 3) serving as small molecular elements are designed and synthesized via concentrated sulfuric acid mediated Friedel–Crafts reaction. With highly nonplanar topological configuration, TPA(PDAF)_3_ shows weaker intermolecular interaction in the solid states and thus exhibits single nanomolecular behavior, which is crucial for charge stored and retained in an organic field‐effect transistor (OFET) memory device. Furthermore, diazafluorene derivatives possess a completely separate highest occupied molecular orbital/lowest unoccupied molecular orbital, which offers ideal hole and electron trapping sites. As charge storage elements, triphenylamine groups provide the hole trapping sites, while diazafluorene units provide the electron trapping sites and act as a hole blocking group to restrain the leakage of stored holes trapped in triphenylamine. The pentacene‐based OFET memory device with solution‐processing TPA(PDAF)_3_ shows a good hole‐trapping ability, high hole trapping density (4.55 × 10^12^ cm^−2^), fast trapping speed (<20 ms), a large memory window (89 V), and a tunable ambipolar memory behavior. The optimized device shows a large ON/OFF current ratio (2.85 × 10^7^), good charge retention (>10^4^ s), and reliable endurance properties. This study suggests that diazafluorene based donor–acceptor small molecular elements have great promise for high‐performance OFET memory.

## 1 Introduction

Nonvolatile organic field‐effect transistor (OFET) memories are gaining considerable interest for their potential applications as primary component in organic electronics because of nondestructive reading, single‐transistor realization, and good compatibility with the complementary metal‐oxide semiconductor circuits.^1^ Different from the typical OFET structures, OFET memory device dominated by charge trapping mechanism requires an additional charge storage layer, and up to date, two main trapping mediums can be distinguished: nanofloating gate^2^ and polymer electrets.^3^ In nanofloating gate memory devices, charge trapping sites such as metal nanoparticles or nanocrystals are dispersed and isolated inside a matrix and the memory performances are influenced by specific sizes and distribution which require high processing technic. The drawbacks of solution‐processed polymer electret memories are also obvious such as uncontrollable molecular weight, supramolecular interaction between polymer chains, and ambiguous charge trapping mechanism.^4^ Thus, it is still a challenge to develop new charge trapping elements to improve the charge density and charge endurance,^5^ compared with industrialized electronic products. The small molecular elements show obviously superiority in high‐density and high‐speed charge storage^6^ with well‐defined molecular structure, flexible synthesis, easy purification, and tunable bandgap.[[qv: 6a,b,7]]

Although the investigation in the relationship between charge trapping behavior and electronic structure is rarely reported in small molecular elements based memory, there are still some influence factors for reference especially from the polymer electrets. Among the reported polymer electrets, polystyrene (PS)‐based side‐chain polymers[[qv: 3a,8]] and donor–accepter polyimides^9^ were widely investigated as template polymers to explore the relationship between electronic structure and memory performance. For developing efficient polymer electrets, donor–accepter electronic structure,[[qv: 9a‐c]] limited effective conjugated length,[[qv: 8d,10]] hydrophilicity,[[qv: 8a]] dielectric polarity,^11^ suitable highest occupied molecular orbital (HOMO)/lowest unoccupied molecular orbital (LUMO) energy levels,[[qv: 8d]] and film quality of the polymer[[qv: 3b]] should be considered. Some designing strategies have also been proved to be effective for charge injection, storage, and transporting in OFET memory. For example, star‐shaped polymers with aromatic group for charge trapping centers and alkyl chains to restrain the leakage of stored charges could provide more charge trapping sites and facilitate charge concentration and distribution.[[qv: 8c,12]] By introducing branched π‐conjugation interrupted structures^13^ or large steric hindrance building blocks,^14^ the organic elements based devices also exhibit broad memory windows and long retention time. Through covalently linking between charge trapping cores and alkyl chains, Chen and co‐workers and Tao and co‐workers reported two new organic nanofloating gate elements and the memory devices showed long retention ability and good endurance.[[qv: 15a,b]] The new organic nanofloating gate structures through covalent linking showed obvious advantage compared with the conventional nanofloating gate memories, which generally use nanoparticles dispersed and isolated in a polymeric insulator. Considering the organic elements design experience, both charge trapping sites and unconjugated structures should be considered to realize charge concentration and distribution controllable. However, for small molecular elements, it is difficult to combine the above factors, especially the effective unconjugated structures that surround the charge trapping sites for preserving stored charges. Recently, our group reported a wide‐bandgap molecule as charge trapping layer and the memory device showed impressive memory performance.^14^ Different from narrow‐bandgap small molecules usually using as charge trapping sites in nanofloating gate OFET memories,[[qv: 2a,6c,16]] wide‐bandgap molecules possess high potential barrier, which are suitable to retain trapped charges and emerge great promise for charge trapping elements.

In order to achieve efficient small molecular elements, in this study, donor–acceptor type molecules with triphenylamine (TPA) and 4,5‐diazafluorene units were designed and synthesized to serve as charge storage layers in OFET memory devices. As a classic hole‐transporting moiety, TPA unit has been proved to be an efficient hole trapping core in OFET memory.^17^ 4,5‐diazafluorene derivatives, as electron‐transporting or hole‐blocking materials, have been widely used in organic light‐emitting diodes,^18^ while they are rarely reported as charge trapping elements in OFET memory devices. Here, triphenylamine groups provide the hole trapping sites, while the steric hindrance 4,5‐diazafluorene units surrounding by the triphenylamine act as the electron trapping sites. Additionally, because of the hole‐blocking effect, 4,5‐diazafluorene units could restrain the leakage of stored hole trapped in TPA. Due to the strong electron affinity, 4,5‐diazafluorene based small molecules in this work possess lower LUMO, which is beneficial for electron injection and completely separated HOMO and LUMO, which offers ideal hole and electron trapping sites.^19^ Furthermore, with highly nonplanar topological configuration, the elements showed weaker intermolecular interaction in solid states and thus exhibited single nanomolecular behavior with random molecular distribution,^20^ which could be viewed as isolated molecular trapping centers for charge stored and restained. Similar with the organic nanofloating gate elements, triphenylamine groups act as charge trapping cores while 4,5‐diazafluorene units perform the role of “unconjugated structures” for hole trapped. For comparison, different amounts of 4,5‐diazafluorene units connecting with TPA, TPA(PDAF)_1_, TPA(PDAF)_2_, and TPA(PDAF)_3_ were prepared to explore the role of the electronic structure and charge trapping mechanism. Herein, the preparations, characterizations, spectra, electrical properties, and theory calculation of TPA(PDAF)*_n_* were systematically explored. It is worth mentioning that the TPA(PDAF)*_n_* elements were prepared by simple solution‐processing methods instead of vacuum evaporation like most small molecules reported.[[qv: 6b,7b,21]] The charge‐trapping and ambipolar memory characteristics of pentacene‐based OFET memory using TPA(PDAF)*_n_* as charge storage layers were studied. In addition, the relationships between electronic structure of TPA(PDAF)*_n_*, charge trapping, and memory characteristics was discussed in detail.

## 2 Results and Discussion

### 2.1 Synthesis and Characterization

**Scheme 1:**
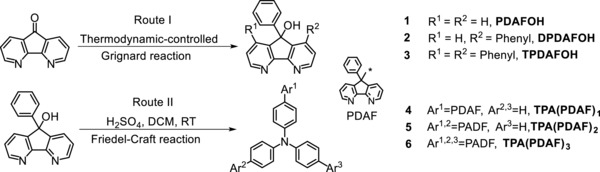
Synthetic procedures for the nPDAFOH (compounds 1, 2, 3) and TPA(PDAF)*_n_* (compounds 4, 5, 6), *n* = 1, 2, 3. Reaction conditions: (route I) phenyl magnesium bromide, tetrahydrofuran, (1) −78 °C, 15 min; (2) −10 °C, 10 min; (3) 40 °C, 10 min. (route II) triphenylamine, concentrated H_2_SO_4_, dichloromethane, room temperature.

The synthesis routes of tertiary alcohols and TPA(PDAF)*_n_* (*n* = 1, 2, 3) are listed in **Scheme** **1** (route I and route II). 4,5‐diazafluorene‐9‐one (DAFO) was prepared as precious works in our group.^22^ Owing to the strong electron‐deficiency of two pyridine rings, 1,8‐position of DAFO is activated enough to occur michael addition reaction under the attack of grignard reagent. Tertiary alcohol **1** was obtained at −78 °C, while by‐products tertiary alcohols **2** and **3** were synthesized at higher temperature. Therefore, route I was thermodynamic‐controlled grignard reaction. Subsequently, TPA(PDAF)*_n_* (*n* = 1, 2, 3) was smoothly prepared by **1** and TPA via concentrated sulfuric acid‐mediated Friedel–Crafts reaction with the yields of 56%, 46%, and 75%, respectively. Chemical structures of the products were confirmed by ^1^H and ^13^C NMR, high‐resolution mass spectrometry (HRMS), and fortunately, the single crystals of compounds **3** and **6** were also obtained. The data could be seen in the Supporting Information. The single crystals of compounds **3** and **6** were unambiguously characterized by X‐ray crystallographic analysis, as shown in Figure S1 (Supporting Information). The single crystal of TPA(PDAF)_3_ revealed a 3D topology shape, consisting a core unit of TPA and three open‐arms of 4,5‐diazafluorene moieties. Owing to the large steric hindrance of diazafluorene moieties connecting to TPA, TPA(PDAF)_3_ displayed the asymmetric conformation in three directions with open‐armed at angles of 122°, 113°, and 125°. Compared with the angle values of the single crystals of TPA (Figure S1, Supporting Information), TPA(PDAF)_3_ displayed a larger distortion derived from the steric hindrance. All compounds dissolved well in common solvents such as chloroform, tetrahydrofuran, and dichloromethane. The thermal properties of TPA(PDAF)*_n_* (*n* = 1, 2, 3) were estimated using thermogravimetric analysis (TGA) and differentialscanning calorimetry (DSC). The DSC curves indicate that three diazafluorene derivatives show no glass phase transition and melting point by heating to 195 °C (Figure S2, Supporting Information). The degradation temperatures (*T*_d_, temperature corresponding to 5% weight loss) of diazafluorene derivatives are 354, 440, and 485 °C, respectively (Figure S3, Supporting Information). With increased diazafluorene units, the TPA(PDAF)*_n_* show enhanced thermal stability probably to the rigid and steric hindrance structure that suppresses the phase transition process.^23^ These high *T*_d_ values suggested the good thermal stability of TPA(PDAF)*_n_* for the application as charge storage layers in OFET memory devices.

### 2.2 Optical and Electrochemical Properties

**Figure 1:**
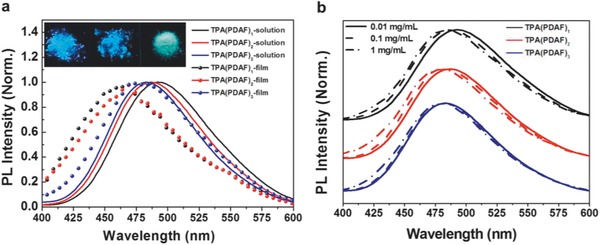
a) The PL spectra of TPA(PDAF)*_n_* (*n* = 1, 2, 3) in chloroform solution (10^−5^ mol L^−1^) and in film state. Photos of TPA(PDAF)*_n_* powder under 365 nm UV light (inset). b) PL spectra of TPA(PDAF)*_n_* in chloroform solution at various concentrations: 0.01 mg mL^−1^ (solid), 0.1 mg mL^−1^ (dashed), and 1 mg mL^−1^ (dashed dotted).

The optical properties of the compounds were investigated by UV–visible (UV–vis) and photoluminescence (PL) analysis in solution and solid films. The absorption and emission spectra of TPA(PDAF)*_n_* (*n* = 1, 2, 3) in solutions and films are plotted in Figures S4 and S5 (Supporting Information), with the corresponding data summarized in **Table** **1**. The three diazafluorene derivatives exhibited a similar absorption around 300–330 nm, which was originated from the π–π* transition of the TPA and diazafluorene units.[[qv: 18b]] In chloroform solution, TPA(PDAF)_1_, TPA(PDAF)_2_, and TPA(PDAF)_3_ showed absorption peaks at 308, 311, and 312 nm, respectively (Table 1). Compared with the solution absorption, hypsochromic shifts of 10, 3, and 1 nm were observed in solid state shown in Figure S5 (Supporting Information), which probably due to weaker intermolecular interaction with increased diazafluorene units. TPA(PDAF)*_n_* (*n* = 1, 2, 3) in solutions exhibits emission spectra with peaks at 494, 487, and 483 nm, respectively. Similar with absorption phenomenon, hypsochromic shifts of 28, 18, and 1 nm were observed in film, as **Figure** **1**a showed. Inset in Figure 1a shows the image of TPA(PDAF)*_n_* powder under 365 nm UV light. Photoluminescence spectra in several different polar solvents were also measured and shown in Figure S6 (Supporting Information). Three diazafluorene derivatives exhibited obviously solvent‐dependent fluorescence that is the higher polarity of the solvent, the more pronounced red‐shift of the emission maxima, indicating the complicated excited states and energy transfer occur.^22^ With increased concentration in solutions, hypsochromic shifts of emission peaks were also found, and TPA(PDAF)_3_ showed the smallest change (Figure 1b). Compared with the absorption and emission spectra of the solution and the film of diazafluorene derivatives, TPA(PDAF)_3_ presented the smallest spectra peaks changed and stokes shift, suggesting weakest aggregation and packing in the films.^13^ And it also indicated that the full end‐capping with large steric hindrance in TPA(PDAF)_3_ would suppress the molecular aggregation to a certain degree. In solid state, weaker intermolecular interaction of TPA(PDAF)_3_ revealed single molecular behavior creating random molecular distribution, which could be considered as isolated molecular trapping centers, and minor energy transfer occurred between the centers. As a result, it could be beneficial for charge stored and restrain the charge leakage of charges as charge storage layers in OFET memory devices.

**Table 1:** Photophysical, thermal, and electrochemical properties of TPA(PDAF)*_n_* (*n* = 1, 2, 3)

Molecule	*T*_d_ [°C]	λ_abs, max/nm_		λ_PL,max/nm_		*E*_g_^opt^ [eV]	*E*_g_ [eV]	*E*_HOMO_ [eV]	*E*_LUMO_ [eV]	EA [eV]
		Solution	Film	Solution	Film					
TPA(PDAF)_1_	354	308	298	494	466	3.14	2.95	−5.40	−2.45	−0.11
TPA(PDAF)_2_	440	311	308	487	469	3.11	2.80	−5.39	−2.59	−0.52
TPA(PDAF)_3_	485	312	311	483	482	3.08	2.69	−5.38	−2.69	−0.68

The cyclic voltammetry (CV) curves of TPA(PDAF)*_n_* films are showed in Figure S7 (Supporting Information), with the electrochemical properties of TPA(PDAF)*_n_* (*n* = 1, 2, 3) listed in Table 1. The HOMO energy levels of TPA(PDAF)_1_, TPA(PDAF)_2_, and TPA(PDAF)_3_ were −5.40, −5.39, and −5.38 eV, respectively.And the LUMO energy levels were −2.45, −2.59, and −2.69 eV, respectively. As a result, the electrochemical bandgaps were 2.95, 2.80, 2.69 eV, respectively. The energy levels revealed that the introduction of 4,5‐diazafluorene groups could lead to significant reduction in LUMO levels, while less change occurred in HOMO level (only 0.01 eV). Such low LUMO levels could reduce the energy barrier which is defined as the difference value of the LUMO/HOMO levels between semiconductor and charge trapping elements, and facilitate charge injection and avoid the charge escaping from the trapping layer. Besides, in the anodic successive multiple potential scans, as Figure S8 (Supporting Information) shown, TPA(PDAF)_3_ exhibited most stable reversible oxidation processes. The electrochemical results demonstrated that TPA(PDAF)_3_ end‐capping with large bulky steric hindrance imparts extraordinarily high electrochemical stability, which is crucial for the stability of electrical devices.

### 2.3 Theory Calculation

**Figure 2:**
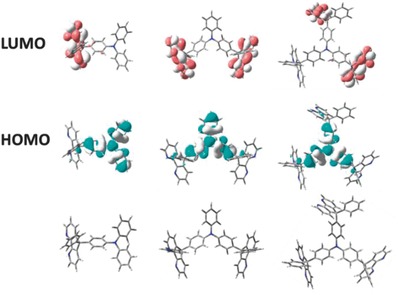
The calculated HOMO and LUMO distribution for TPA(PDAF)_1_, TPA(PDAF)_2_, and TPA(PDAF)_3_. The optimized conformation with energy minimization structure of TPA(PDAF)*_n_* (*n* = 1, 2, 3).

To further demonstrate the intramolecular charge transport and the influence of carrier transporting groups on the frontier molecular orbitals, all the molecules are evaluated by density functional theory (DFT) calculations. The geometry optimization and frontier molecular orbitals are shown in **Figure** **2**. Obviously, their HOMO orbitals are completely localized on the electron‐rich triphenylamine moiety and LUMO orbitals are entirely localized on electron‐deficient 4,5‐diazafluorene moiety. Considering TPA and diazafluorene are interconnected via sp^3^‐hybridized C atoms, the complete separation of HOMO/LUMO confirms that there are minimal intramolecular interactions between the two moieties. The complete separation of frontier molecular orbitals of diazafluorene derivatives can facilitate the balance of carrier injecting and transporting, and offer ideal hole and electron trapped sites.^24^ The electron affinities (EA) of TPA(PDAF)_1_, TPA(PDAF)_2_, TPA(PDAF)_3_ were also calculated with the values of −0.11, −0.52, −0.68 eV, respectively (Table 1). LUMO energy level generally decreased with increased EA,^25^ thus TPA(PDAF)_3_ possessed lowest LUMO, which is beneficial for electron injection as charge trapping element. The 3D diameter of TPA(PDAF)_3_ is about 1.8 nm × 1.6 nm × 0.9 nm based on the single crystal structure (Figure S9, Supporting Information). The multidimensional conformation suggests that the end‐capped bulky molecule TPA(PDAF)_3_ owned highly nonplanar topological configuration, which could suppress π–π stacking and aggregation in the solid states (as shown in optical properties). In this regard, TPA(PDAF)_3_ may exhibit single nanomolecular behavior with weaker intermolecular interaction, which is critical for charge storage and retention as charge storage layers in OFET memory devices.

### 2.4 Characteristics of OFET Memory Devices with TPA(PDAF)*_n_* (*n* = 1, 2, 3)

**Figure 3:**
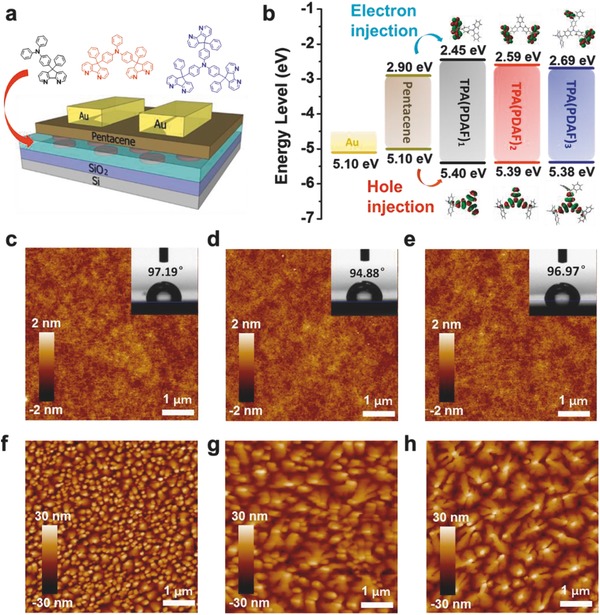
a) Schematic structure of OFET memory device using TPA(PDAF)*_n_*. b) Energy level diagram of Au electrode, pentacene semiconductor, and TPA(PDAF)*_n_*, and charges trapping in programming and erasing processes. AFM topographic images of c) TPA(PDAF)_1_, d) TPA(PDAF)_2_, and e) TPA(PDAF)_3_ films on SiO_2_/Si substrates. The inset figures are the contact angles of the TPA(PDAF)*_n_* film surfaces (side view of a drop of water). AFM topographic images of pentacene on different surfaces: f) TPA(PDAF)_1_, g) TPA(PDAF)_2_, h) TPA(PDAF)_3_ modified SiO_2_ surface.

**Figure** **3**a shows the schematic diagram of pentacene based OFET memory devices, in which TPA(PDAF)*_n_* (*n* = 1, 2, 3) were used as the charge storage elements. For OFETs, the morphology and surface energy of charge trapping elements are critical factors to influence the growth and crystallinity of overlaying semiconductor. The TPA(PDAF)*_n_* (*n* = 1, 2, 3) based charge trapping films were fabricated by solution spin‐coating process on bare SiO_2_ substrates. The atomic force microscopy (AFM) images of the TPA(PDAF)*_n_* films (Figure 3c–e) exhibited smooth surface morphology with root‐mean‐square roughness as 0.238, 0.229, and 0.218 nm, respectively, which were favorable for the formation of high mobility organic semiconductor films. The representative contact angles of the TPA(PDAF)*_n_* films are shown in inset picture of Figure 3c–e. The hydrophobic characteristics of TPA(PDAF)*_n_* (*n* = 1, 2, 3) are with water contact angles of 97.19°, 94.88°, and 96.97°, respectively. The hydrophobic surfaces ensure good wetting and well‐formed growth of pentacene on the smooth electret surface.[[qv: 8a]] AFM images of pentacene on top of TPA(PDAF)*_n_* films are shown in Figure 3f–h. The pentacene deposited on TPA(PDAF)_1_ exhibited a grain domain with the size about 0.2–0.3 µm, while pentacene on TPA(PDAF)_2_ and TPA(PDAF)_3_ exhibited a bigger grain domain with the sizes of ≈0.4–0.6 µm and 0.6–1 µm, respectively. It may be ascribed to the weaker intermolecular interaction of TPA(PDAF)_3_ in solid state that provided a nanoscaled nucleation center for crystallinity of pentacene.

The output and transfer characteristics of the devices based on TPA(PDAF)*_n_* (*n* = 1, 2, 3) are shown in Figure S10 (Supporting Information), and the calculated transistor parameters are listed in **Table** **2**. These curves exhibited typical p‐type behavior with the hole mobility of 0.018, 0.048, and 0.053 cm^2^ V^−1^ s^−1^, respectively. The increase of mobility was attributed to the increased crystalline and grain size of pentacene. The transfer curves of these devices showed the initial threshold voltages (*V*_th_) of −4.8, −18.7, −11.2, and ON/OFF current ratios of 5.1 × 10^4^, 11.1 × 10^5^, and 3.96 × 10^4^, respectively, which were enough to distinguish between “0” and “1” digital states clearly.

**Table 2:** Transistor and memory performances of pentacene‐based OFET memories with TPA(PDAF)*_n_* (*n* = 1, 2, 3)

Charge trapping elements	*µ* [cm^2^ V^−1^ s^−1^]	*V*_th_ [V]	*I*_ON_/*I*_OFF_	Negative window [V]	Positive window [V]
TPA(PDAF)_1_	0.018 ± 0.01	−4.8 ± 0.5	5.1 × 10^4^	50.9a)	1.5b)
TPA(PDAF)_2_	0.048 ± 0.01	−18.7 ± 1.1	1.11 × 10^5^	54.3a)	12.1b)
TPA(PDAF)_3_	0.053 ± 0.02	−11.2 ± 0.7	3.96 × 10^4^	63.2a)	26.2b)
10%TPA(PDAF)_1_@PS	0.20 ± 0.2	−14.6 ± 0.8	8.5 × 10^6^	24.75c)	1.15d)
10%TPA(PDAF)_2_@PS	0.25 ± 0.15	−12.6 ± 1.5	2.3 × 10^7^	33.98c)	17.4d)
10%TPA(PDAF)_3_@PS	0.55 ± 0.1	−11.04 ± 1.2	2.85 × 10^7^	38.30c)	27.0d)

a **Programming conditions**: ^a)^ *V*_G_ = −100 V for 20 ms

^b)^ *V*_G_ = 100 V assistance of light for 1 s

^c)^ *V*_G_ = −80 V for 20 ms

^d)^ *V*_G_ = 80 V assistance of light for 1 s.

### 2.5 Memory Characteristics

**Figure 4:**
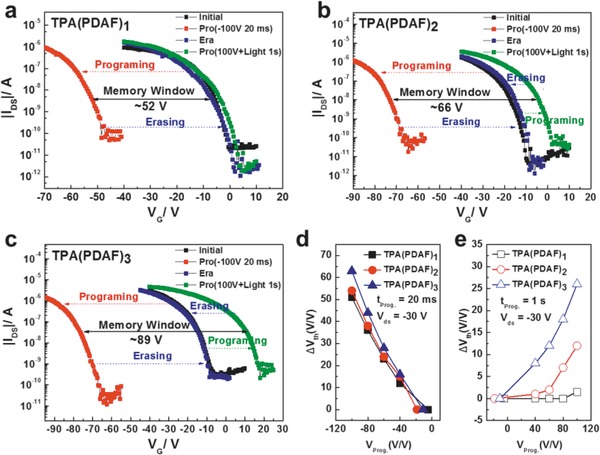
Electrical transfer curves of the a) TPA(PDAF)_1_, b) TPA(PDAF)_2_, and c) TPA(PDAF)_3_ based devices for the programming and erasing processes in both negative and positive gate voltages. Shifts of threshold voltage (Δ*V*_th_) and linear fitting curves under different programming gate bias (*V*_Prog_) with the d) negative and e) positive bias.

To investigate the influence from 4,5‐diazafluorene group on transistor memory devices, we applied appropriate gate pulse to lead the shifts of transfer curves, which brought about high conductance (ON) and low conductance (OFF) states. Memory window (Δ*V*_th_) is defined as the difference between the *V*_th_ of the programming (PGM) and erasing (ERS) states. It should be noted that the thickness of blocking dielectric layer SiO_2_ is 300 nm, programming voltage would be relatively large. **Figure** **4**a–c shows the negative and positive shifts of the transfer curves (at drain–source voltage *V*_DS_ = −30 V) of the memory devices. After applying a negative gate bias (*V*_G_ = −100 V for 20 ms) under dark conditions, the transfer curves based TPA(PDAF)_1_, TPA(PDAF)_2_, and TPA(PDAF)_3_ were significantly shifted in the negative direction with threshold voltages (*V*_th_) of −55.7, −73.0, and −74.4 V, respectively, serving as the “programming” process. Subsequently, the memory devices were applied reverse gate bias of 50, 50, and 55 V for TPA(PDAF)_1_, TPA(PDAF)_2_, and TPA(PDAF)_3_ based devices with assistance of light illumination for 1 s, respectively. Then the transfer curves were completely shifted to the positive direction and recovered to their initialstate, serving as the “erasing” process. The negative memory windows between PGM and ERS states were calculated to be 50.9, 54.3, and 63.2 V for TPA(PDAF)_1_, TPA(PDAF)_2_, and TPA(PDAF)_3_ based devices, respectively. In the electric‐programming process, when the programming time was changing from 20 ms to 5 s for TPA(PDAF)*_n_* (*n* = 1, 2, 3) based device, there was no change with the value of Δ*V*_th_ (Figure S11 of the Supporting Information and Figure 6a), which indicated that TPA(PDAF)*_n_* (*n* = 1, 2, 3) provided strong trapping sites, thus displayed a fast trapping speed. Owing to the programming speed limit of our instrument is 20 ms, faster saturation programming speed could be achieved if experimental conditions permitted. The electric‐programming time of TPA(PDAF)*_n_* (<20 ms) was suitable compared with other reported charge trapping elements, such as poly(α‐methylstyrene) (PαMS) (<1 µs),[[qv: 3a]] [2‐(9‐(4‐(octyloxy)phenyl)‐9H‐ﬂuoren‐2‐yl)thiophene]_3_ (WG_3_)(≈1 s),^14^ and diacetylenic‐naphthalenetetracarboxyldiimide‐carrying phosphonic acids (DAND‐PA) (18 s).[[qv: 15b]] The TPA(PDAF)*_n_* (*n* = 1, 2, 3) owned the same hole trapping core TPA unit, and similar potential barrier (approximate HOMO levels) for hole carriers transferred, the widest memory windows of TPA(PDAF)_3_ may be ascribed to the larger torsion angle of triphenylamine unit. As Figure S1 (Supporting Information) showed, the torsion angles of triphenylamine were 111°, 112°, 109° for TPA(PDAF)_1_, 108°, 109°, 113° for TPA(PDAF)_2_, and 122°, 113°, 125° for TPA(PDAF)_3_. The largest torsion angle of TPA(PDAF)_3_ may create higher barrier for trapping more charges deeply and prevent the recombination of segregated charges in the trapping layer.^10^, ^21^ Furthermore, more 4,5‐diazafluorene groups linked to TPA could prevent the stored charges leakage. When applying negative gate bias ranging from −40, −60, −80 to −100 V, the memory devices showed multibit memory windows of 12.0, 23.1, 36.4, and 50.9 V for TPA(PDAF)_1_, 15.2, 24.5, 38.3, and 54.3 V for TPA(PDAF)_2_ and 16.1, 28.3, 44.5, and 63.2 V for TPA(PDAF)_3_, respectively (Figure S12, Supporting Information). Namely, the devices exhibited stepwise shifts of transfer curves with the increase of the negative gate bias, which indicated that these memory devices could process the multibit storage function to achieve high storage density in one cell.

However, when applying positive gate bias ranging from 40, 60, 80 to 100 V, the transfer curves of TPA(PDAF)_1_ based device barely shifted in the positive direction. Whereas the TPA(PDAF)_2_ based device showed slight positive memory windows of 1.0, 2.1, 7.3, and 12.1 V, respectively. Significantly, much bigger memory windows of 8.2, 12.5, 18.3, and 26.2 V were obtained from TPA(PDAF)_3_, respectively (Figure S12, Supporting Information). The shifts of *V*_th_ enhanced with the increased proportion of 4,5‐diazafluorene group. TPA(PDAF)_3_ based device exposited largest positive memory window, because TPA(PDAF)_3_ has the lowest LUMO energy level due to the strongest electron affinity, thus facilitating more electrons transfer from pentacene to trapping layer. Moreover, TPA(PDAF)_3_ possesses the maximum electron‐withdrawing 4,5‐diazafluorene group, which helps for more trapped electrons. As a result, considering the ambipolar memory characteristic, under the conditions of −100 V at hole‐trapping mode and 100 V at electron‐trapping mode, about 52, 66, and 89 V total memory windows were obtained from TPA(PDAF)_1_, TPA(PDAF)_2_, and TPA(PDAF)_3_, respectively, shown in Figure 4a–c, which were better than most reported single‐component small molecular elements based memory devices to the best of our knowledge.[[qv: 6,7b,21]] For verifying the effectiveness of the design strategy of the TPA(PDAF)*_n_*, single‐component charge trapping elements diazafluorene and triphenylamine were also measured and the result electrical characteristics are shown in Figures S13 and S14 (Supporting Information). Although the memory windows can be found in these devices, they were much smaller than the devices with TPA(PDAF)*_n_*, which was clearly indicated that the design strategy of small molecular elements was crucial to achieve the memory performance.

**Figure 5:**
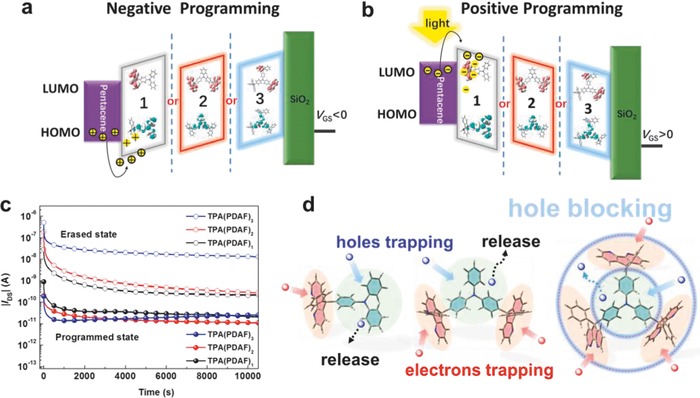
Energy band diagrams of pentacene and TPA(PDAF)*_n_* under a) negative and b) positive gate voltages. c) Retention characteristics of TPA(PDAF)*_n_* based transistor memories. d) The Schematic of molecular charge trapping mechanism.

The possible mechanism was proposed in **Figure** **5**a,b and Figure S15 (Supporting Information). The HOMO/LUMO energy levels of pentacene and TPA(PDAF)*_n_* (*n* = 1, 2, 3) illustrated in Figure 3b. The relatively low HOMO energy levels are suitable for the hole injected from pentacene into TPA(PDAF)*_n_* (*n* = 1, 2, 3). According to Fowler–Nordheim (FN) tunneling mechanism, when applying a negative gate electric field (programming process), the holes were induced through pentacene and transferred into trapping layers, which injected into the HOMO of the molecules and trapped by the triphenylamine moiety, as shown in Figure 5a, leading to a negative shift of *V*_th_. As is well‐known that photosensitive pentacene can generate excitons by light emission. For OFETs, the photogenerated excitons can be separated by applying appropriate gate voltage to generate holes and electrons. For the erasing process, when the reverse gate bias and light irradiation were applied, the trapped holes were neutralized by the injected photoinduced electrons, resulting in the transfer curve returning to a high conductance state.^26^ Corresponding to positive programming, under positive gate bias, electrons overcame the LUMO energy barrier between pentacene and charge trapping molecules, then injected from the LUMO of pentacene into the LUMO of the molecules and trapped by the 4,5‐diazafluorene moiety, shown in Figure 5b. For more studying the charge trapping and intrinsic mechanism, positive ionization potential (IP) and negative EAs were calculated by DFT. The energy of neutral (*E*_0_), cationic (*E*_+_), and anionic (*E*_−_) states of TPA(PDAF)*_n_* molecules are summarized in Table S1 (Supporting Information). When the charge trapping molecule combines a positive charge or a negative charge, the cationic state or anionic state of the molecule were obtained. The values of IP and EA were calculated as IP = *E*_0_(*Q*_0_) – *E*_+_(*Q*_+_) and EA = *E*_0_(*Q*_0_) – *E*_+_(*Q*_+_).[[qv: 25a]] Comparing the values of TPA(PDAF)*_n_*, TPA(PDAF)_3_ has a much lower energy in both charged states (IP = 6.18 eV, EA = −0.68 eV), which facilitate the charge injection and the stabilization of trapped charges.[[qv: 25b]] Therefore, TPA(PDAF)_3_ based device exhibited higher positive and negative memory windows and better charge retention property.

To further analyze the trapping behavior, we plotted the shifts of threshold voltage (Δ*V*_th_) with different programming gate voltage (*V*_Prog_). All the devices displayed nearly linear fittings with Δ*V*_th_ as a function of *V*_Prog_, corresponding to the model of FN tunneling.[[qv: 8b]] As shown in Figure 4d, the negative memory windows of TPA(PDAF)*_n_* increased linearly with the enhanced *V*_Prog_ dramatically which suggested TPA(PDAF)*_n_* possess strong hole storage capability. For the positive memory windows, the Δ*V*_th_ of TPA(PDAF)_2_ and TPA(PDAF)_3_ increased linearly with the increasing *V*_Prog_ (Figure 4e). The larger slope of TPA(PDAF)_3_ indicated a stronger electron storage capability than that of TPA(PDAF)_2_. Larger memory windows could be obtained for TPA(PDAF)_2_ and TPA(PDAF)_3_ if a higher *V*_Prog_ was applied. However, there is no increase for TPA(PDAF)_1_ until applying a *V*_Prog_ beyond 100 V. For TPA(PDAF)*_n_* trapping elements, the magnitude of positive memory windows could be controlled by the proportion of 4,5‐diazafluorene group.

We can calculate the charge trapping density (Δ*n*) according to the equation: Δ*n* = Δ*V*_th_·*C*_i_/*e*, where *e* is the element charge (1.602 × 10^−19^ C), *C*_i_ is the total capacitance per unit area (roughly calculated from the equation: *C*_i_ = 1/*C*_M_ + 1/*C*_SiO2_). Under a programming voltage of *V*_GS_ = −100 V for 20 ms, the calculated Δ*n* in these memory devices were about 3.69 × 10^12^ cm^−2^ for TPA(PDAF)_1_, 3.91 × 10^12^ cm^−2^ for TPA(PDAF)_2_, and 4.55 × 10^12^ cm^−2^ for TPA(PDAF)_3_, respectively. It suggests that the TPA(PDAF)*_n_* based devices have similar hole trapping capacity. The charge trapping density with a magnitude of 10^12^ in OFET memory devices is better than the nanofloating gate memory devices newly reported.^16^, ^27^ Upon the programming voltage of *V*_GS_ = 100 V with the assist of light for 1 s, the calculated Δ*n* was 7.49 × 10^10^ cm^−2^ for TPA(PDAF)_1_, 5.99 × 10^11^ cm^−2^ for TPA(PDAF)_2_, and 1.29 × 10^12^ cm^−2^ for TPA(PDAF)_3_. The experimental results indicated that the strong electronic deficiency of 4,5‐diazafluorene group could generate more electron trapping sites and high charge trapping density to improve memory performance, while the hole trapping ability depends on the core of the TPA. The memory characteristics of TPA(PDAF)*_n_* (*n* = 1, 2, 3) based devices are listed in Table 2. Considering the TPA(PDAF)*_n_* based OFET memory devices have large hole storage windows, tunable electron storage windows, and high charge storage capacity, our approach is a promising strategy for realizing multilevel characteristics memory.

The multiple switching stability of the devices using TPA(PDAF)*_n_* (*n* = 1, 2, 3) as charge trapping layers were evaluated through write–read–erase–read (WRER) cycles. After applying writing and erasing gate bias, the ON and OFF currents were steady. The ON/OFF current ratios of TPA(PDAF)*_n_* (*n* = 1, 2, 3) based devices were measured around 10^2^–10^3^ during the WRER examinations, and the devices were tested for over 100 cycles (Figure S16, Supporting Information). The retention time is a critical measure of charge storage stability that reflects the duration of the stored charge retained in the charge‐trapping layer. The retention time of the ON and OFF states of the devices at a gate voltage of −35 V was maintained for 10^4^ s with ON/OFF current ratios of 11, 29, and 522, respectively (Figure 5c). The relative higher ON/OFF current ratio of TPA(PDAF)_3_ was mainly attributed to its higher ON current state which was attributed to the high mobility. As shown in Figure 5c, in initial period, the rapid degradation of the reading *I*_DS_ at both programmed and erased states was observed due to the release of the charges.^17^ After the charge equilibrium was reached, the reading *I*_DS_ maintained slightly reduced stability. The degradations of *I*_DS_ for TPA(PDAF)*_n_* devices exhibited nonexponential characteristics, which were consistent with the model for long‐term trapped charge decay in most polymer/small molecule elements.[[qv: 3a,8a]] For the erasing process, when the reverse gate bias and light irradiation were applied, the trapped holes were neutralized by the injected photoinduced electrons, resulting in a high conductance state. Due to linking three diazafluorene units which exhibited strong electron affinity, TPA(PDAF)_3_ possessed the strongest electron trapping ability. The trapped photogenerated electrons in TPA(PDAF)_3_ were more stable than those in TPA(PDAF)_1_ and TPA(PDAF)_2_. So, the erasing state of TPA(PDAF)_3_ remained relatively stable compared with the TPA(PDAF)_1_ and TPA(PDAF)_2_ devices. In consideration of the programmed state, the *I*_DS_ of TPA(PDAF)_3_ exhibited better stability than those of TPA(PDAF)_1_ and TPA(PDAF)_2_. One possible reason is that in the TPA(PDAF)_3_ molecule, the TPA core surrounding by three strong electron‐withdrawing 4,5‐diazafluorene groups, which naturally acts as hole‐blocking role, could trap more holes and prevent the stored charge from leaking. The schematic of molecular charge storage mechanism is revealed in Figure 5d. Under gate electric field, the holes were injected into the HOMO of the molecules and trapped in the TPA moieties. With the increased hole blocking diazafluorene groups, holes were harder released from TPA cores, thus, more stable retention characteristics of TPA(PDAF)_3_ element were found. Additionally, owing to three bulky diazafluorene moieties connected to TPA, the larger torsion angle of TPA(PDAF)_3_ caused high barrier for trapping holes deeply and prevented the leakage of stored holes leading to long‐time charges reserved in the trapping layer.^10^, ^21^ Furthermore, according to results of the spectra property and theory calculation, TPA(PDAF)_3_ film presented single nanomolecular behavior and random molecular distribution, which could be viewed as isolated molecular trapping centers with minor charge delivery. The special behavior was also helpful for charge storage and maintenance. This result reflects the importance of the electronic structure of the charge storage elements on the memory characteristics.

### 2.6 Device Optimization

In order to obtain better retention property and ON/OFF current ratio, we blend TPA(PDAF)*_n_* (*n* = 1, 2, 3) into PS for optimized charge trapping layers. As is well‐known, PS is hydrophobic and has a low‐k, which benefits smooth thin‐film charge trapping elements with pretty good mobility. Besides, PS acted as “charge blocking” layers improving the device stability by blocking the charges release, owing to the lower HOMO level of PS (−5.60 eV) and provided a deeper trapping site[[qv: 8b]] that would avoid the charge escaping from the trapping layers.^28^ First, OFET memory devices based on different wt% ratios (0%, 5%, 10%, 20%, 30%, and 40%) of TPA(PDAF)_3_ blended in PS serving as charge trapping layers were fabricated to optimize a best blending ratio. The transistor and memory characteristics of various wt% TPA(PDAF)_3_@PS were shown in Table S2 (Supporting Information). With the blending ratio increased, more trapping sites were provided due to the increasing TPA(PDAF)_3_, but the hole mobility was decreased which reduced the hole injected from pentacene into TPA(PDAF)*_n_*.^17^ The ideal negative memory window was found with the blending ratio of 10–20%. The positive memory windows with different blending ratios showed little change, which suggested that the parameter value is mainly determined by the intrinsic properties of the TPA(PDAF)_3_. The ON/OFF ratio of 10% TPA(PDAF)_3_@PS device was almost two orders higher than those with other blend ratios devices. Thus, blending ratio of 10% was chosen to optimize the TPA(PDAF)*_n_* (*n* = 1, 2, 3) elements.

**Figure 6:**
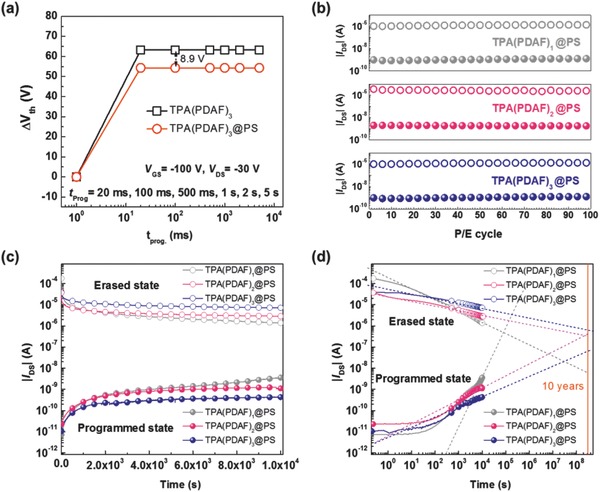
a) Corresponding shifts of threshold voltage (Δ*V*_th_) as a function of applied programming time (*t*_prog_) for both TPA(PDAF)_3_ and TPA(PDAF)_3_@PS devices. b) Endurance characteristics and c) retention characteristics of TPA(PDAF)*_n_*@PS based transistor memories (the currents at ON and OFF states were read at *V*_G_ = −35 V and *V*_D_ = −30 V). d) Extended fitting curves of retention time to 10^9^ s.

As Figure S17 (Supporting Information) showed, the transfer characteristics of the optimized 10 wt % TPA(PDAF)*_n_*@PS devices exhibited much higher field‐effect mobilities of 0.20, 0.25, and 0.55 cm^2^ V^−1^ s^−1^, respectively. The drain–source currents of TPA(PDAF)*_n_*@PS based OFETs enhanced dramatically, which benefit larger ON/OFF current ratios of 10^6^, 10^7^, and 10^7^, respectively (see Table 2). Predictably, the blend elements exhibited similar negative memory windows for holes storage compared with isolated TPA(PDAF)*_n_* based devices. As Figure S18 (Supporting Information) and Table 2 showed, when applying negative gate bias from −60 to −80 V, negative memory windows of 17.83 and 24.75 V for TPA(PDAF)_1_, 25.43 and 33.98 V for TPA(PDAF)_2_, and 25.46 and 38.30 V for TPA(PDAF)_3_ were found. Slightly smaller memory windows occurred comparing with isolated TPA(PDAF)*_n_* based devices, which were ascribed to the reduced trapping sites in the blend elements. For TPA(PDAF)*_n_*@PS based devices, under −100 V programming process, when the programming time was changing from 20 ms to 5 s, the memory windows were also not changed, which revealed the fast trapping efficiency in TPA(PDAF)*_n_* (**Figure** **6**a; Figure S19, Supporting Information). The positive memory windows were 1.15 V for TPA(PDAF)_1_, 17.4 V for TPA(PDAF)_2_, and 27.0 V for TPA(PDAF)_3_ under a positive gate bias of 80 V with assistance of light for 1 s. Whether blend with PS or not, the role of 4,5‐diazafluorene group for electron trapping, high charge trapping density, and preventing hole leakage is still obvious. The ON/OFF current ratios of the three devices were indeclinable along with 100 WRER cycles (Figure 6b). Besides, the retention characteristics were obviously enhanced when blending TPA(PDAF)*_n_* in PS. The retention time of the optimized devices at a reading gate voltage of −35 V was still maintained over 10^4^ s with a high ON/OFF current ratio of over 10^3^ for TPA(PDAF)_1_ and TPA(PDAF)_2_. The ON/OFF current ratio of TPA(PDAF)_3_ was even up to 1.9 × 10^4^, as shown in Figure 6c. The TPA(PDAF)_2_ and TPA(PDAF)_3_ based memories exhibited good extrapolated retention characteristics, as Figure 6d illustrated. Further extending the fitting curves to ten years, the TPA(PDAF)_3_ based memory still had an ON/OFF current ratio greater than 10.^29^ This long retention time indicated that TPA(PDAF)*_n_*@PS are promising elements for reliable and stable memory devices used in high density information storage with controllable charge behaviors.

## 3 Conclusion

In summary, we have successfully synthesized three diazafluorene derivatives TPA(PDAF)*_n_* (*n* = 1, 2, 3) serving as molecular charge storage elements, which possess D–A type conjugate interrupted structures with steric hindrance building blocks. With fully end‐capping steric hindrance of triphenylamine, TPA(PDAF)_3_ owns highly nonplanar topological configuration and weaker intermolecular interaction, which suppress π–π stacking and aggregation in the solid states. The complete separation of HOMO/LUMO of diazafluorene derivatives facilitates the balance of carrier injecting and transporting and offers ideal hole and electron trapping sites. The OFET memory devices based on solution‐processing TPA(PDAF)*_n_* (*n* = 1, 2, 3) elements were examined and TPA(PDAF)_3_ exhibited the best memory performance. With increased diazafluorene units, the larger memory windows were observed, whether for hole storage or electron storage, which illustrated that diazafluorene groups not only provided electron trapping sites, but also acted as hole blocking role for trapping more holes deeply in triphenylamine and prevented charge leakage. TPA(PDAF)_3_ based device showed an ambipolar memory behavior with a large memory window of 89 V, high hole trapping density and fast trapping speed. Optimizing by mixing with 90% PS, the device exhibited reliable endurance property and good charge retention time. The novel donor–acceptor small molecular elements design of trapping core attached with diazafluorene is a promising candidate for high performance OFET memory.

## 4 Experimental Section

*Chemical and Materials*: All the solvents and reagents were purchased from commercial suppliers and used without further purification, unless noted otherwise. All products were purchased by flash column chromatography which was carried out with silica gel (200–300 mesh). Spectrochemical‐grade solvents were used for optical measurements. Bromobenzene and 1,10‐Phenanthroline monohydrate, triphenylamine were obtained from Aldrich Chemical Co. Potassium carbonate, magnesium sulphate, chloroform, and toluene were purchased from Suzhou Rosen Chemical Co., Ltd. without further purification. Dichloromethane was dried by anhydrous sodium under room temperature. Tetrahydrofuran (THF) and toluene were dried over sodium benzophenone ketyl anion radical and distilled under a dry nitrogen atmosphere immediately prior to use. DAFO was synthesized as in our previous work.^22^ Pentacene, PS (weight average molecular weight, *M*_w_ = 250 000) were purchased from Sigma‐Aldrich and used without further purification. Experimental details including general methods, synthesis, NMR, HRMS, theoretical calculations, and crystallographic data are given in the Supporting Information.

*Characterization*: All compounds were characterized by ^1^H NMR and ^13^C NMR on a Bruker AVANCE III 400 MHz instrument with tetramethylsilane as the internal standard. For the high‐resolution mass spectrometry, the data were recorded on Thermo Fisher Scientific LTQ FTICR‐MS with MALDI_DHB positive ion mode. X‐ray diffraction was performed on a Bruker D8 X‐ray diffractometer with Mo Ka radiation (λ = 0.71073 Å), and the operating 2θ angle ranged from 5° to 50°, with the step length of 0.02°. Absorption spectra were measured with a Shimadzu UV‐3150 spectrometer and emission spectra were recorded on a Shimadzu RF‐530XPC luminescence spectrometer. DSC analyses were performed on a Shimadzu DSC‐60A Instrument. TGAs were conducted on a Shimadzu DTG‐60H thermogravimetric analyzer under a heating rate of 10 °C min^−1^ and a nitrogen flow rate of 20 cm^3^ min^−1^. CV studies were conducted using an CHI600C in a typical three‐electrode cell with a platinum sheet working electrode, a platinum wire counter electrode, and a silver/silver nitrate (Ag/Ag^+^) reference electrode. AFM measurements were obtained with a NanoScope IIIa AFM at room temperature. Commercial silicon cantilevers with typical spring constants of 21–78 N m^−1^ were used to operate the AFM in tapping mode. All the quantum‐chemical calculations were performed using the Gaussian 09 program suite.

*Device Fabrication and Characterization*: OFET memory devices were fabricated with a top‐contact and bottom‐gate configuration. Heavily doped n‐type Si wafer with 300 nm SiO_2_ as the gate dielectric (15 nF capacitance) was used as the substrate. The substrates were cleaned sequentially in an ultrasonic bath with acetone, ethanol, and deionized water for 10 min each, and then transferred into an oven at 120 °C for 30 min after dried using a nitrogen gun. Later, the substrate surface was UV/ozone cleaned for 3 min and transferred to a N_2_ filled glovebox. Chloroform solution of TPA(PDAF)*_n_* and TPA(PDAF)*_n_*@PS (5 mg mL^−1^) were stirred 30 min to form a homogeneous solution. All the solution‐processed single‐component films were fabricated by spin‐coating, with the spin speed of 2500 rpm for TPA(PDAF)_1_, 2900 rpm for TPA(PDAF)_2_, 3000 rpm for TPA(PDAF)*_n_*, and 3000 rpm for TPA(PDAF)*_n_*@PS, respectively. The spin time was 30 s. Then, they were baked at 80 °C on a hot plate for 30 min to remove the residual solvent. The thicknesses of TPA(PDAF)*_n_* thin films were estimated to be 22.3, 20.8, and 20.3 nm, respectively. The thicknesses of (0–40 wt%) TPA(PDAF)_3_@PS films were 37.2, 33.6, 33.9, 34.2, 36.7, and 35.0 nm, respectively. The thicknesses of TPA(PDAF)*_n_* (*n* = 1, 2)@PS films were 35.5 and 34.2 nm. After that, 50 nm thick pentacene was thermally evaporated at a deposition rate of 0.1 Å s^−1^ under a pressure of 5 × 10^−4^ Pa. Finally, about 50 nm thick Au was thermally evaporated through a shadow mask to form source and drain electrodes with the channel width *W* = 1500 µm and length *L* = 100 µm. Film thickness was measured by Bruker Dektak XT stylus profiler. The electrical characteristics of the memory devices were characterized using an Keithley 4200‐SCS semiconductor parameter analyzer. All electrical measurements were carried out under ambient conditions at room temperature.

## Conflict of Interest

The authors declare no conflict of interest.

## Supporting information

Supplementary
