# Supplementary material for: 4,5‐Diazafluorene‐Based Donor–Acceptor Small Molecules as Charge Trapping Elements for Tunable Nonvolatile Organic Transistor Memory
Source: Adv Sci (Weinh). 2018 Sep 6;5(12):1800747. doi: 10.1002/advs.201800747 (PMC6299726; doi:10.1002/advs.201800747)
Supplement: Supplementary file 1 — Supplementary [file ADVS-5-1800747-s001.pdf]

Copyright WILEY-VCH Verlag GmbH & Co. KGaA, 69469 Weinheim, Germany, 2018.

Supporting Information

for *Adv. Sci.,* DOI: 10.1002/advs.201800747

4,5-Diazafluorene-Based Donor–Acceptor Small Molecules as Charge Trapping Elements for Tunable Nonvolatile Organic Transistor Memory

Yang Yu, Lin-Yi Bian, Jian-Guo Chen, Qi-Hao Ma, Yin-Xiang Li, Hai-Feng Ling, Quan-You Feng, Ling-Hai Xie, Ming-Dong Yi, and Wei Huang**

WILEY-VCH

Copyright WILEY-VCH Verlag GmbH & Co. KGaA, 69469 Weinheim, Germany, 2016.

Supporting Information

4,5-Diazafluorene-based Donor-Acceptor Small Molecules as Charge Trapping Elements for Tunable Nonvolatile Organic Transistor Memory

Yang Yu, Lin-Yi Bian, Jian-Guo Chen, Qi-Hao Ma, Yin-Xiang Li, Hai-Feng Ling,

Quan-You Feng, Ling-Hai Xie*, Ming-Dong Yi, and Wei Huang*

General Methods. Unless otherwise information noted, all reagents were obtained from commercial source and used without purification further. Most of products were purified via column chromatography over silica gel (200-300 mesh) and some of them were purified by recrystallization. ^{1}H and ^{13}C NMR data were received from a Bruker 400 MHz NMR Fourier transform spectrometer (400 MHz and 100 MHz, respectively) at 20 ^{o}C. Chemical shifts are shown as δ in units of parts per million (ppm) relative to internal standard (^{1}H NMR: tetramethylsilane (TMS) = 0.00 ppm) or relative residual peaks (^{1}H NMR: 7.26 for $CDCl_3$, 2.50 for d_6-DMSO; ^{13}C NMR: 77.0 triplet for $CDCl_3$, 39.25 for d_6-DMSO). Multiplicities of every signal peak in briefly were shown as: s (singlet); d (doublet); t (triplet); q (quartet); dd (doublet of doublets); dt (doublet of triplets); m (multiplet). Coupling constants are expressed as a *J* value in Hz. HRMS were used as the determination of molecular weight.

X-ray crystallographic data. Crystallographic data for compound 3 and compound 6 were saved as the files of CCDC 974323.cif and 974342.cif, respectively. Data were collected at room temperature on a detector with graphitemonochromated Mo Ka radiation (λ=0.71073 Å). The absorption correction was applied by integration based on the crystal shape. Structures were solved by direct methods and refined against F2 with the full-matrix and least-squares methods. Hydrogen atoms were found by difference Fourier syntheses and were refined.

Crystal data for compound 3 (TPDAFOH). $C_{29}H_{20}O_1N_2$, M=412.49, T=298 K, monoclinic, C 2/c, a=22.3007(39) Å, b=6.7956(12) Å, c=28.9475(51) Å, β=101.973(2)°, V=4291.46(131) Å^{3}, ρ_{calcd} =1.27679 Mg m^{-3}, F(000)=1728, h, k, l_{max}=26, 8, 34, N_{ref}=3765, T_{min}, T_{max}=0.955, 0.975, $2\theta_{max}$= 50°, R(reflections)=0.0497(3302), R2(reflections)=0.2014(3754), S=1.493, N_{par}=291, Data completeness=0.997.

Crystal data for compound 6 (TPA(PDAF)$_3$). $C_{69}H_{45}N_7$, M=972.12, T=173(2) K, orthorhombic, Pna21(33) , Z=4, a=26.5050(16)Å, b=17.2404(8)^{o}, c=12.7703(8)^{o}, a=90.00^{o}, b=90.00^{o}, g=90.00^{o} Å, V=5835.48(58) Å^{3}, F(000)=2032, c_{lad}=1.107Mg m^{-3}, $2\theta_{max}$=50. 7°, R(reflections)=0.0723(5733), h, k, l_{max}=31, 20, 15, N_{ref}=10684[5602],

T_{min}, T_{max}= 0.955, 0.975, wR2(reflections)=0.2062(9834), S=1.043, N_{par}=686, Data completeness=1.76/0.92.

Crystal data for CCDC 974342 contained the supplementary crystallographic data for this paper. These data can be obtained free of charge from The Cambridge Crystallographic Data Centre via www.ccdc.cam.ac.uk/data_-request/cif.

Cyclic Voltammetry. CV were conducted at room temperature on the CHI660E system in a typical three-electrode cell with a platinum sheet working electrode, a platinum wire counter electrode, and a silver/silver nitrate (Ag/Ag^+) reference electrode. All electrochemical experiments were carried out under a nitrogen atmosphere at room temperature and performed on the solution of the sample on a glassy carbon electrode measured in Bu_4NPF_6 (0.1 M)/acetonitrile at a sweeping rate of 0.1V/s. According to the redox onset potentials of the CV measurements, the highest occupied molecular orbital (HOMO)/lowest unoccupied energy levels (LUMO) of the materials are estimated based on the reference energy level of ferrocene (4.8 eV below the vacuum). HOMO/ LUMO = - ($E_{ox/red}$ - 0.09 V) + 4.8 eV, where the value 0.09 V is the standard potential for ferrocene/ferrocenium vs Ag/Ag^+.

Theoretical calculations. The electronic ground states of all the compounds were computed by Becke's three-parameter density functional in combination with Lee Yang Parr's correlation functional (B3LYP) utilizing 6-31G(d) basis sets.27-28 The ground-state geometries of all the compounds were adequately optimized at the B3LYP/6-31G(d) level. TDDFT/B3LYP/6-31G(d) calculations of the excitation energies were then performed at these optimized geometries. All computations were performed using the Gaussian 09 package.

General synthesis of six compounds

Synthetic procedures of 9-phenyl-4,5-diazafluorene-9-ol (compound 1),
1,9-diphenyl-4,5 -diazafluorene-9-ol (compound 2) and
1,8,9-triphenyl-4,5-diazafluorene-9-ol (compound 3) by Grignard reaction.

The synthetization of phenyl magnesium bromide solution: Magnesium turnings and a piece of iodine were added into a 100 ml flask under a N_2 atmosphere. Then, 2-3 drops of bromobenzene and 5 ml tetrahydrofuran (THF) were added into the flask. The reaction was triggered by heated to a suitable temperature, meanwhile the color of reaction liquid turned from orange to transparent. Then remained bromobenzene was added into the flask with some other THF in ice-bath. Ultimate, phenyl magnesium bromide solution can be obtain after 2 h of reflux in oil-bath (55 ^{o}C).

4,5-diazafluorene-9-one (DAFO), which was synthesized as the method of precious work of our group,[22] was added into 500 ml flask under a N_2 atmosphere. Then 200 ml tetrahydrofuran (THF) was injected into the flask to dissolve the DAFO in Acetone dry ice bath (-78 ^{o}C) for compound 1. Synthesis methods of compound 2 and compound 3 were the same as compound 1 except the reaction temperature (-10 ^{o}C for compound 2 and 40 ^{o}C for compound 3). Phenyl magnesium bromide solution was injected into the flask containing DAFO after extracted from its flask by injection

syringe. The reaction can be quenched by Saturated ammonium chloride solution after stirring for 15min. the reaction mixture was extracted with CH_2Cl_2 and dried with $MgSO_4$. The obtained organic phase was condensed by rotary evaporation under reduced pressure and the crude residue was subjected to flash column chromatography with ethyl acetate (EA) and petroleum ether to obtain the target products.

Compound 1: yield 91%. White solid. ^{1}H NMR (400 MHz, d_6-DMSO): δ (ppm) 8.63 – 8.64(d, J = 6.4 Hz, 2H), 7.73 – 7.71 (d, J = 9.2 Hz 2H), 7.36 – 7.33 (m, J = 12.8 Hz 2H), 7.27 – 7.23 (m, J = 24.4 Hz, 5H), 6.68 (s, 1H). ^{13}C NMR (100 MHz, d_6-DMSO): δ (ppm) 157.58, 150.89, 146.68, 143.39, 132.97, 128.86, 127.83, 125.63, 124.66, 79.22. HRMS: m/z calcd for [M+H$^+$] $C_{17}H_{13}O_1N_2$: 261.1022; found: 261.1021.

Compound 2: yield 53%. White solid. ^{1}H NMR (400 MHz, d_6-DMSO): δ (ppm) 8.71 – 8.70 (d, J = 5.2 Hz,1H), 8.63 – 8.62 (d, J = 4.8 Hz, 1H), 7.53 – 7.51 (d, J = 7.6Hz, 1H), 7.37 – 7.35 (d, J = 7.2, 2H), 7.32 – 7.31 (m, J = 7.2, 1H), 7.28 – 7.27 (d, J = 12 Hz, 1H), 7.20 – 7.19 (d, J = 4.8 Hz, 1H), 6.98 – 6.97 (m, J =5.6 Hz, 3H), 6.93 – 6.92 (d, J = 4.8 Hz 2H), 6.81 (s, 1H). ^{13}C NMR (100 MHz, (d_6-DMSO): δ (ppm) 158.74, 156.76, 151.14, 150.88, 148.18, 147.49, 143.08, 141.59, 137.34, 132.48, 129.52, 128.45, 128.09, 127.93, 127.10, 125.69, 125.36, 124.92, 79.88. HRMS: m/z calcd for [M+H$^+$] $C_{23}H_{16}N_2O$: 336.1272; found: 336.1270.

Compound 3: yield 77%. White solid. ^{1}H NMR (400 MHz, d_6-DMSO): δ (ppm) 8.73 – 8.72 (d, J = 5.2 Hz, 2H), 7.23 – 7.08 (m, 12H), 7.05 (s, 1H), 6.75-6.72 (dd, 1H), 6.64 – 6.62(dd, 2H), 6.51 – 6.49 (d, J = 4.8 Hz, 2H). ^{13}C NMR (100 MHz, $CDCl_3$): δ (ppm) 157.20, 150.84, 148.30, 142.91, 139.75, 136.71, 128.88, 127.94, 127.57, 127.04, 126.31, 125.63, 125.10, 81.46. HRMS: m/z calcd for [M+H$^+$] $C_{29}H_{20}N_2O$: 412.1576; found: 412.1579.

General procedure for Friedel–Crafts reaction

9-phenyl-4,5-diazafluorene-9-ol and triphenylamine (TPA) were added into a 500 mL flask and stirred to dissolve in dichloromethane (DCM) in room temperature. Then, sulfuric acid was added as a catalyst into the solution and refluxed for 6 h. Then neutralized the solution with NaOH (aq) to pH at about 8. After stirring for another 1 h to neutralize adequately, the reaction mixture was extracted with CH_2Cl_2 and dried with $MgSO_4$. The obtained organic phase was condensed by rotary evaporation under reduced pressure and the crude residue was subjected to flash column chromatography with ethyl acetate (EA) and petroleum ether to obtain the target product, which was offwhite solid.

Synthesis of *N*,*N*-diphenyl-4-(9-phenyl-4,5-diazafluorene-9-yl)phenylamine (**$TPA(PDAF)_1$**) (compound 4)

Following the above procedures, 9-phenly-4,5-diazafluoren-9-ol (0.26 g, 1 mmol) and triphenylamine (0.74 g, 3 mmol) were added into a 250 mL flask and stired to dissolve in 150 mL DCM. Then, 2.5 mL sulfuric acid as catalyst was added into the solution and refluxed for 6 h. Then neutralized the solution with NaOH (aq) to pH at

about 8. After stirring for another 1 h to neutralize adequately, the reaction mixture was extracted with CH_2Cl_2 and dried with $MgSO_4$. The obtained organic phase was condensed by rotary evaporation under reduced pressure and the crude residue was subjected to flash column chromatography with ethyl acetate (EA) and petroleum ether to obtain the target product of **TPA(PDAF)$_1$**. Offwhite solid. 273 mg. 0.56 mmol. Yield: 56%. ^{1}H NMR (400 MHz, $CDCl_3$): δ (ppm) 8.73 – 8.72 (dd, *J* = 4.7 Hz, 1.2 Hz, 2H), 7.79 – 7.77 (dd, *J* = 7.8 Hz, 1.3 Hz, 2H), 7.29 – 7.27 (dd, *J* = 7.6 Hz, 4.8 Hz, 2H), 7.25 – 7.18 (m, 9H), 7.07 – 7.05 (d, *J* = 7.6 Hz, 4H), 7.03 – 6.98 (m, 4H), 6.92 – 6.90 (d, *J* = 8.7 Hz, 2H). ^{13}C NMR (100 MHz, $CDCl_3$): δ (ppm) 157.4, 150.1, 147.4, 147.1, 146.0, 143.7, 136.6, 133.8, 129.3, 128.6, 127.8, 127.4, 124.7, 123.5, 123.2, 122.7, 61.1. HRMS: m/z calcd for [M+H$^+$] $C_{35}H_{26}N_3$: 488.2121; found: 488.2120.

Synthesis of *N*,*N*-bis(4-(9-phenyl-4,5-diazafluorene-9-yl)phenyl)phenylamine (**TPA(PDAF)$_2$**) (compound 5) and tris(4-(9-phenyl-diazafluorene-9-yl)phenyl)amine (**TPA(PDAF)$_3$**) (compound 6)

According to the general procedure, the two products were obtained by reacting 9-phenly-4,5-diazafluoren-9-ol (0.52 g, 2 mmol) with triphenylamine (0.245 g, 1 mmol) and 9-phenly-4,5-diazafluoren-9-ol (0.94 g, 3.6 mmol) with triphenylamine (0.245 g, 1 mmol), flashing column chromatography with eluent ethyl acetate and petroleum ether; yield 46 % and 75 % of the products TPA(PDAF)$_2$ and TPA(PDAF)$_3$, respectively.

TPA(PDAF)$_2$: Offwhite solid. 336 mg. 0.46 mmol. Yield: 46%. ^{1}H NMR (400 MHz, $CDCl_3$): δ (ppm) 8.72 – 8.71 (d, *J* = 4.7 Hz, 4H), 7.77 – 7.74 (dd, *J* = 7.5 Hz, 0.7 Hz, 4H), 7.28 – 7.21 (m, 13H), 7.17 – 7.15 (m, 4H), 7.05 – 7.03 (d, *J* = 7.6 Hz, 2H), 7.02 – 7.00 (d, *J* = 7.6 Hz, 2H), 6.99 – 6.97 (d, *J* = 8.8 Hz, 2H), 6.89 – 6.97 (d, *J* = 8.7 Hz, 4H). ^{13}C NMR (100 MHz, $CDCl_3$): δ (ppm) 157.4, 150.1, 146.9, 146.7, 145.9, 143.6, 137.2, 133.8, 129.4, 128.6, 127.8, 127.4, 125.1, 123.7, 123.5, 123.3, 61.1. HRMS: m/z calcd for [M+H$^+$] $C_{52}H_{36}N_5$: 730.2965; found: 730.2960.

TPA(PDAF)$_3$: Offwhite solid. 729 mg. 0.75 mmol. Yield: 75%. ^{1}H NMR (400 MHz, $CDCl_3$): δ (ppm) 8.71 – 8.69 (dd, *J* = 4.8 Hz, 1.2 Hz, 6H), 7.74 – 7.72 (dd, *J* = 7.8 Hz, 1.2 Hz, 6H), 7.27 – 7.25 (dd, *J* = 7.6 Hz, 4.8 Hz, 6H), 7.24 – 7.22 (m, 9H), 7.15 – 7.13 (m, 6H), 6.98 – 6.96 (d, *J* = 8.7 Hz, 6H), 6.86 – 6.84 (d, *J* = 8.7 Hz, 6H). ^{13}C NMR (100 MHz, $CDCl_3$): δ (ppm) 157.4, 150.1, 146.2, 145.8, 143.5, 137.9, 133.7, 128.7, 128.6, 127.8, 127.4, 123.8, 123.5, 61.1. HRMS: m/z calcd for [M+H$^+$] $C_{69}H_{46}N_7$: 972.3809; found: 972.3805.

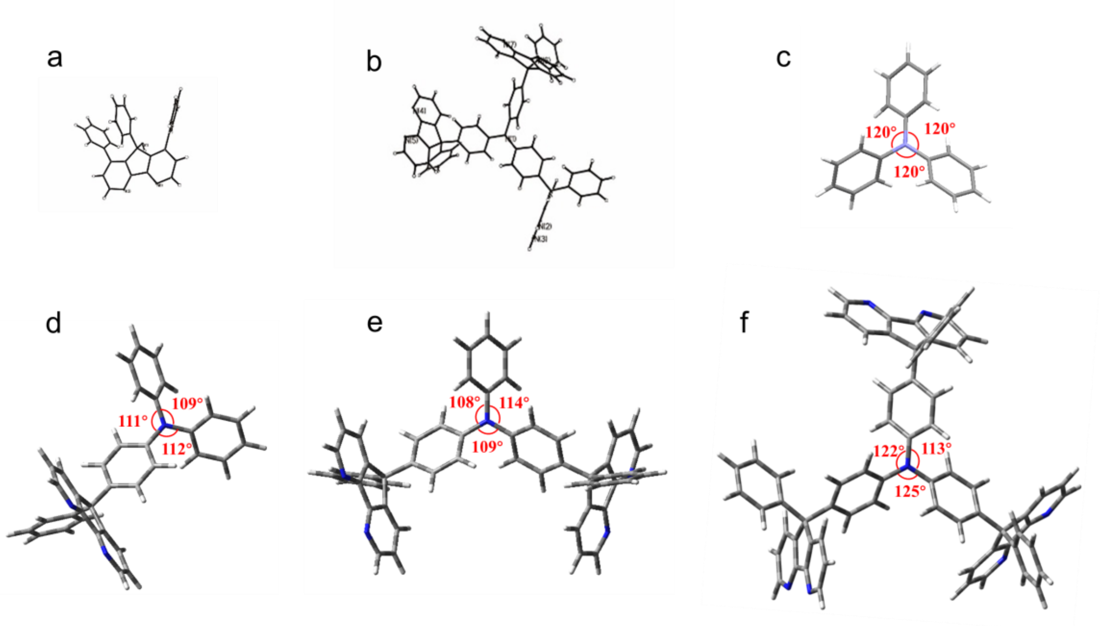

Figure. S1. X-ray single crystal structures of (a) TPDAFOH and (b) TPA(PDAF)$_3$, Angles of (c) TPA, (d) TPA(PDAF)$_1$, (e) TPA(PDAF)$_2$ and (f) TPA(PDAF)$_3$.The single crystal structure of TPA can be referred to Y. X. Li, S. S. Wang, Y. Yu, H. Zhang, W. Y. Wang, R. Q. Yang, L. H. Xie, F. Liu, Z. Q. Lin, N. E. Shi, L. T. Sun, W. Huang, *Small*. **2018**, 14.

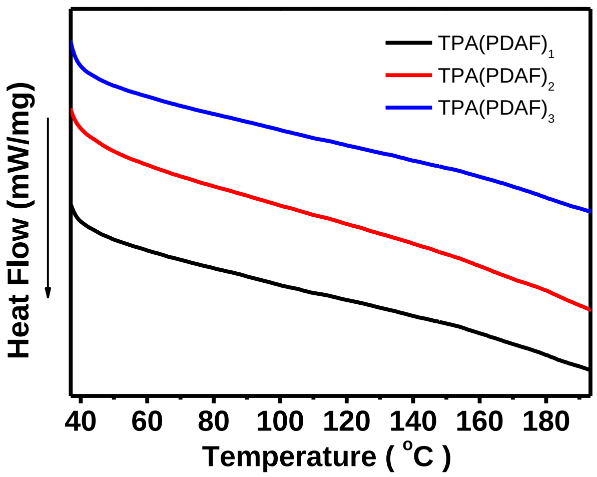

Figure. S2. DSC curve of TPA(PDAF)$_n$ (n=1,2,3) with the heating rate of 10 °C/min in the nitrogen atmosphere.

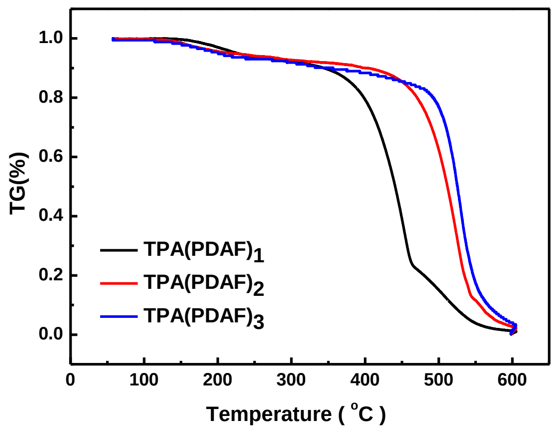

Figure. S3. TGA curve of TPA(PDAF)$_n$ (n=1,2,3) in nitrogen atmosphere.

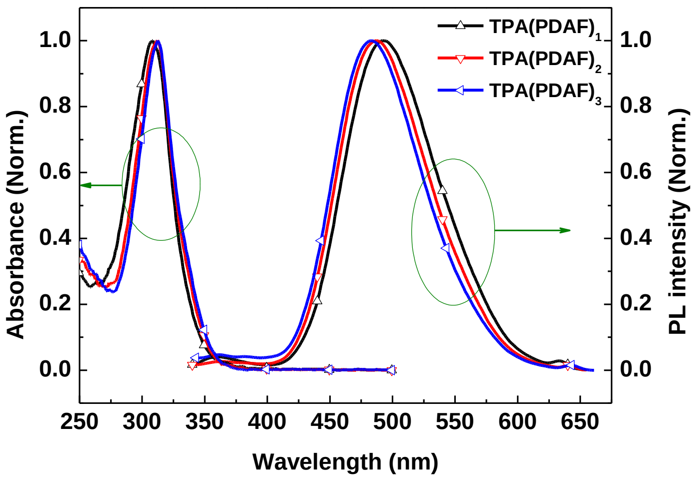

Figure. S4. The absorption and emission spectra of TPA(PDAF)$_n$ (n=1,2,3) in chloroform solutions.

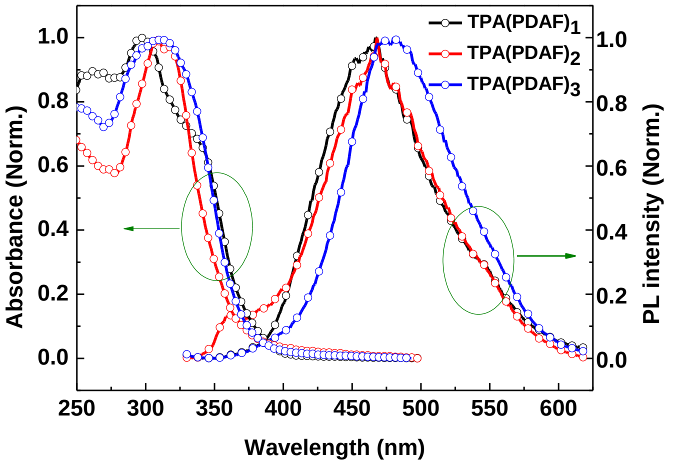

Figure. S5. The absorption and emission spectra of TPA(PDAF)$_n$ (n=1,2,3) in films.

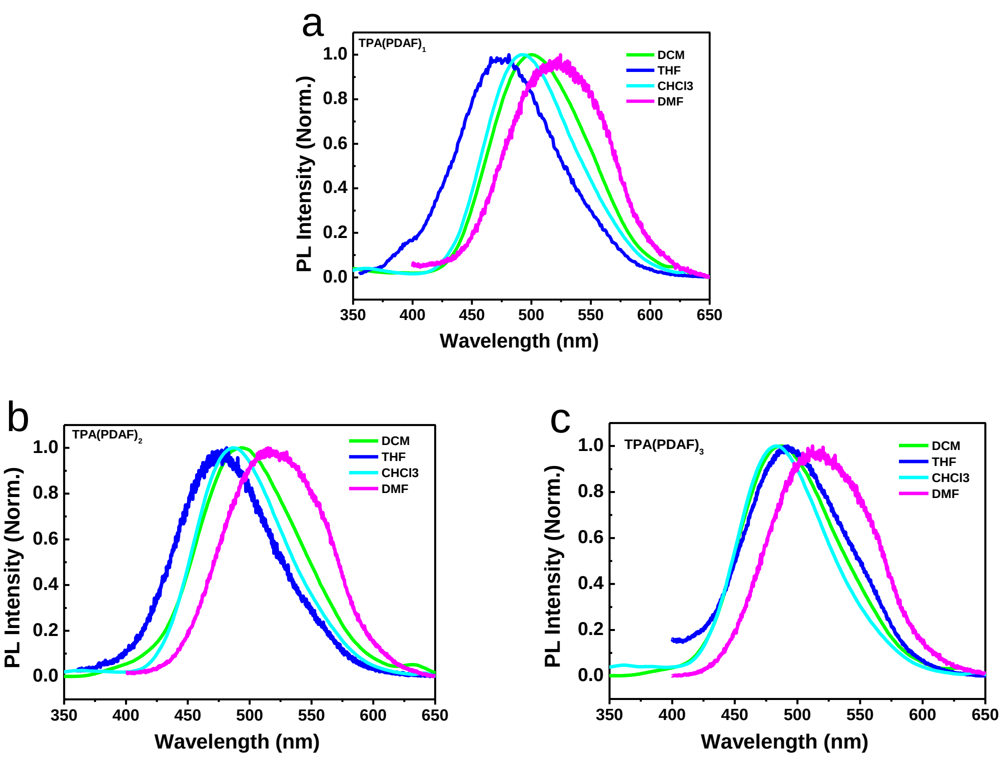

Figure. S6. The PL spectra of the (a) TPA(PDAF)$_1$, (b) TPA(PDAF)$_2$, and (c) TPA(PDAF)$_3$ in different solutions (DCM , $CHCl_3$, THF and DMF).

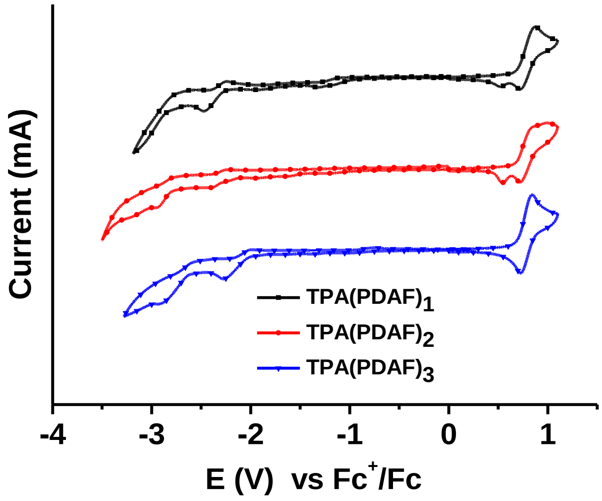

Figure. S7. Cyclic voltammograms of TPA(PDAF)$_n$ (n=1,2,3) in CH_2Cl_2 solution (forward direction) and in THF solution (negative direction). Cyclic voltammogram of the three compounds measured with a scan rate of 100 mV s^{-1} .

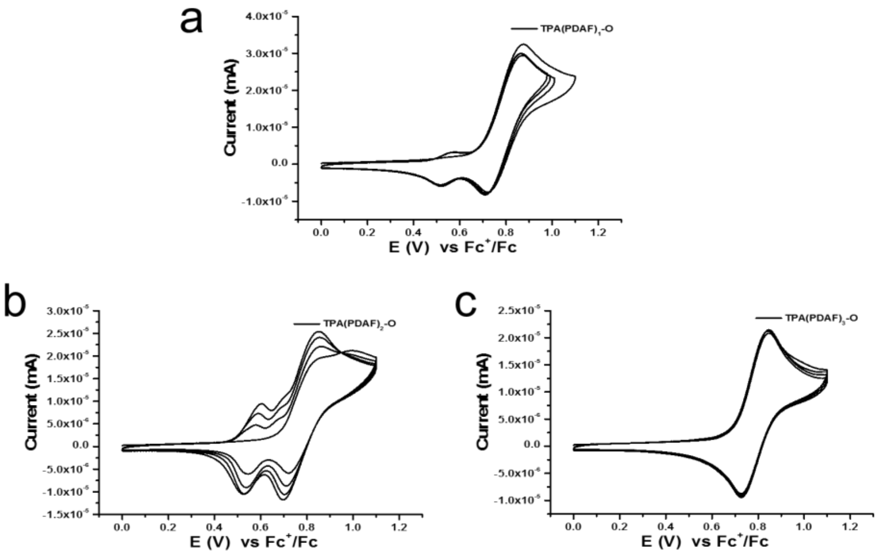

Figure. S8. Cyclic voltammograms of (a) TPA(PDAF)$_1$, (b) TPA(PDAF)$_2$, (c) TPA(PDAF)$_3$ with successive scan curves in the anodic scan.

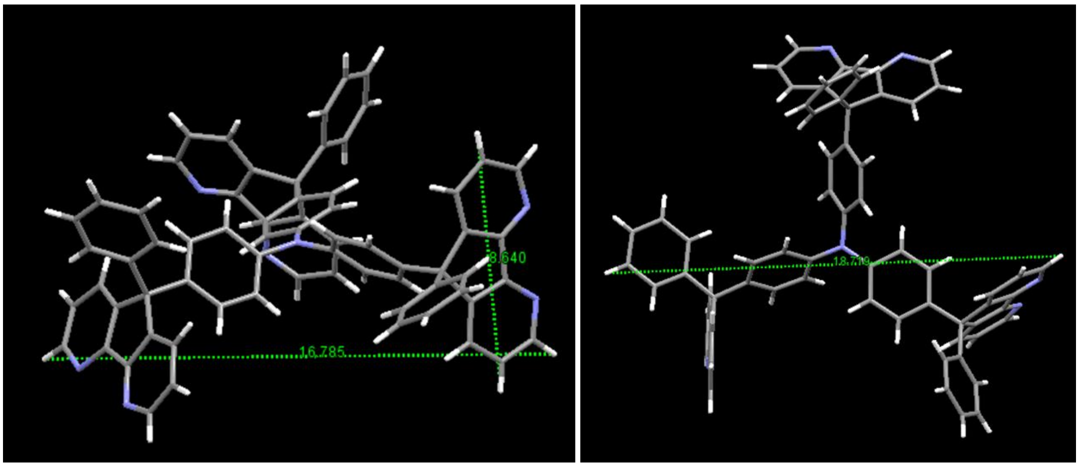

Figure. S9. The 3-dimensional diameter of TPA(PDAF)$_3$ based on the single crystal structure.

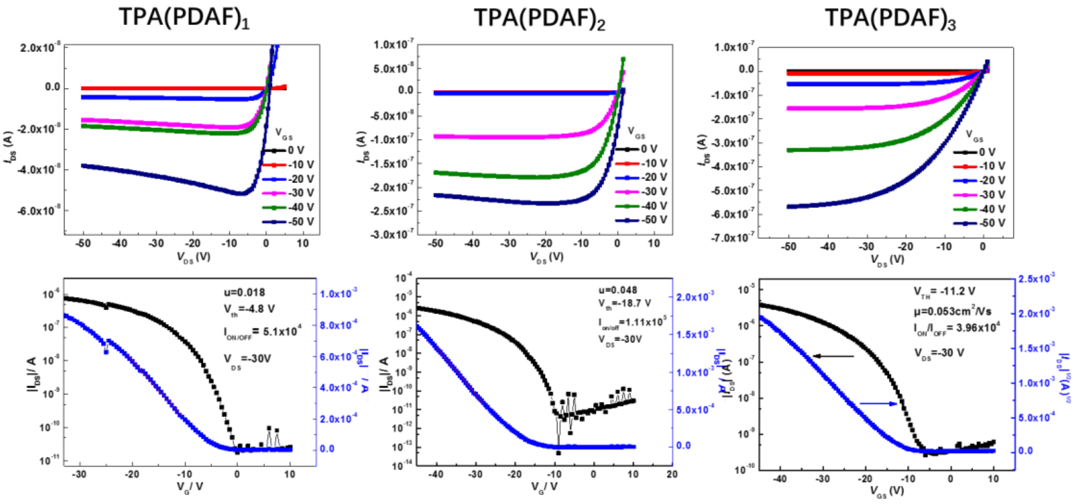

Figure. S10. The output and transfer characteristics of the devices with $TPA(PDAF)_n$ (n=1,2,3) as charge storage layers.

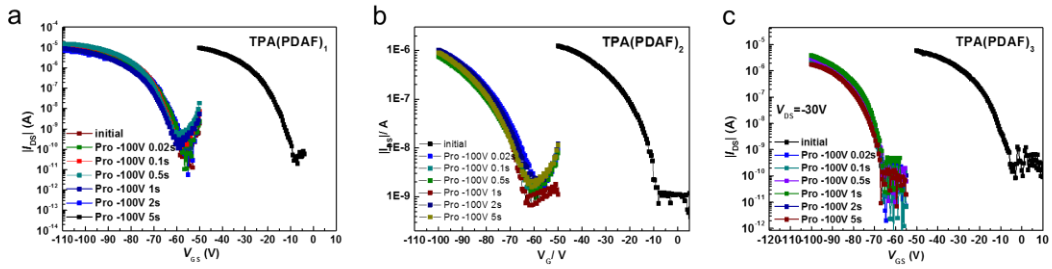

Figure. S11. The transfer characteristics of (a)$TPA(PDAF)_1$, (b)$TPA(PDAF)_2$ and (c)$TPA(PDAF)_3$ based OFET memory. The programming time was changing from 20 ms to 5 s under -100 V gate voltage.

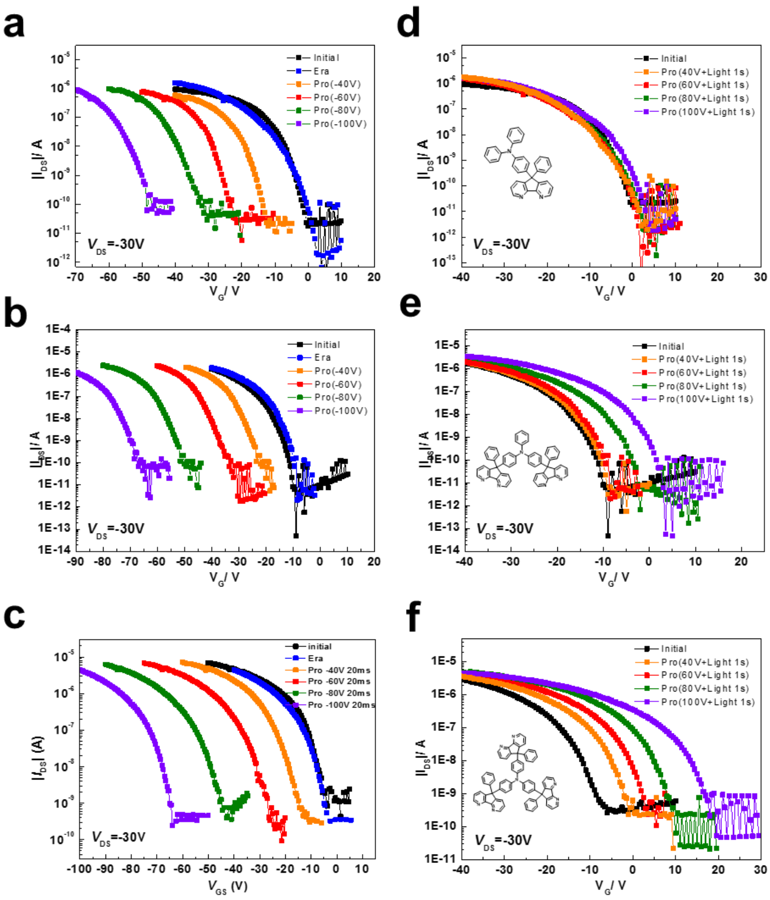

Figure. S12. The transfer curves of the (a) TPA(PDAF)$_1$, (b) TPA(PDAF)$_2$, (c) TPA(PDAF)$_3$ based devices for the programming processes under negative gate voltages ranging from -40, -60, -80 to -100 V. The drain current was measured at V_D = -30 V and the programming time was 20 ms. And (d) TPA(PDAF)$_1$, (e) TPA(PDAF)$_2$, (f) TPA(PDAF)$_3$ based devices for the positive programming processes upon positive gate voltages ranging from 40, 60, 80 to 100 V, with assist of light for 1 s.

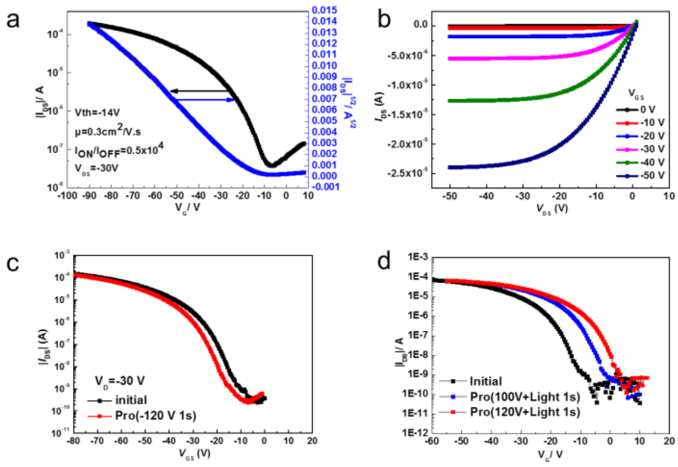

Figure S13. The electrical characteristics of the devices using only diazafluorene as charge trapping layers.

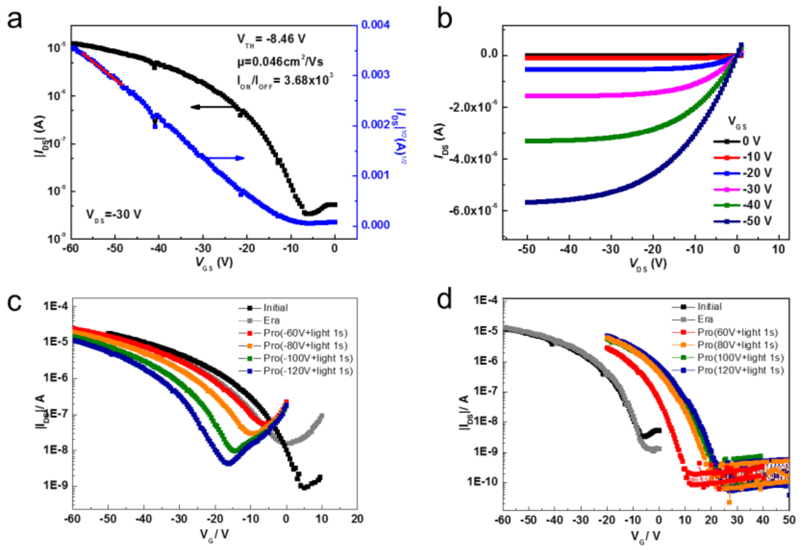

Figure S14. The electrical characteristics of the devices using only triphenylamine as charge trapping layers.

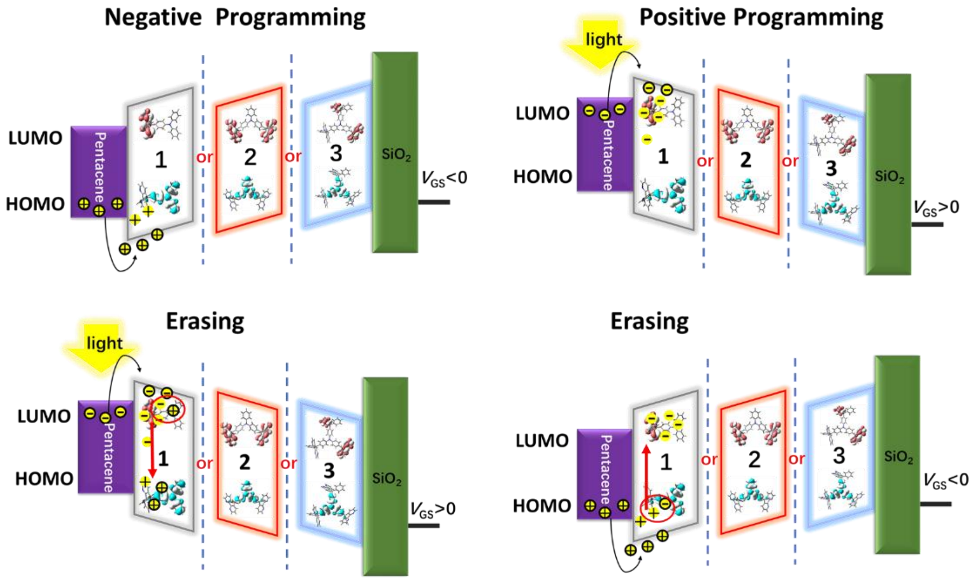

Figure. S15. Energy band diagrams of pentacene and TPA(PDAF)$_n$ under negative and positive gate voltages. (a) hole trapping (PGM mode) (b) electron trapping (PGM mode), (c) electron detrapping and recombination with hole (ERS mode) and (d) hole detrapping and recombination with electron (ERS mode)

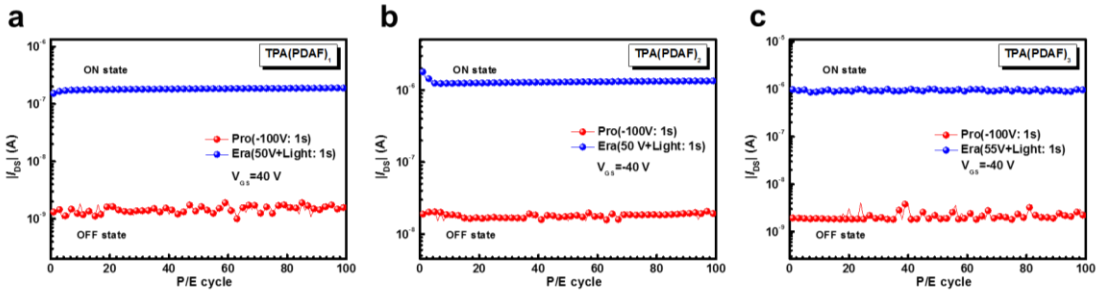

Figure. S16. Write-read-erase-read cycles testing of (a) TPA(PDAF)$_1$, (b) TPA(PDAF)$_2$ and (c) TPA(PDAF)$_3$ based devices.

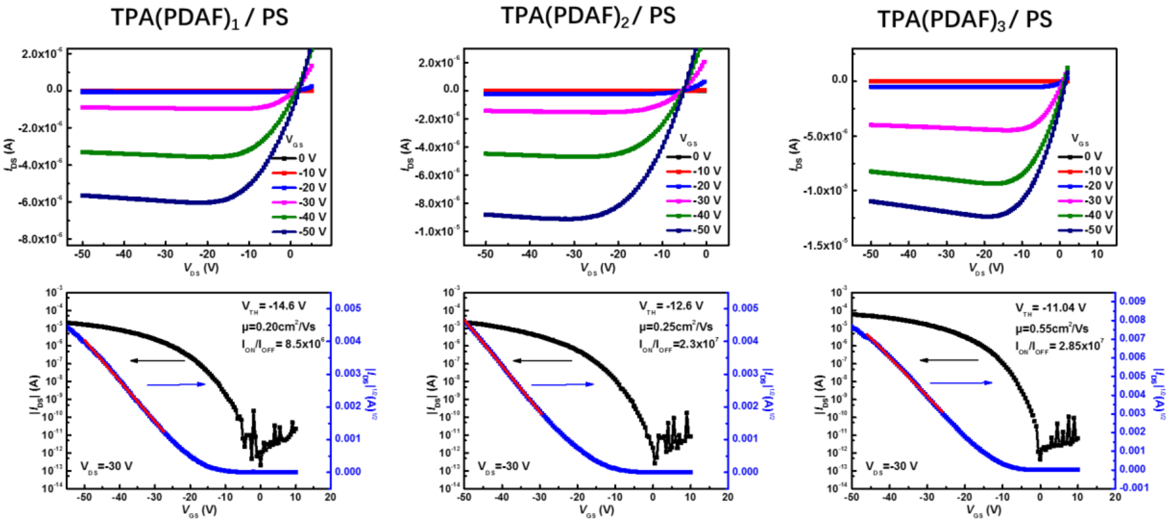

Figure. S17. The output and transfer characteristics of the devices with TPA(PDAF)$_n$ (n=1,2,3) blend with PS as charge storage layers.

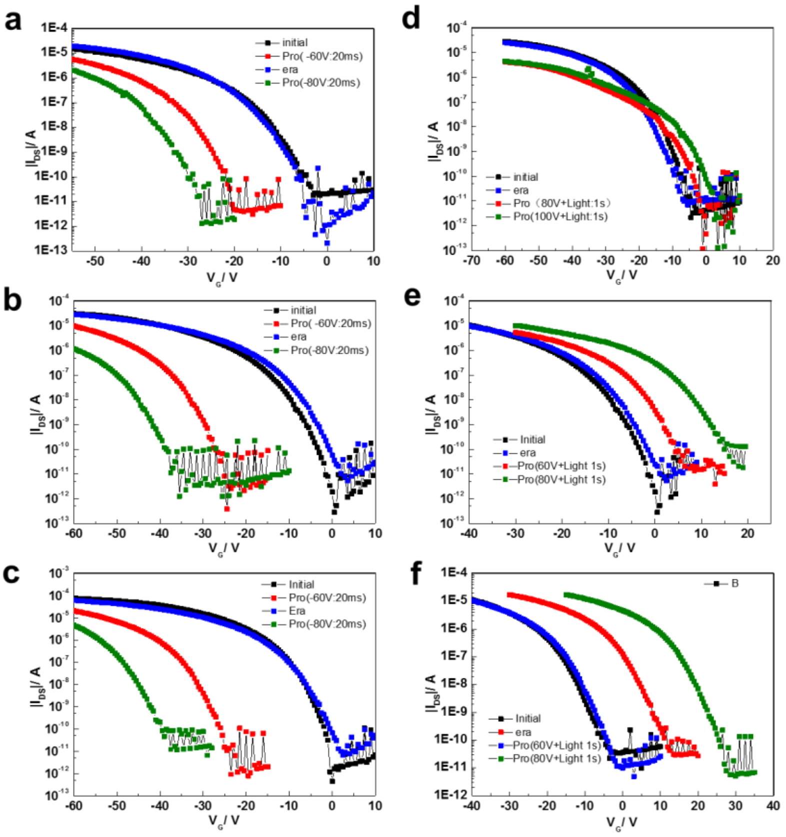

Figure. S18. The transfer curves of the (a) TPA(PDAF)$_1$@PS, (b) TPA(PDAF)$_2$@PS, (c) TPA(PDAF)$_3$@PS based devices for the programming processes under negative gate voltages ranging from -60 to -80 V. The drain current was measured at V_D = -30 V and the programming time was 20 ms. And (d) TPA(PDAF)$_1$@PS, (e) TPA(PDAF)$_2$@PS, (f) TPA(PDAF)$_3$@PS based devices for the positive programming processes upon positive gate voltages ranging from 60 to 80 V, with assist of light for 1 s.

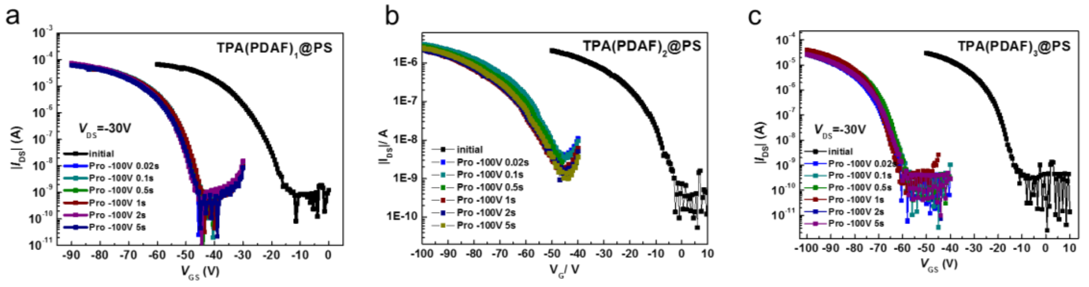

Figure. S19. The transfer characteristics of (a)TPA(PDAF)$_3$@PS, (b)TPA(PDAF)$_2$@PS and (c)TPA(PDAF)$_3$@PS based OFET memory. The programming time was changing from 20 ms to 5 s under -100 V gate voltage.

Table S1. Summary of calculated energies, ionization potential, and electron affinities for TPA(PDAF)$_n$(n=1,2,3)

	Neutral state			Cationic state			Anionic state		
	TPA(PDAF)$_1$	TPA(PDAF)$_2$	TPA(PDAF)$_3$	TPA(PDAF)$_1$	TPA(PDAF)$_2$	TPA(PDAF)$_3$	TPA(PDAF)$_1$	TPA(PDAF)$_2$	TPA(PDAF)$_3$
**Energy**	E_0 = -1513.03327912 a.u.	E_0 = -2276.36912235 a.u.	E_0 = -3039.70489571 a.u.	E_+ = -1512.80280738 a.u.	E_+ = -2276.14051614 a.u.	E_+ = -3039.47772805 a.u.	E_- = -1513.03718143 a.u.	E_- = -2276.38816714 a.u.	E_- = -3039.72996025 a.u.
**Potential Energy**	0	0	0	IP= 6.27 eV	IP= 6.22 eV	IP= 6.18 eV	EA= -0.11 eV	EA= -0.52 eV	EA= -0.68 eV

Table S2. Transistor and memory characteristics of various wt% TPA(PDAF)$_3$ mixed in PS.

Charge Trapping Elements	μ ($cm^2 V^{-1}s^{-1}$)	V_{th}(V)	I_{ON} / I_{OFF}	Negative Window (V)	Positive Window(V)
PS	0.76 ± 0.2	-11.99 ± 0.3	7.1×10^5	0	18.65
5%TPA(PDAF)$_3$@PS	0.56 ± 0.2	-16.19 ± 0.8	2.12×10^6	14.38	21.68
10%TPA(PDAF)$_3$@PS	0.55 ± 0.1	-11.04 ± 1.2	2.85×10^7	38.30	27.0
20%TPA(PDAF)$_3$@PS	0.37 ± 0.5	-12.96 ± 0.8	1.07×10^5	38.69	24.10
30%TPA(PDAF)$_3$@PS	0.25 ± 0.3	-10.35 ± 1.2	4.12×10^5	25.43	27.4
40%TPA(PDAF)$_3$@PS	0.09 ± 0.03	-5.27 ± 1.3	7.37×10^4	29.70	22.8

Programming conditions: V_G= -80 V for 20 ms. d) V_G= 80 V assist of light for 1 s.

^{1}H and ^{13}C-NMR spectra

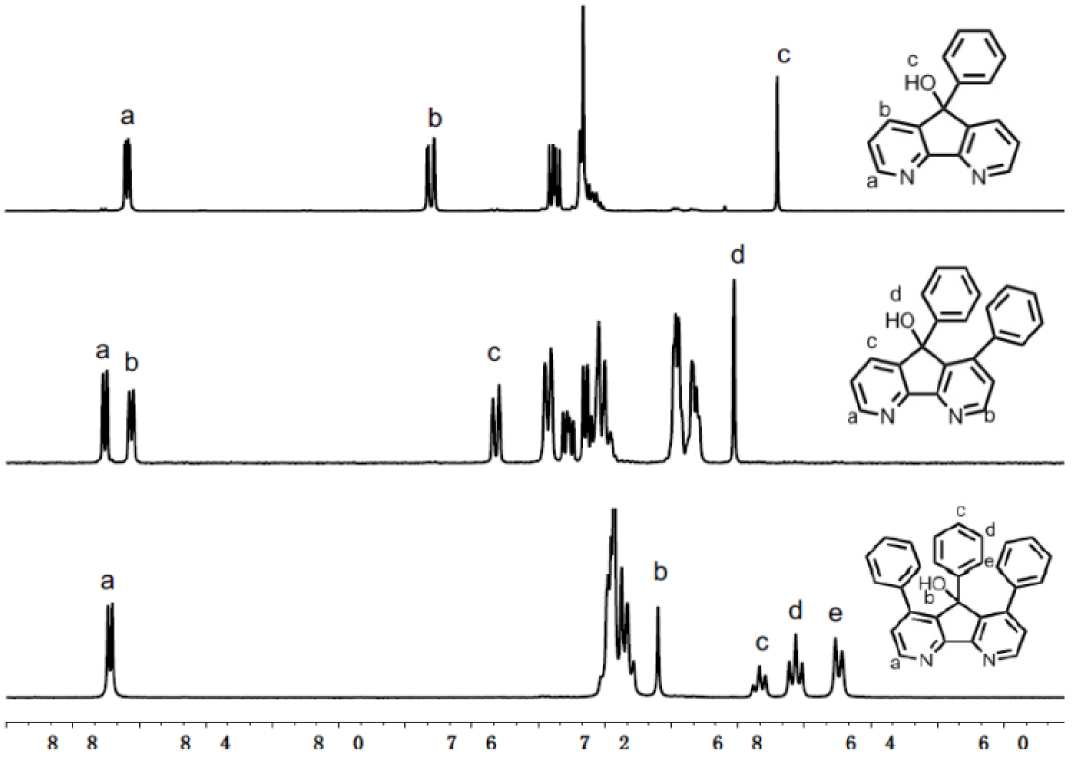

Figure. S20. Aromatic region of ^{1}H NMR spectra for nPDAFOH (n=1,2,3).

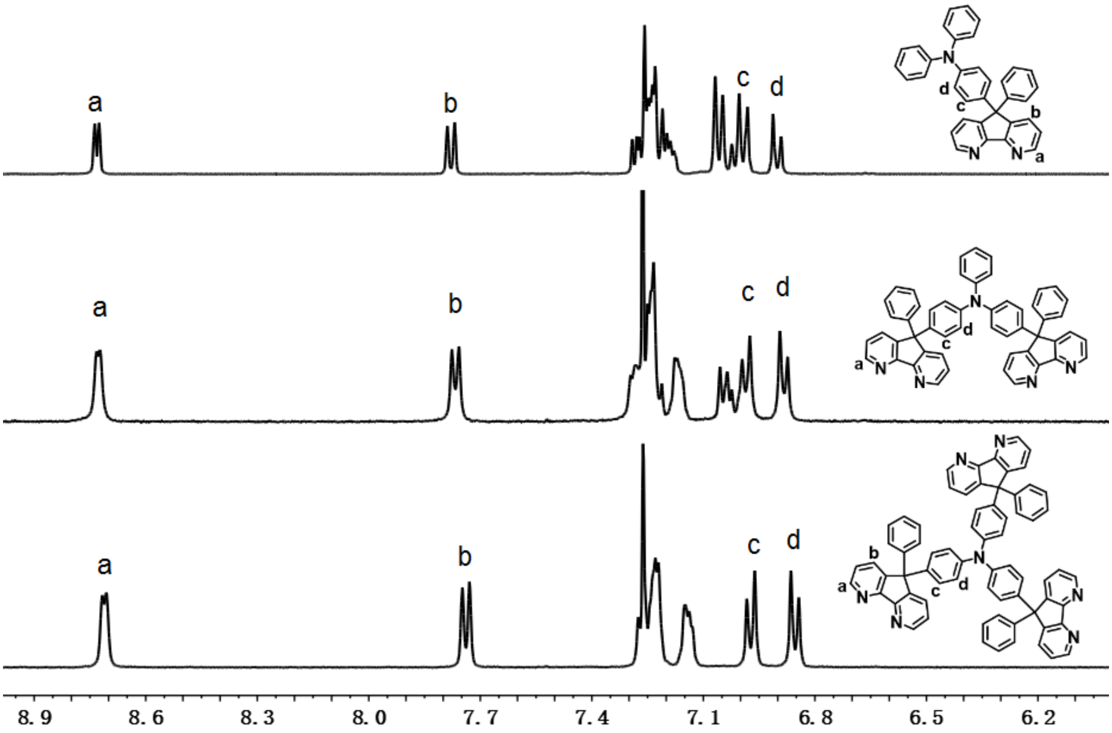

Figure. S21. Aromatic region of ^{1}H NMR spectra for TPA(PDAF)$_{n}$ (n=1,2,3).

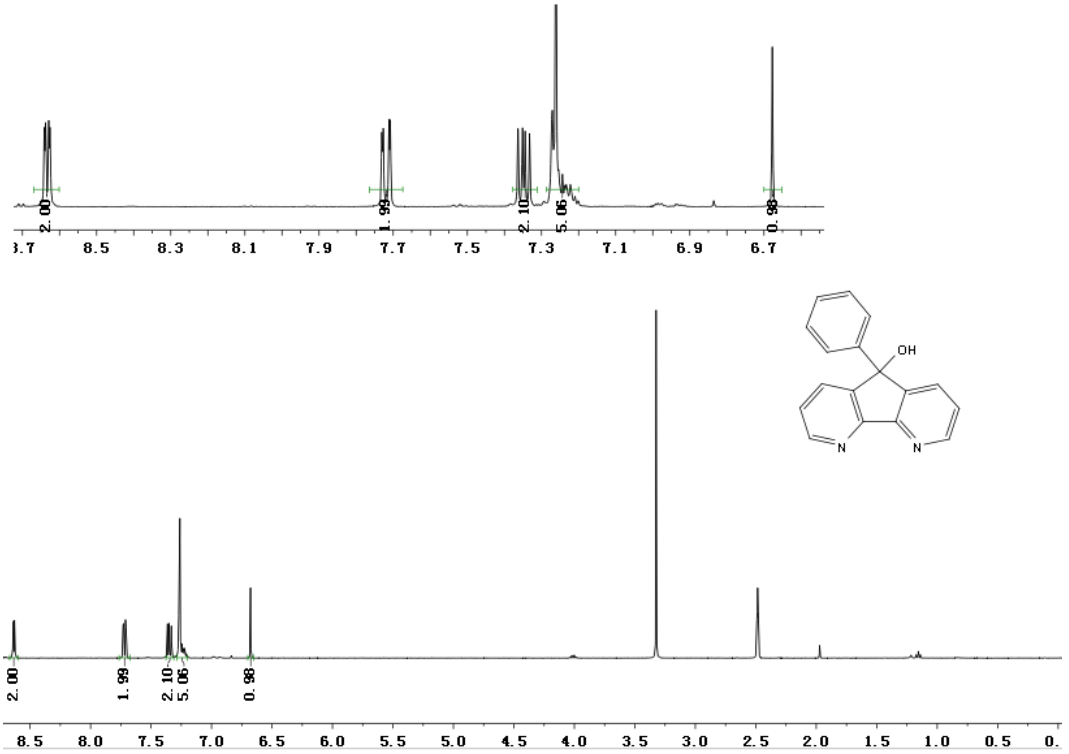

Figure. S22. ^{1}H NMR of **PDAFOH** in d_6-DMSO.

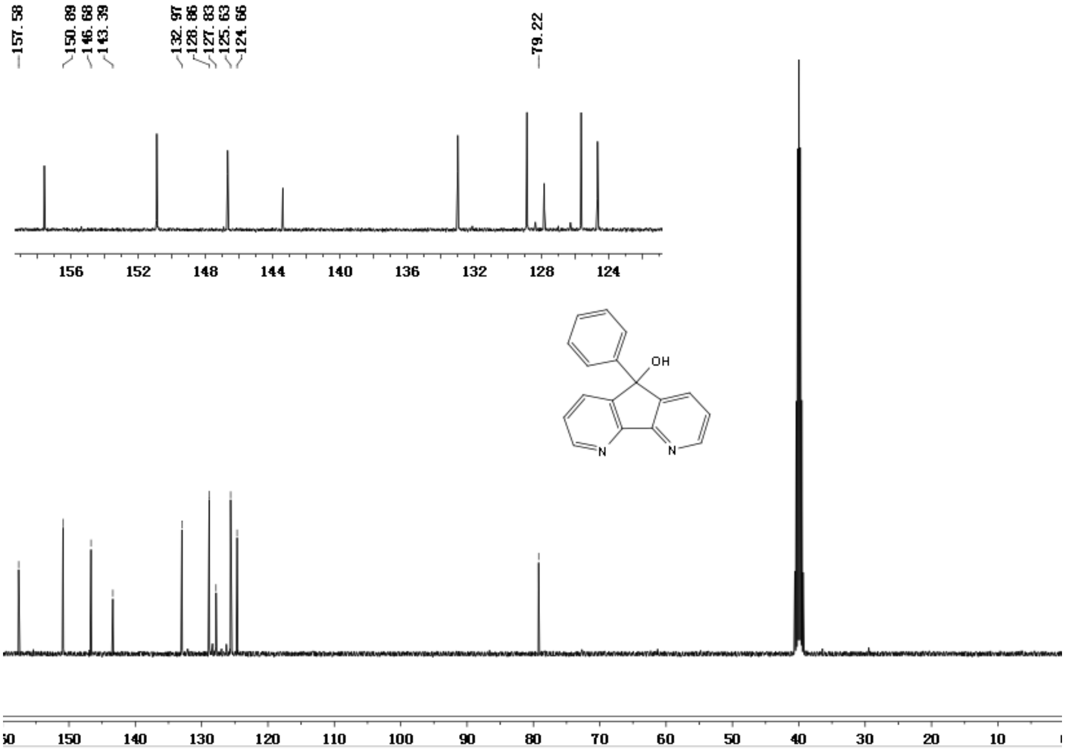

Figure. S23. ^{13}C NMR of **PDAFOH** in d_6-DMSO.

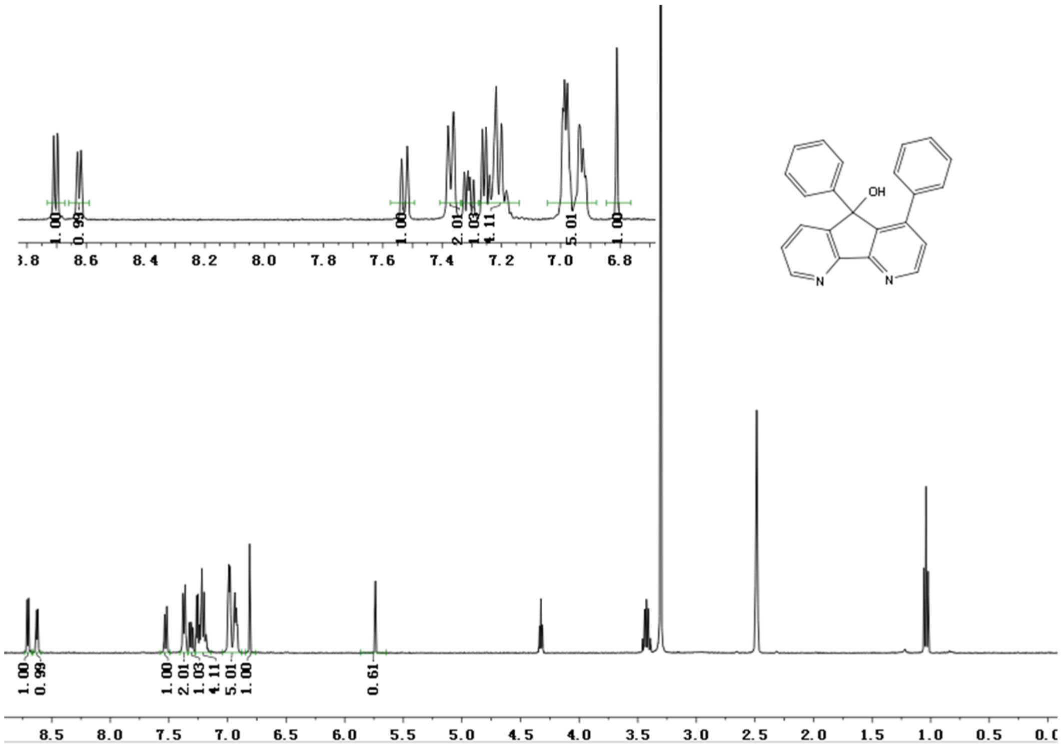

Figure. S24. ^{1}H NMR of **DPDAFOH** in d_6-DMSO.

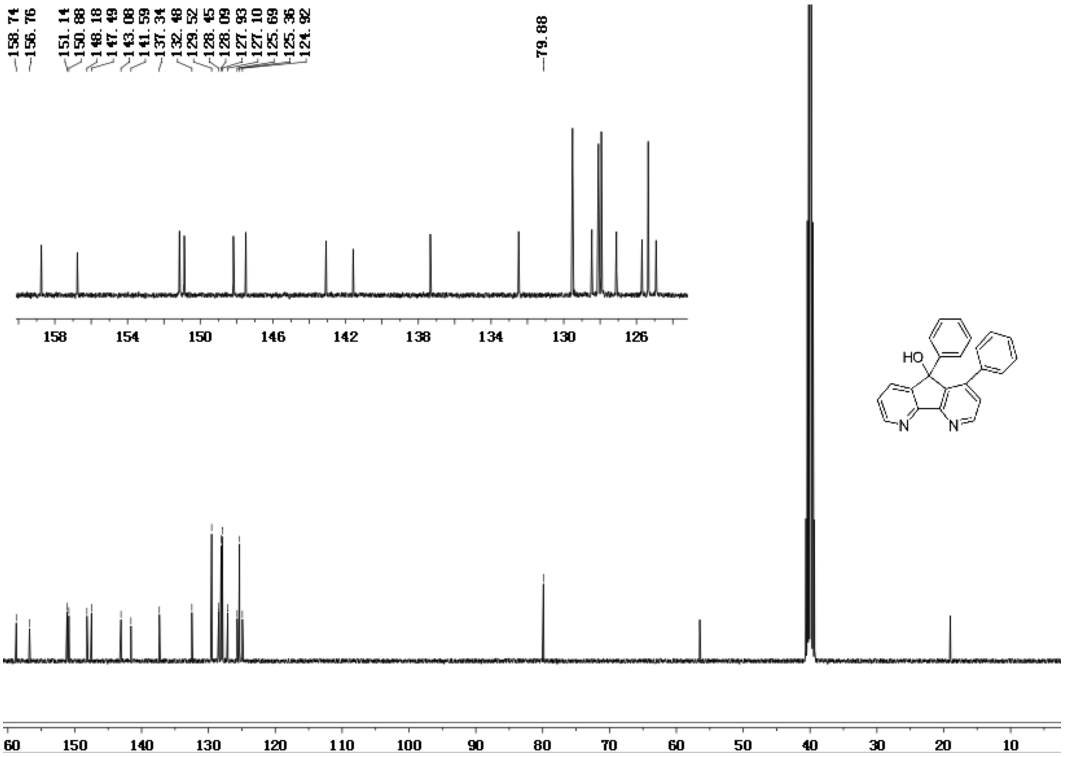

Figure. S25. ^{13}C NMR of **DPDAFOH** in d_6-DMSO.

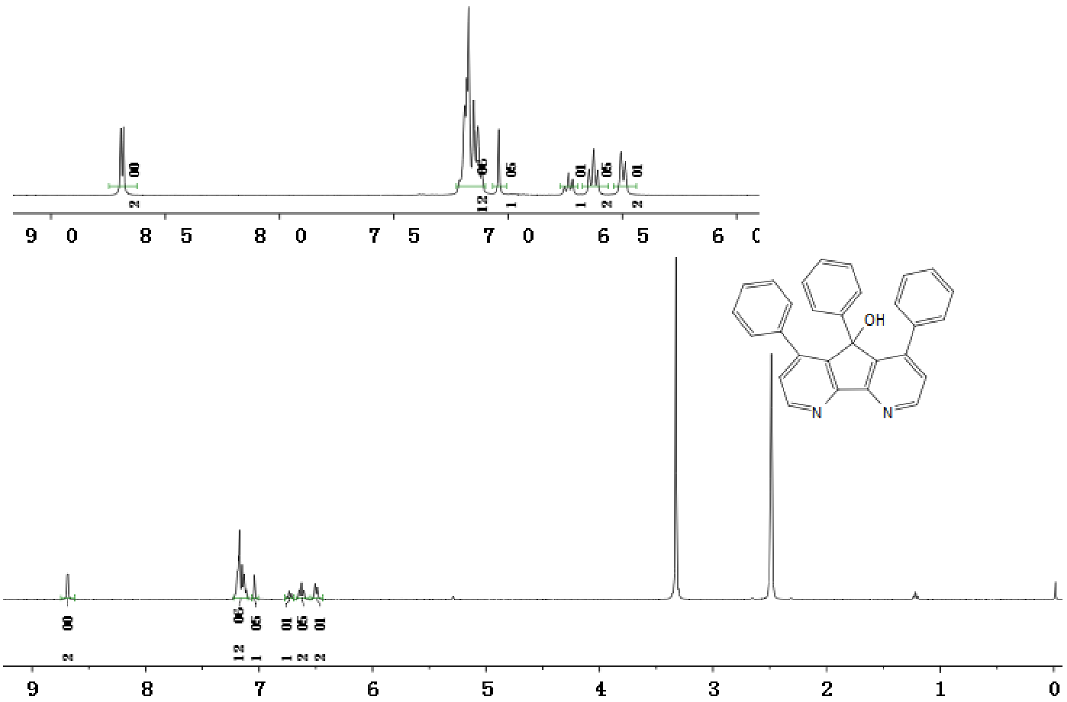

Figure. S26. ^{1}H NMR of **TPDAFOH** in d_6-DMSO.

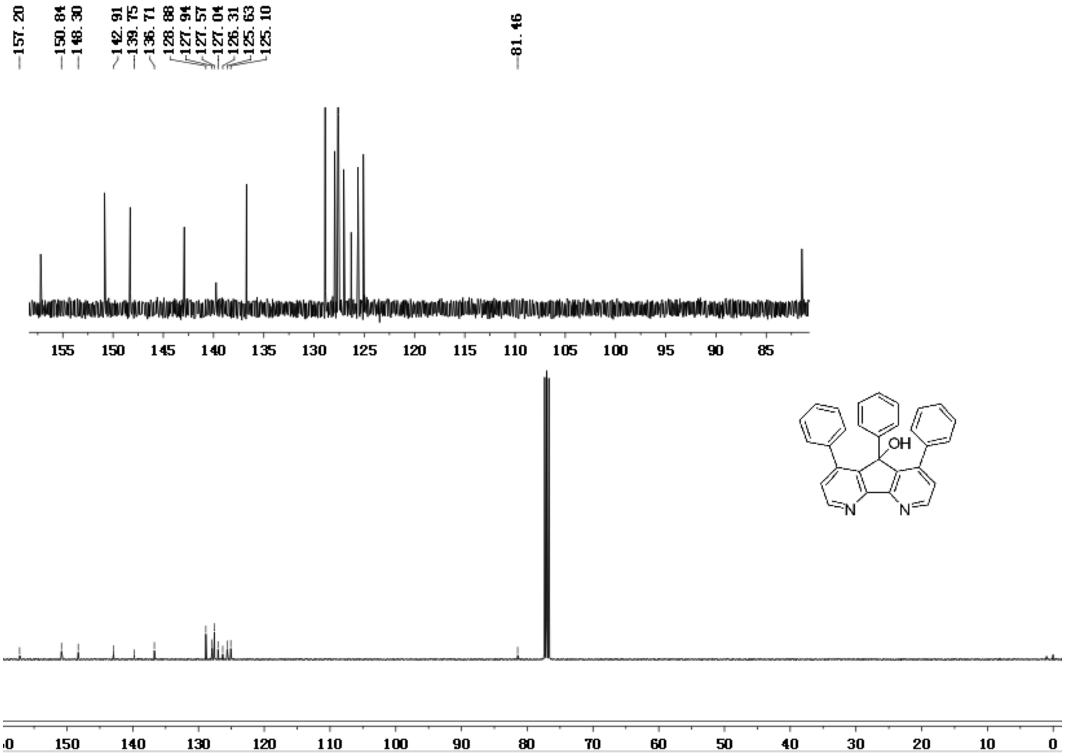

Figure. S27. ^{13}C NMR of **TPDAFOH** in $CDCl_3$.

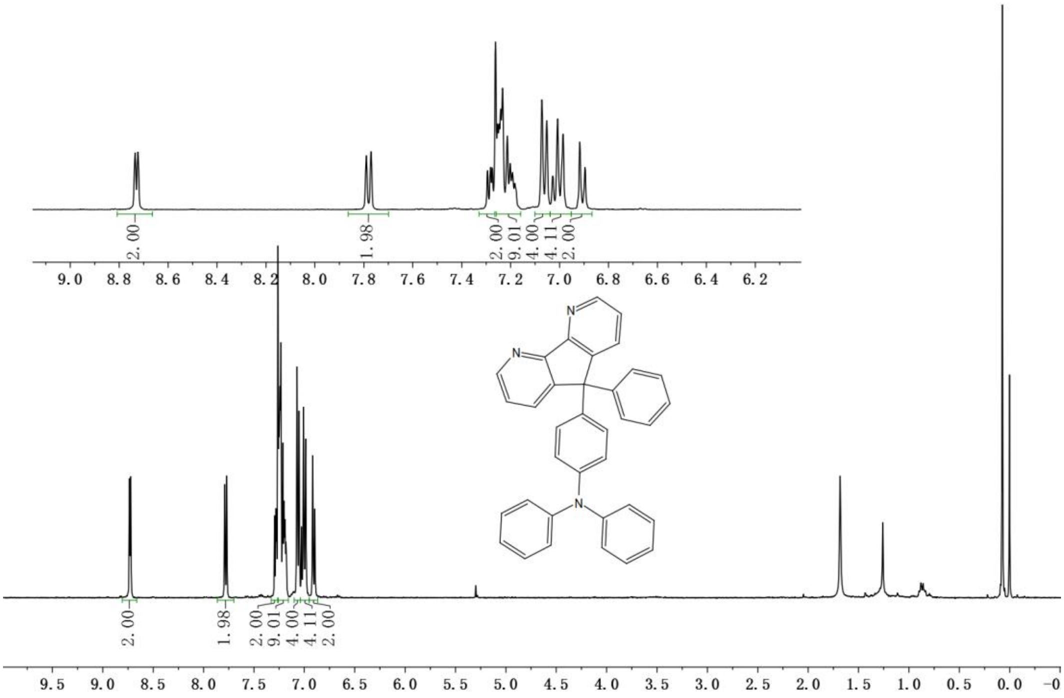

Fig. S28. ^{1}H NMR of **TPA(PDAF)$_1$** in $CDCl_3$.

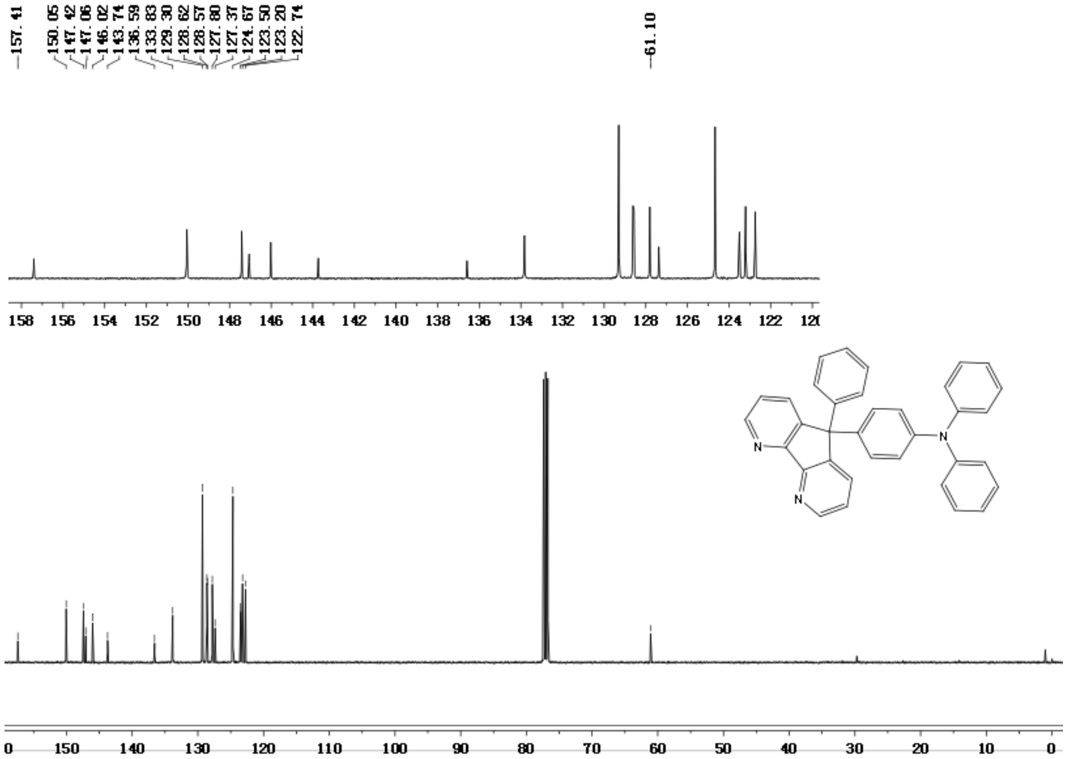

Fig. S29. ^{13}C NMR of **TPA(PDAF)$_1$** in $CDCl_3$.

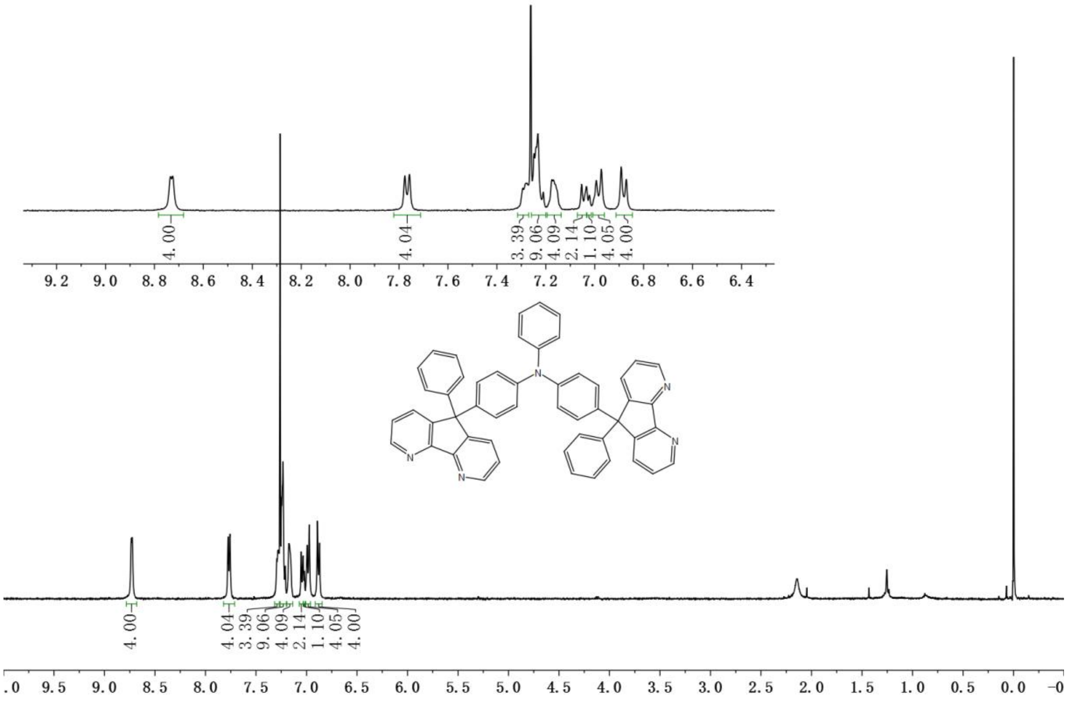

Fig. S30. ^{1}H NMR of **TPA(PDAF)$_2$** in $CDCl_3$.

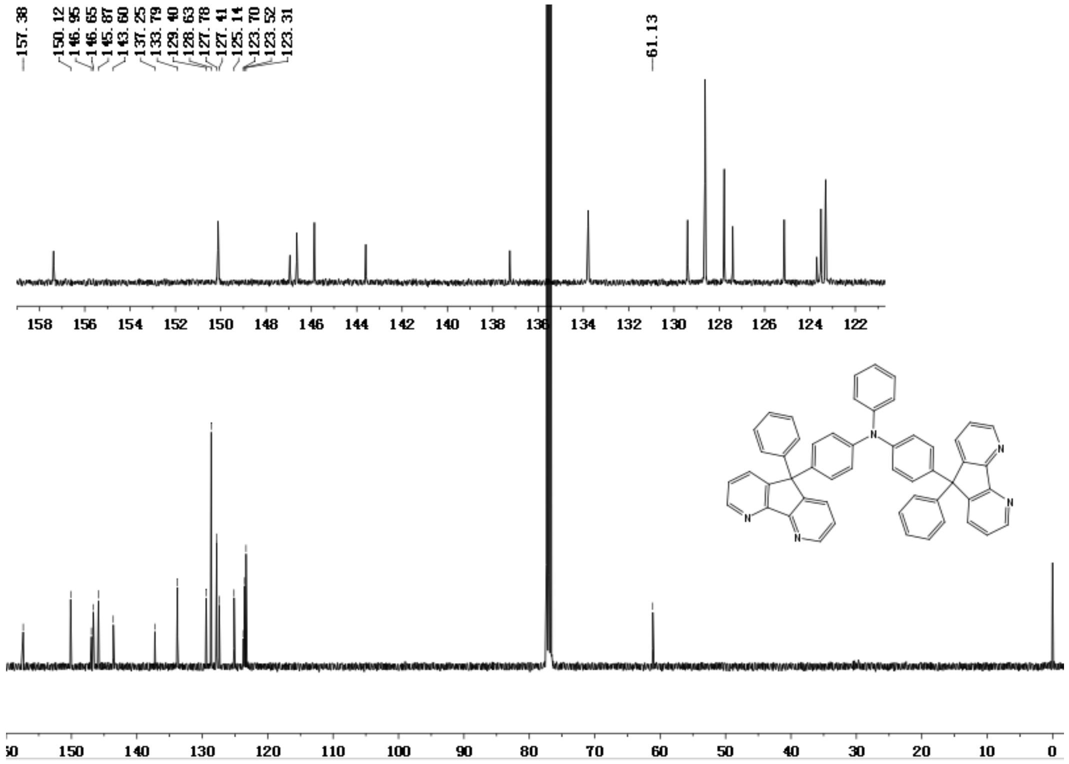

Fig. S31. ^{13}C NMR of **TPA(PDAF)$_2$** in $CDCl_3$.

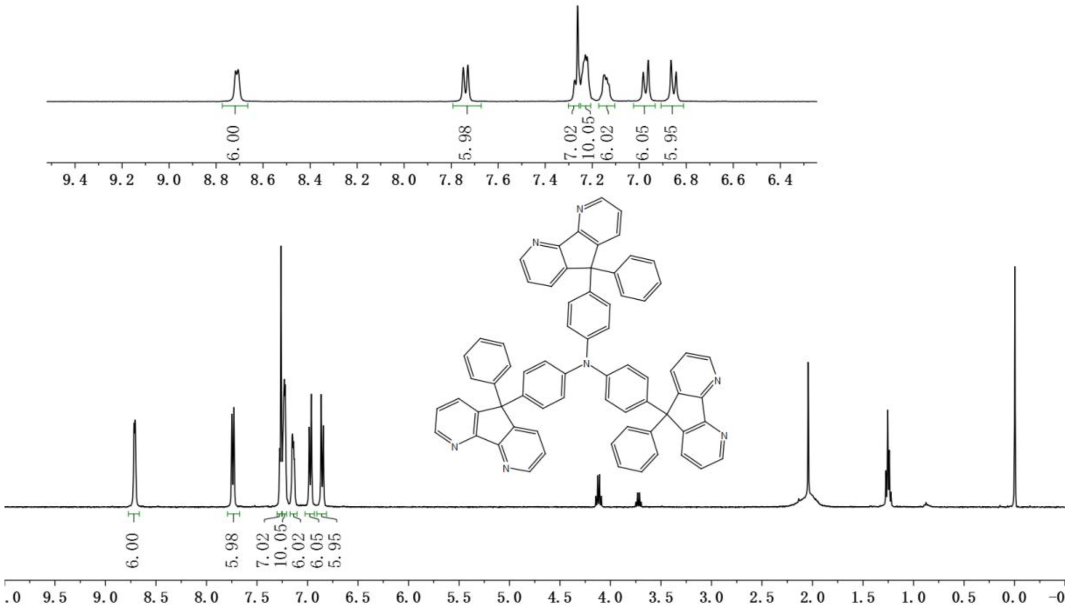

Fig. S32. ^{1}H NMR of **TPA(PDAF)$_3$** in $CDCl_3$.

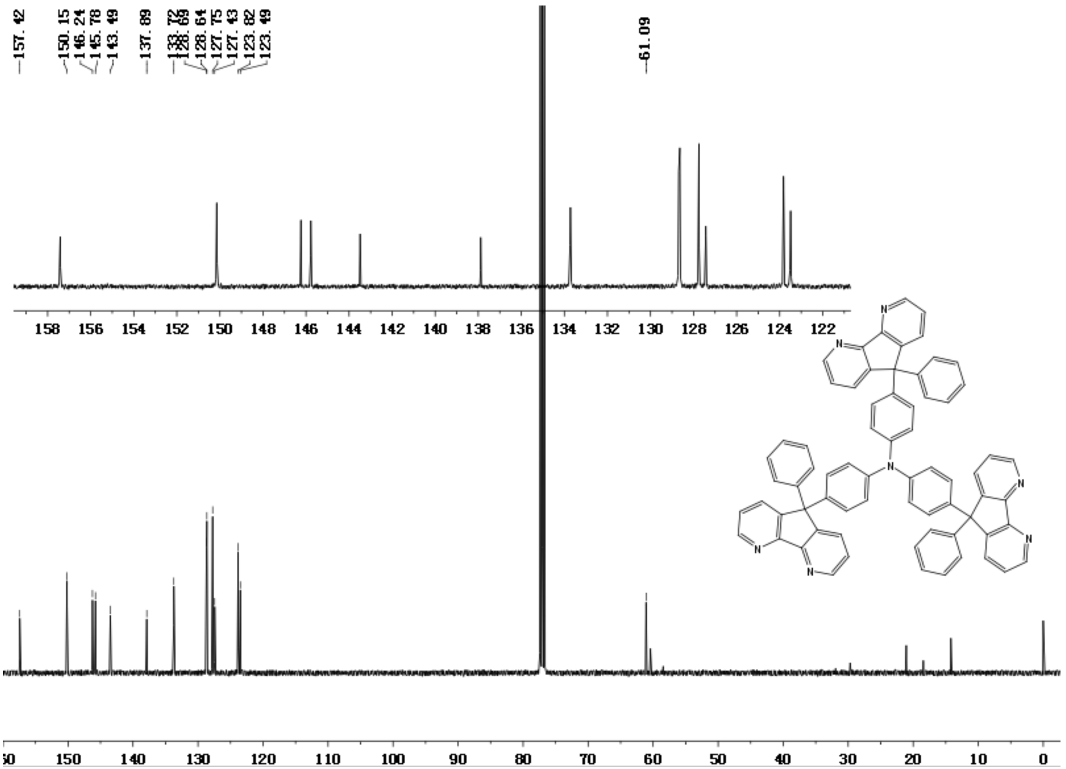

Fig. S33. ^{13}C NMR of **TPA(PDAF)$_3$** in $CDCl_3$.

Copyright WILEY-VCH Verlag GmbH & Co. KGaA, 69469 Weinheim, Germany, 2016.

Supporting Information

4,5-Diazafluorene-based Donor-Acceptor Small Molecules as Charge Trapping Elements for Tunable Nonvolatile Organic Transistor Memory

Yang Yu, Lin-Yi Bian, Jian-Guo Chen, Qi-Hao Ma, Yin-Xiang Li, Hai-Feng Ling, Quan-You Feng, Ling-Hai Xie*, Ming-Dong Yi, and Wei Huang*

General Methods. Unless otherwise information noted, all reagents were obtained from commercial source and used without purification further. Most of products were purified via column chromatography over silica gel (200-300 mesh) and some of them were purified by recrystallization. ^{1}H and ^{13}C NMR data were received from a Bruker 400 MHz NMR Fourier transform spectrometer (400 MHz and 100 MHz, respectively) at 20 ^{o}C. Chemical shifts are shown as δ in units of parts per million (ppm) relative to internal standard (^{1}H NMR: tetramethylsilane (TMS) = 0.00 ppm) or relative residual peaks (^{1}H NMR: 7.26 for $CDCl_3$, 2.50 for d_6-DMSO; ^{13}C NMR: 77.0 triplet for $CDCl_3$, 39.25 for d_6-DMSO). Multiplicities of every signal peak in briefly were shown as: s (singlet); d (doublet); t (triplet); q (quartet); dd (doublet of doublets); dt (doublet of triplets); m (multiplet). Coupling constants are expressed as a *J* value in Hz. HRMS were used as the determination of molecular weight.

X-ray crystallographic data. Crystallographic data for compound 3 and compound 6 were saved as the files of CCDC 974323.cif and 974342.cif, respectively. Data were collected at room temperature on a detector with graphitemonochromated Mo Ka radiation (λ=0.71073 Å). The absorption correction was applied by integration based on the crystal shape. Structures were solved by direct methods and refined against F2 with the full-matrix and least-squares methods. Hydrogen atoms were found by difference Fourier syntheses and were refined.

Crystal data for compound 3 (TPDAFOH). $C_{29}H_{20}O_1N_2$, M=412.49, T=298 K, monoclinic, C 2/c, a=22.3007(39) Å, b=6.7956(12) Å, c=28.9475(51) Å, β=101.973(2)°, V=4291.46(131) Å^3, ρ_{calcd} =1.27679 Mg m^{-3}, F(000)=1728, h, k, l_{max}=26, 8, 34, N_{ref}=3765, T_{min}, T_{max}=0.955, 0.975, $2\theta_{max}$= 50°, R(reflections)=0.0497(3302), R2(reflections)=0.2014(3754), S=1.493, N_{par}=291, Data completeness=0.997.

Crystal data for compound 6 (TPA(PDAF)$_3$). $C_{69}H_{45}N_7$, M=972.12, T=173(2) K, orthorhombic, Pna21(33) , Z=4, a=26.5050(16)Å, b=17.2404(8)^{o}, c=12.7703(8)^{o}, a=90.00^{o}, b=90.00^{o}, g=90.00^{o} Å, V=5835.48(58) Å^3, F(000)=2032, c_{lad}=1.107Mg m^{-3}, $2\theta_{max}$=50. 7°, R(reflections)=0.0723(5733), h, k, l_{max}=31, 20, 15, N_{ref}=10684[5602],

T_{min}, T_{max}= 0.955, 0.975, wR2(reflections)=0.2062(9834), S=1.043, N_{par}=686, Data completeness=1.76/0.92.

Crystal data for CCDC 974342 contained the supplementary crystallographic data for this paper. These data can be obtained free of charge from The Cambridge Crystallographic Data Centre via www.ccdc.cam.ac.uk/data_-request/cif.

Cyclic Voltammetry. CV were conducted at room temperature on the CHI660E system in a typical three-electrode cell with a platinum sheet working electrode, a platinum wire counter electrode, and a silver/silver nitrate (Ag/Ag^+) reference electrode. All electrochemical experiments were carried out under a nitrogen atmosphere at room temperature and performed on the solution of the sample on a glassy carbon electrode measured in Bu_4NPF_6 (0.1 M)/acetonitrile at a sweeping rate of 0.1V/s. According to the redox onset potentials of the CV measurements, the highest occupied molecular orbital (HOMO)/lowest unoccupied energy levels (LUMO) of the materials are estimated based on the reference energy level of ferrocene (4.8 eV below the vacuum). HOMO/ LUMO = - ($E_{ox/red}$ - 0.09 V) + 4.8 eV, where the value 0.09 V is the standard potential for ferrocene/ferrocenium vs Ag/Ag^+.

Theoretical calculations. The electronic ground states of all the compounds were computed by Becke’s three-parameter density functional in combination with Lee Yang Parr’s correlation functional (B3LYP) utilizing 6-31G(d) basis sets.27-28 The ground-state geometries of all the compounds were adequately optimized at the B3LYP/6-31G(d) level. TDDFT/B3LYP/6-31G(d) calculations of the excitation energies were then performed at these optimized geometries. All computations were performed using the Gaussian 09 package.

General synthesis of six compounds

Synthetic procedures of 9-phenyl-4,5-diazafluorene-9-ol (compound 1),
1,9-diphenyl-4,5 -diazafluorene-9-ol (compound 2) and
1,8,9-triphenyl-4,5-diazafluorene-9-ol (compound 3) by Grignard reaction.

The synthetization of phenyl magnesium bromide solution: Magnesium turnings and a piece of iodine were added into a 100 ml flask under a N_2 atmosphere. Then, 2-3 drops of bromobenzene and 5 ml tetrahydrofuran (THF) were added into the flask. The reaction was triggered by heated to a suitable temperature, meanwhile the color of reaction liquid turned from orange to transparent. Then remained bromobenzene was added into the flask with some other THF in ice-bath. Ultimate, phenyl magnesium bromide solution can be obtain after 2 h of reflux in oil-bath (55 ^oC).

4,5-diazafluorene-9-one (DAFO), which was synthesized as the method of precious work of our group,[22] was added into 500 ml flask under a N_2 atmosphere. Then 200 ml tetrahydrofuran (THF) was injected into the flask to dissolve the DAFO in Acetone dry ice bath (-78 ^oC) for compound 1. Synthesis methods of compound 2 and compound 3 were the same as compound 1 except the reaction temperature (-10 ^oC for compound 2 and 40 ^oC for compound 3). Phenyl magnesium bromide solution was injected into the flask containing DAFO after extracted from its flask by injection

syringe. The reaction can be quenched by Saturated ammonium chloride solution after stirring for 15min. the reaction mixture was extracted with CH_2Cl_2 and dried with $MgSO_4$. The obtained organic phase was condensed by rotary evaporation under reduced pressure and the crude residue was subjected to flash column chromatography with ethyl acetate (EA) and petroleum ether to obtain the target products.

Compound 1: yield 91%. White solid. ^{1}H NMR (400 MHz, d$_6$-DMSO): δ (ppm) 8.63 – 8.64(d, J = 6.4 Hz, 2H), 7.73 – 7.71 (d, J = 9.2 Hz 2H), 7.36 – 7.33 (m, J = 12.8 Hz 2H), 7.27 – 7.23 (m, J = 24.4 Hz, 5H), 6.68 (s, 1H). ^{13}C NMR (100 MHz, d$_6$-DMSO): δ (ppm) 157.58, 150.89, 146.68, 143.39, 132.97, 128.86, 127.83, 125.63, 124.66, 79.22. HRMS: m/z calcd for [M+H$^+$] $C_{17}H_{13}O_1N_2$: 261.1022; found: 261.1021.

Compound 2: yield 53%. White solid. ^{1}H NMR (400 MHz, d$_6$-DMSO): δ (ppm) 8.71 – 8.70 (d, J = 5.2 Hz,1H), 8.63 – 8.62 (d, J = 4.8 Hz, 1H), 7.53 – 7.51 (d, J = 7.6Hz, 1H), 7.37 – 7.35 (d, J = 7.2, 2H), 7.32 – 7.31 (m, J = 7.2, 1H), 7.28 – 7.27 (d, J = 12 Hz, 1H), 7.20 – 7.19 (d, J = 4.8 Hz, 1H), 6.98 – 6.97 (m, J =5.6 Hz, 3H), 6.93 – 6.92 (d, J = 4.8 Hz 2H), 6.81 (s, 1H). ^{13}C NMR (100 MHz, (d$_6$-DMSO): δ (ppm) 158.74, 156.76, 151.14, 150.88, 148.18, 147.49, 143.08, 141.59, 137.34, 132.48, 129.52, 128.45, 128.09, 127.93, 127.10, 125.69, 125.36, 124.92, 79.88. HRMS: m/z calcd for [M+H$^+$] $C_{23}H_{16}N_2O$: 336.1272; found: 336.1270.

Compound 3: yield 77%. White solid. ^{1}H NMR (400 MHz, d$_6$-DMSO): δ (ppm) 8.73 – 8.72 (d, J = 5.2 Hz, 2H), 7.23 – 7.08 (m, 12H), 7.05 (s, 1H), 6.75-6.72 (dd, 1H), 6.64 – 6.62(dd, 2H), 6.51 – 6.49 (d, J = 4.8 Hz, 2H). ^{13}C NMR (100 MHz, $CDCl_3$): δ (ppm) 157.20, 150.84, 148.30, 142.91, 139.75, 136.71, 128.88, 127.94, 127.57, 127.04, 126.31, 125.63, 125.10, 81.46. HRMS: m/z calcd for [M+H$^+$] $C_{29}H_{20}N_2O$: 412.1576; found: 412.1579.

General procedure for Friedel–Crafts reaction

9-phenyl-4,5-diazafluorene-9-ol and triphenylamine (TPA) were added into a 500 mL flask and stirred to dissolve in dichloromethane (DCM) in room temperature. Then, sulfuric acid was added as a catalyst into the solution and refluxed for 6 h. Then neutralized the solution with NaOH (aq) to pH at about 8. After stirring for another 1 h to neutralize adequately, the reaction mixture was extracted with CH_2Cl_2 and dried with $MgSO_4$. The obtained organic phase was condensed by rotary evaporation under reduced pressure and the crude residue was subjected to flash column chromatography with ethyl acetate (EA) and petroleum ether to obtain the target product, which was offwhite solid.

Synthesis of *N*,*N*-diphenyl-4-(9-phenyl-4,5-diazafluorene-9-yl)phenylamine (**TPA(PDAF)$_1$**) (compound 4)

Following the above procedures, 9-phenly-4,5-diazafluoren-9-ol (0.26 g, 1 mmol) and triphenylamine (0.74 g, 3 mmol) were added into a 250 mL flask and stired to dissolve in 150 mL DCM. Then, 2.5 mL sulfuric acid as catalyst was added into the solution and refluxed for 6 h. Then neutralized the solution with NaOH (aq) to pH at

about 8. After stirring for another 1 h to neutralize adequately, the reaction mixture was extracted with CH_2Cl_2 and dried with $MgSO_4$. The obtained organic phase was condensed by rotary evaporation under reduced pressure and the crude residue was subjected to flash column chromatography with ethyl acetate (EA) and petroleum ether to obtain the target product of **TPA(PDAF)$_1$**. Offwhite solid. 273 mg. 0.56 mmol. Yield: 56%. ^{1}H NMR (400 MHz, $CDCl_3$): δ (ppm) 8.73 – 8.72 (dd, *J* = 4.7 Hz, 1.2 Hz, 2H), 7.79 – 7.77 (dd, *J* = 7.8 Hz, 1.3 Hz, 2H), 7.29 – 7.27 (dd, *J* = 7.6 Hz, 4.8 Hz, 2H), 7.25 – 7.18 (m, 9H), 7.07 – 7.05 (d, *J* = 7.6 Hz, 4H), 7.03 – 6.98 (m, 4H), 6.92 – 6.90 (d, *J* = 8.7 Hz, 2H). ^{13}C NMR (100 MHz, $CDCl_3$): δ (ppm) 157.4, 150.1, 147.4, 147.1, 146.0, 143.7, 136.6, 133.8, 129.3, 128.6, 127.8, 127.4, 124.7, 123.5, 123.2, 122.7, 61.1. HRMS: m/z calcd for [M+H$^+$] $C_{35}H_{26}N_3$: 488.2121; found: 488.2120.

Synthesis of *N*,*N*-bis(4-(9-phenyl-4,5-diazafluorene-9-yl)phenyl)phenylamine (**TPA(PDAF)$_2$**) (compound 5) and tris(4-(9-phenyl-diazafluorene-9-yl)phenyl)amine (**TPA(PDAF)$_3$**) (compound 6)

According to the general procedure, the two products were obtained by reacting 9-phenly-4,5-diazafluoren-9-ol (0.52 g, 2 mmol) with triphenylamine (0.245 g, 1 mmol) and 9-phenly-4,5-diazafluoren-9-ol (0.94 g, 3.6 mmol) with triphenylamine (0.245 g, 1 mmol), flashing column chromatography with eluent ethyl acetate and petroleum ether; yield 46 % and 75 % of the products TPA(PDAF)$_2$ and TPA(PDAF)$_3$, respectively.

TPA(PDAF)$_2$: Offwhite solid. 336 mg. 0.46 mmol. Yield: 46%. ^{1}H NMR (400 MHz, $CDCl_3$): δ (ppm) 8.72 – 8.71 (d, *J* = 4.7 Hz, 4H), 7.77 – 7.74 (dd, *J* = 7.5 Hz, 0.7 Hz, 4H), 7.28 – 7.21 (m, 13H), 7.17 – 7.15 (m, 4H), 7.05 – 7.03 (d, *J* = 7.6 Hz, 2H), 7.02 – 7.00 (d, *J* = 7.6 Hz, 2H), 6.99 – 6.97 (d, *J* = 8.8 Hz, 2H), 6.89 – 6.97 (d, *J* = 8.7 Hz, 4H). ^{13}C NMR (100 MHz, $CDCl_3$): δ (ppm) 157.4, 150.1, 146.9, 146.7, 145.9, 143.6, 137.2, 133.8, 129.4, 128.6, 127.8, 127.4, 125.1, 123.7, 123.5, 123.3, 61.1. HRMS: m/z calcd for [M+H$^+$] $C_{52}H_{36}N_5$: 730.2965; found: 730.2960.

TPA(PDAF)$_3$: Offwhite solid. 729 mg. 0.75 mmol. Yield: 75%. ^{1}H NMR (400 MHz, $CDCl_3$): δ (ppm) 8.71 – 8.69 (dd, *J* = 4.8 Hz, 1.2 Hz, 6H), 7.74 – 7.72 (dd, *J* = 7.8 Hz, 1.2 Hz, 6H), 7.27 – 7.25 (dd, *J* = 7.6 Hz, 4.8 Hz, 6H), 7.24 – 7.22 (m, 9H), 7.15 – 7.13 (m, 6H), 6.98 – 6.96 (d, *J* = 8.7 Hz, 6H), 6.86 – 6.84 (d, *J* = 8.7 Hz, 6H). ^{13}C NMR (100 MHz, $CDCl_3$): δ (ppm) 157.4, 150.1, 146.2, 145.8, 143.5, 137.9, 133.7, 128.7, 128.6, 127.8, 127.4, 123.8, 123.5, 61.1. HRMS: m/z calcd for [M+H$^+$] $C_{69}H_{46}N_7$: 972.3809; found: 972.3805.

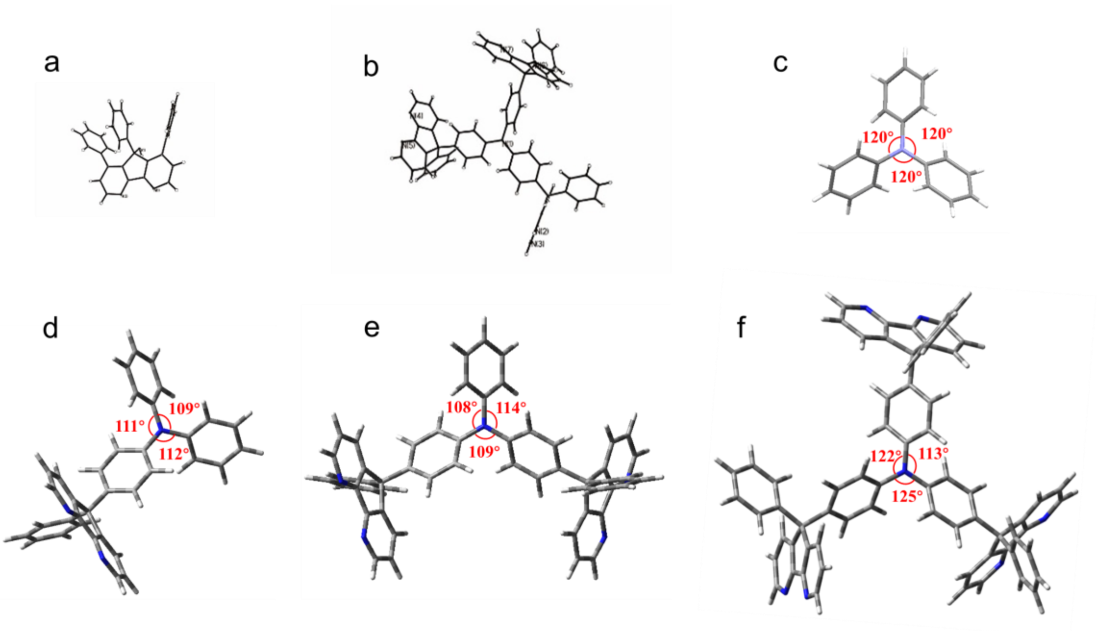

Figure. S1. X-ray single crystal structures of (a) TPDAFOH and (b) $TPA(PDAF)_3$, Angles of (c) TPA, (d) $TPA(PDAF)_1$, (e) $TPA(PDAF)_2$ and (f) $TPA(PDAF)_3$.The single crystal structure of TPA can be referred to Y. X. Li, S. S. Wang, Y. Yu, H. Zhang, W. Y. Wang, R. Q. Yang, L. H. Xie, F. Liu, Z. Q. Lin, N. E. Shi, L. T. Sun, W. Huang, *Small*. **2018**, 14.

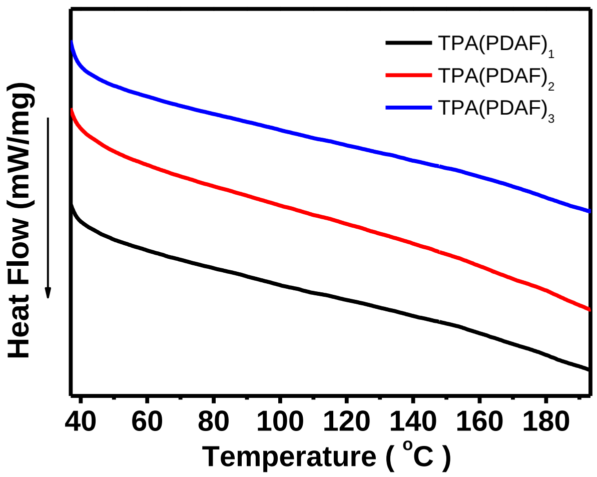

Figure. S2. DSC curve of $TPA(PDAF)_n$ (n=1,2,3) with the heating rate of 10 °C/min in the nitrogen atmosphere.

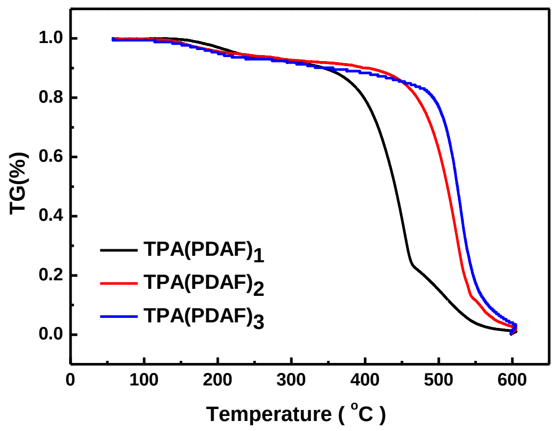

Figure. S3. TGA curve of $TPA(PDAF)_n$ (n=1,2,3) in nitrogen atmosphere.

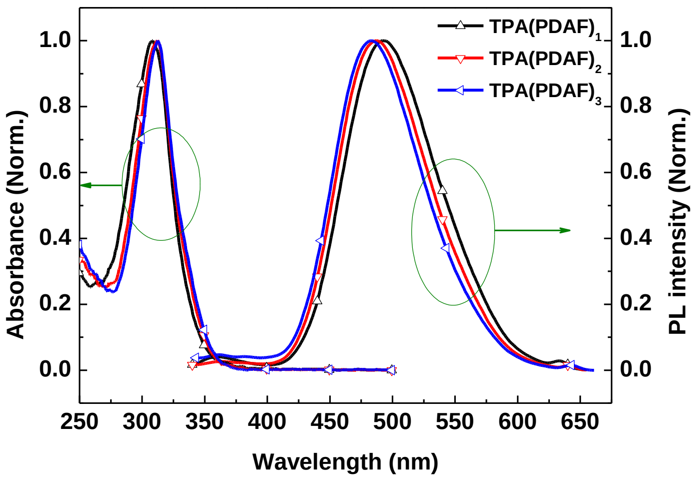

Figure. S4. The absorption and emission spectra of $TPA(PDAF)_n$ (n=1,2,3) in chloroform solutions.

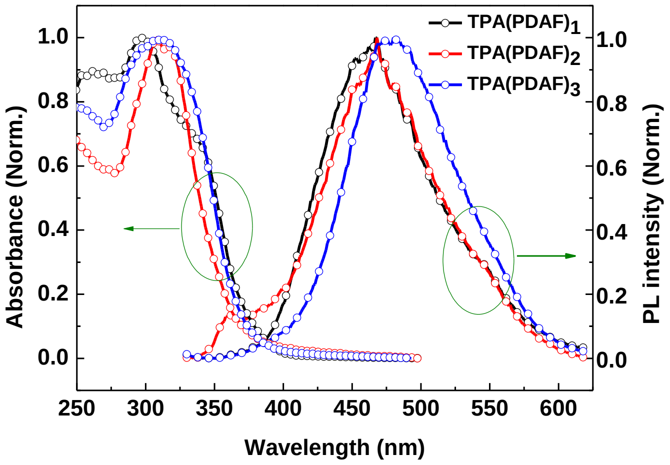

Figure. S5. The absorption and emission spectra of $TPA(PDAF)_n$ (n=1,2,3) in films.

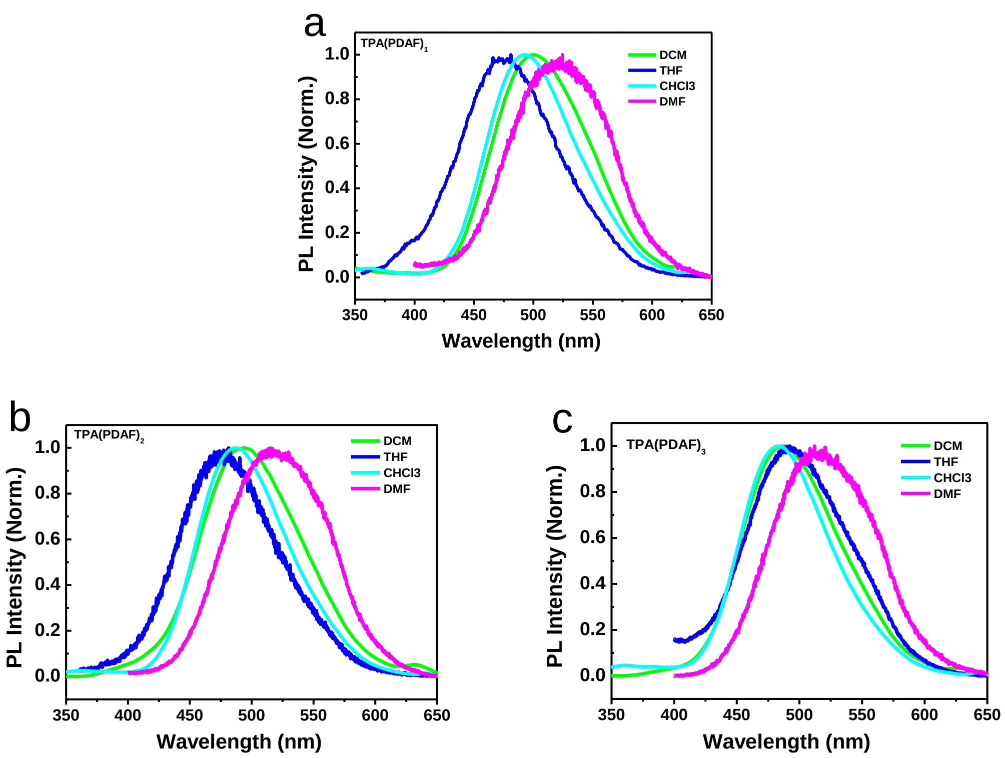

Figure. S6. The PL spectra of the (a) $TPA(PDAF)_1$, (b) $TPA(PDAF)_2$, and (c) $TPA(PDAF)_3$ in different solutions (DCM , $CHCl_3$, THF and DMF).

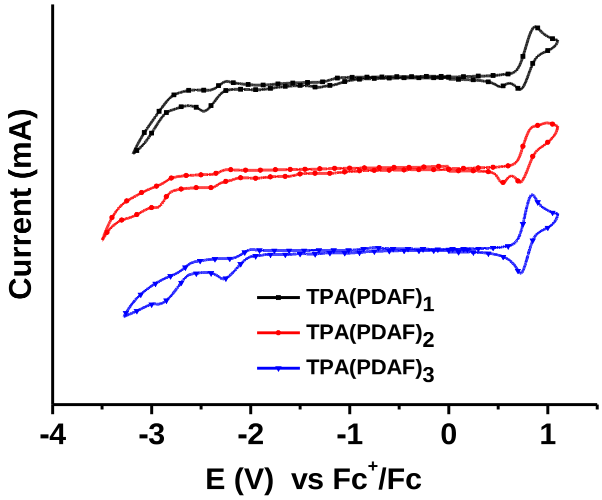

Figure. S7. Cyclic voltammograms of $TPA(PDAF)_n$ (n=1,2,3) in CH_2Cl_2 solution (forward direction) and in THF solution (negative direction). Cyclic voltammogram of the three compounds measured with a scan rate of 100 mV s^{-1} .

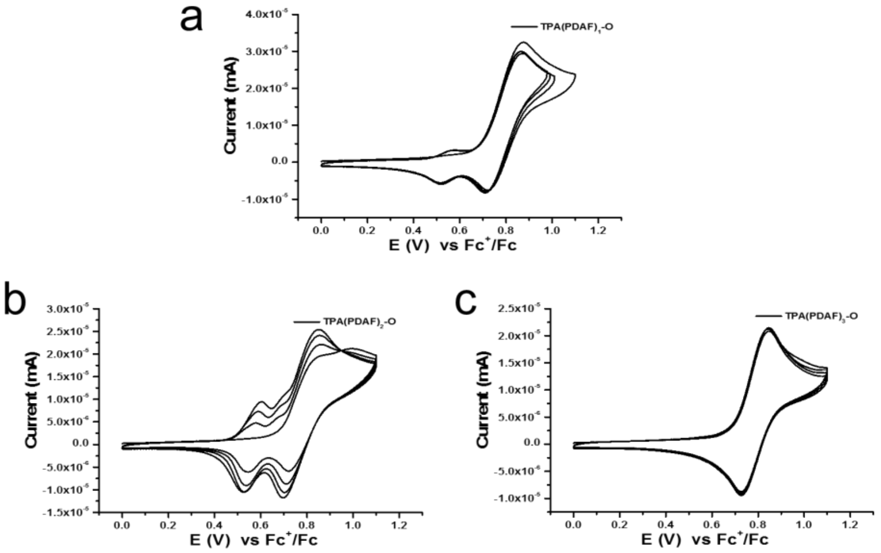

Figure. S8. Cyclic voltammograms of (a) TPA(PDAF)$_1$, (b) TPA(PDAF)$_2$, (c) TPA(PDAF)$_3$ with successive scan curves in the anodic scan.

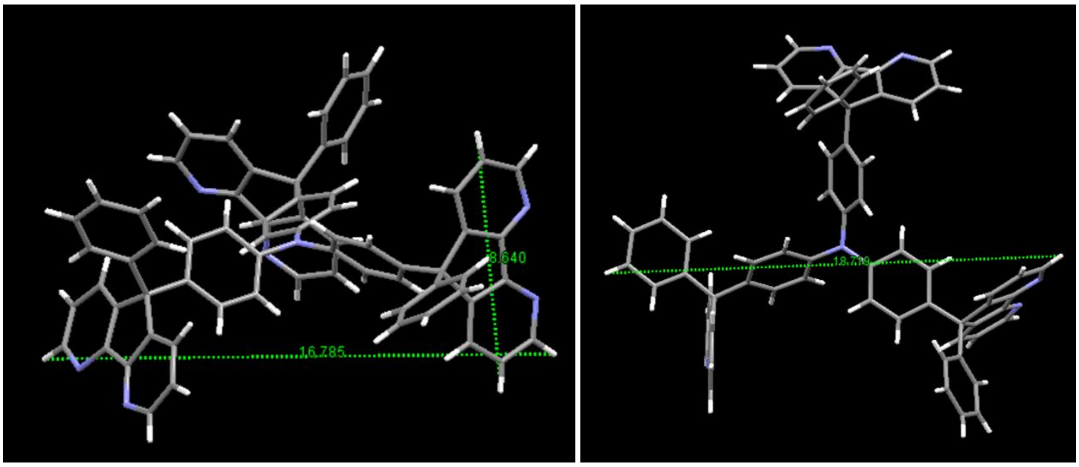

Figure. S9. The 3-dimensional diameter of TPA(PDAF)$_3$ based on the single crystal structure.

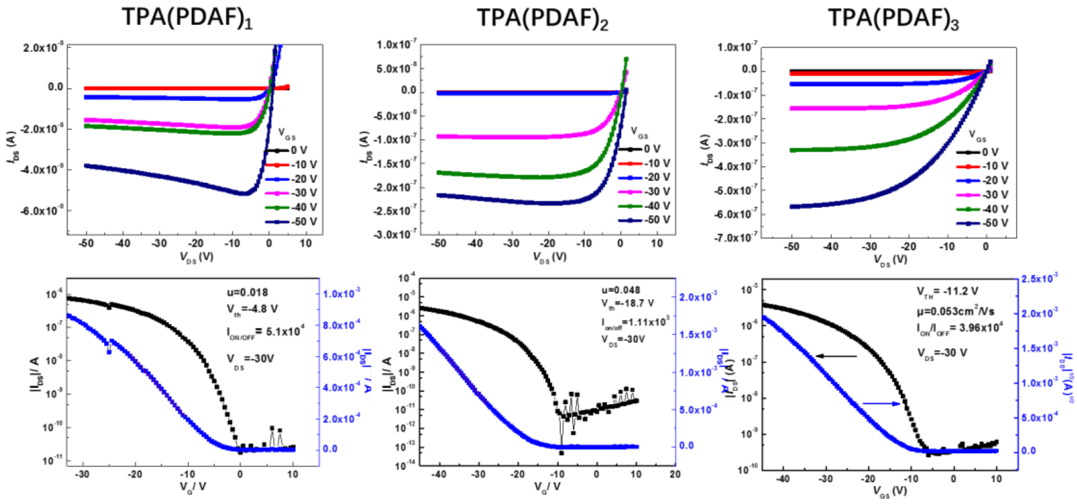

Figure. S10. The output and transfer characteristics of the devices with $TPA(PDAF)_n$ (n=1,2,3) as charge storage layers.

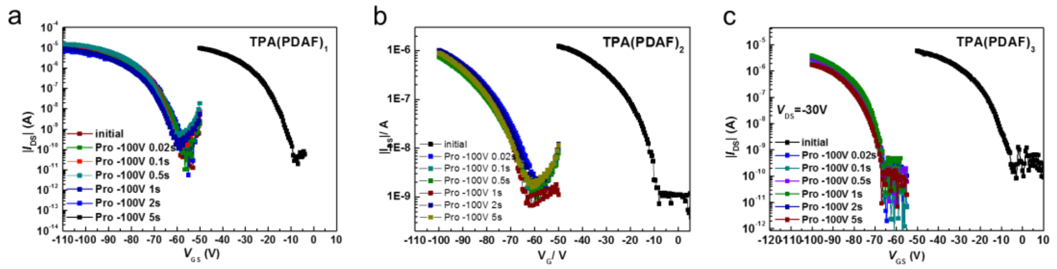

Figure. S11. The transfer characteristics of (a)$TPA(PDAF)_1$, (b)$TPA(PDAF)_2$ and (c)$TPA(PDAF)_3$ based OFET memory. The programming time was changing from 20 ms to 5 s under -100 V gate voltage.

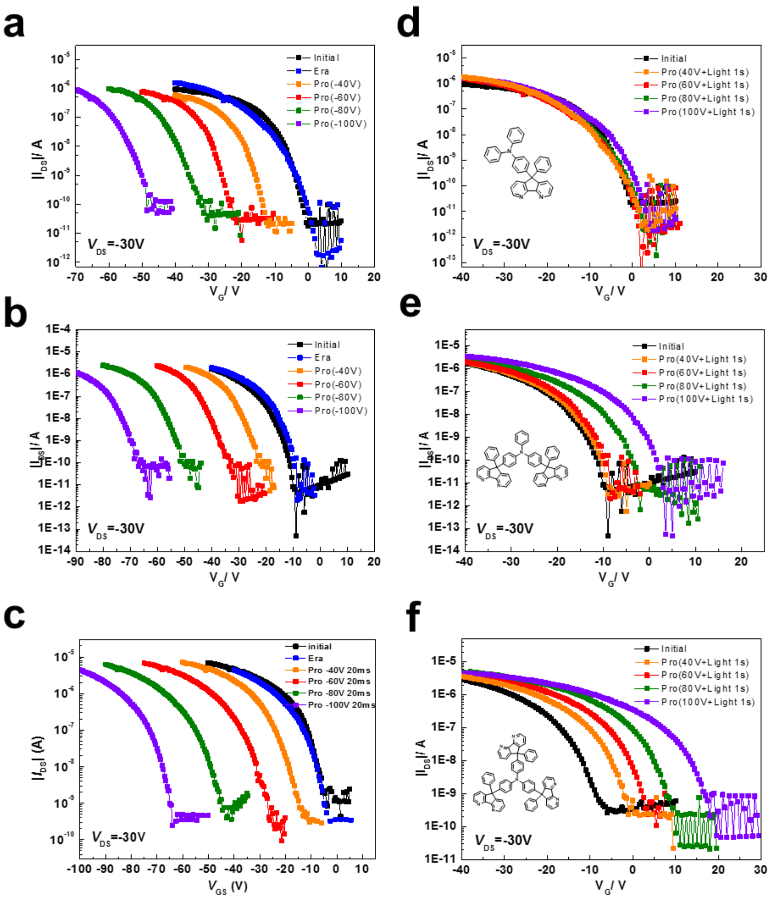

Figure. S12. The transfer curves of the (a) TPA(PDAF)$_1$, (b) TPA(PDAF)$_2$, (c) TPA(PDAF)$_3$ based devices for the programming processes under negative gate voltages ranging from -40, -60, -80 to -100 V. The drain current was measured at V_D = -30 V and the programming time was 20 ms. And (d) TPA(PDAF)$_1$, (e) TPA(PDAF)$_2$, (f) TPA(PDAF)$_3$ based devices for the positive programming processes upon positive gate voltages ranging from 40, 60, 80 to 100 V, with assist of light for 1 s.

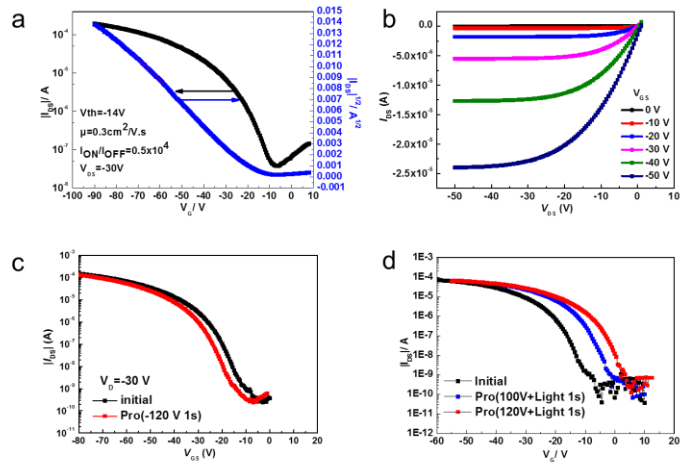

Figure S13. The electrical characteristics of the devices using only diazafluorene as charge trapping layers.

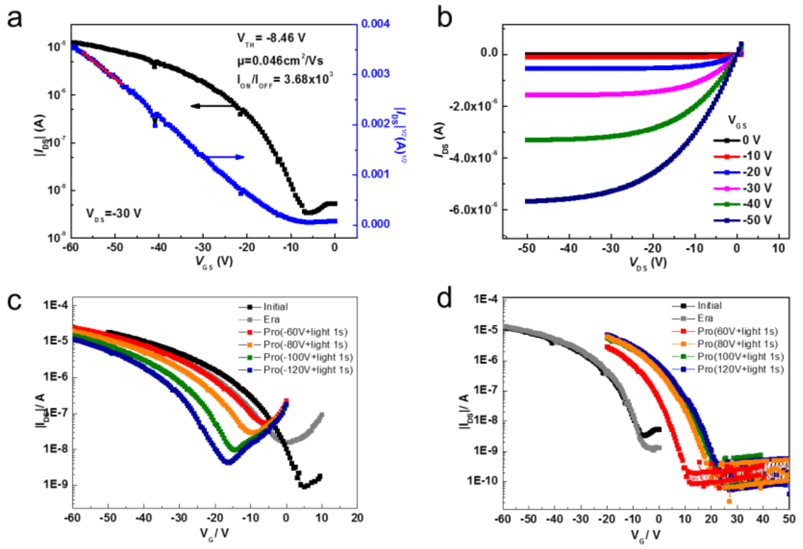

Figure S14. The electrical characteristics of the devices using only triphenylamine as charge trapping layers.

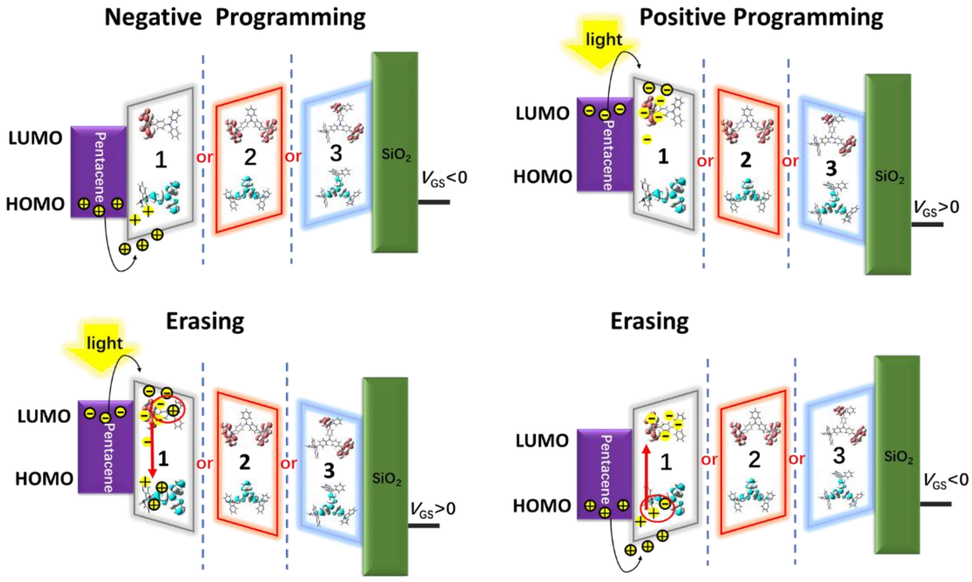

Figure. S15. Energy band diagrams of pentacene and TPA(PDAF)$_n$ under negative and positive gate voltages. (a) hole trapping (PGM mode) (b) electron trapping (PGM mode), (c) electron detrapping and recombination with hole (ERS mode) and (d) hole detrapping and recombination with electron (ERS mode)

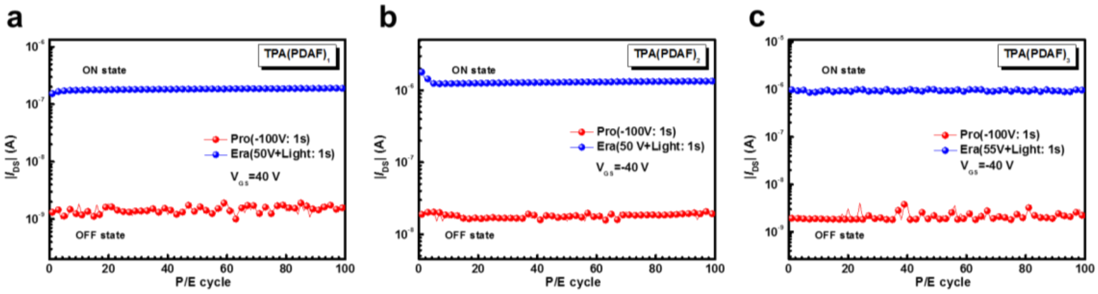

Figure. S16. Write-read-erase-read cycles testing of (a) $TPA(PDAF)_1$, (b) $TPA(PDAF)_2$ and (c) $TPA(PDAF)_3$ based devices.

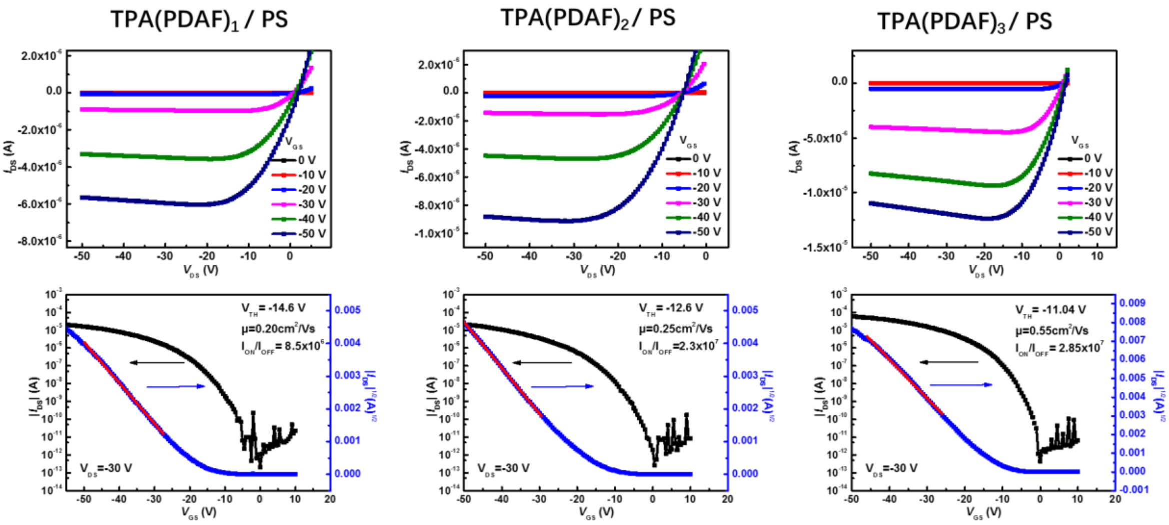

Figure. S17. The output and transfer characteristics of the devices with $TPA(PDAF)_n$ (n=1,2,3) blend with PS as charge storage layers.

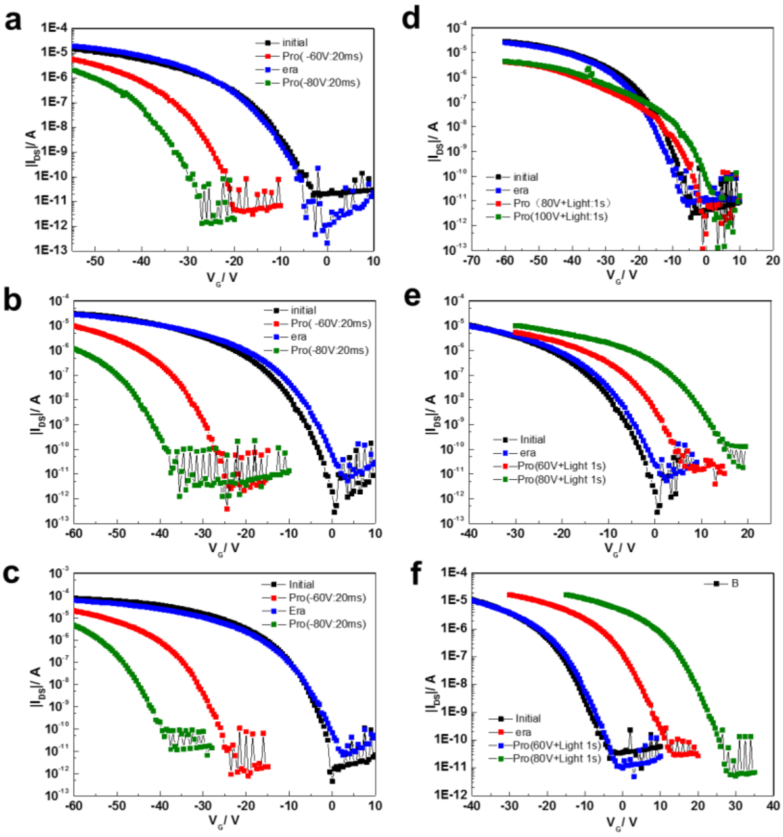

Figure. S18. The transfer curves of the (a) TPA(PDAF)$_1$@PS, (b) TPA(PDAF)$_2$@PS, (c) TPA(PDAF)$_3$@PS based devices for the programming processes under negative gate voltages ranging from -60 to -80 V. The drain current was measured at V_D = -30 V and the programming time was 20 ms. And (d) TPA(PDAF)$_1$@PS, (e) TPA(PDAF)$_2$@PS, (f) TPA(PDAF)$_3$@PS based devices for the positive programming processes upon positive gate voltages ranging from 60 to 80 V, with assist of light for 1 s.

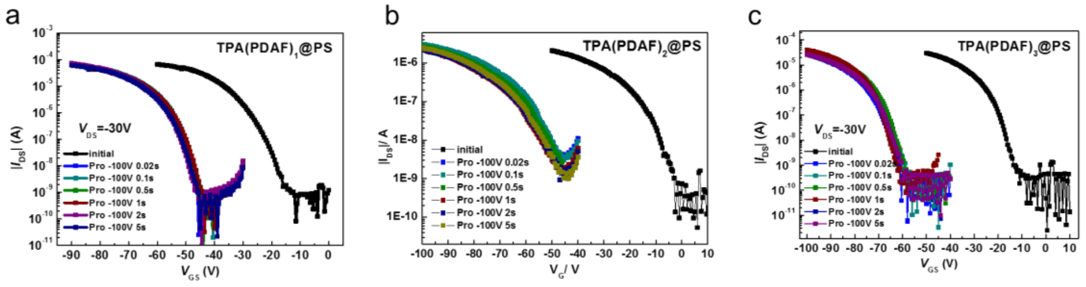

Figure. S19. The transfer characteristics of (a)TPA(PDAF)$_3$@PS, (b)TPA(PDAF)$_2$@PS and (c)TPA(PDAF)$_3$@PS based OFET memory. The programming time was changing from 20 ms to 5 s under -100 V gate voltage.

Table S1. Summary of calculated energies, ionization potential, and electron affinities for $TPA(PDAF)_n$(n=1,2,3)

	Neutral state			Cationic state			Anionic state		
	$TPA(PDAF)_1$	$TPA(PDAF)_2$	$TPA(PDAF)_3$	$TPA(PDAF)_1$	$TPA(PDAF)_2$	$TPA(PDAF)_3$	$TPA(PDAF)_1$	$TPA(PDAF)_2$	$TPA(PDAF)_3$
**Energy**	E_0 = -1513.03327912 a.u.	E_0 = -2276.36912235 a.u.	E_0 = -3039.70489571 a.u.	E_+ = -1512.80280738 a.u.	E_+ = -2276.14051614 a.u.	E_+ = -3039.47772805 a.u.	E_- = -1513.03718143 a.u.	E_- = -2276.38816714 a.u.	E_- = -3039.72996025 a.u.
**Potential Energy**	0	0	0	IP= 6.27 eV	IP= 6.22 eV	IP= 6.18 eV	EA= -0.11 eV	EA= -0.52 eV	EA= -0.68 eV

Table S2. Transistor and memory characteristics of various wt% $TPA(PDAF)_3$ mixed in PS.

Charge Trapping Elements	μ ($cm^2 V^{-1}s^{-1}$)	V_{th}(V)	I_{ON} / I_{OFF}	Negative Window (V)	Positive Window(V)
PS	0.76 ± 0.2	-11.99 ± 0.3	$7.1×10^5$	0	18.65
5%$TPA(PDAF)_3$@PS	0.56 ± 0.2	-16.19 ± 0.8	$2.12×10^6$	14.38	21.68
10%$TPA(PDAF)_3$@PS	0.55 ± 0.1	-11.04 ± 1.2	$2.85×10^7$	38.30	27.0
20%$TPA(PDAF)_3$@PS	0.37 ± 0.5	-12.96 ± 0.8	$1.07×10^5$	38.69	24.10
30%$TPA(PDAF)_3$@PS	0.25 ± 0.3	-10.35 ± 1.2	$4.12×10^5$	25.43	27.4
40%$TPA(PDAF)_3$@PS	0.09 ± 0.03	-5.27 ± 1.3	$7.37×10^4$	29.70	22.8

Programming conditions: V_G= -80 V for 20 ms. d) V_G= 80 V assist of light for 1 s.

^{1}H and ^{13}C-NMR spectra

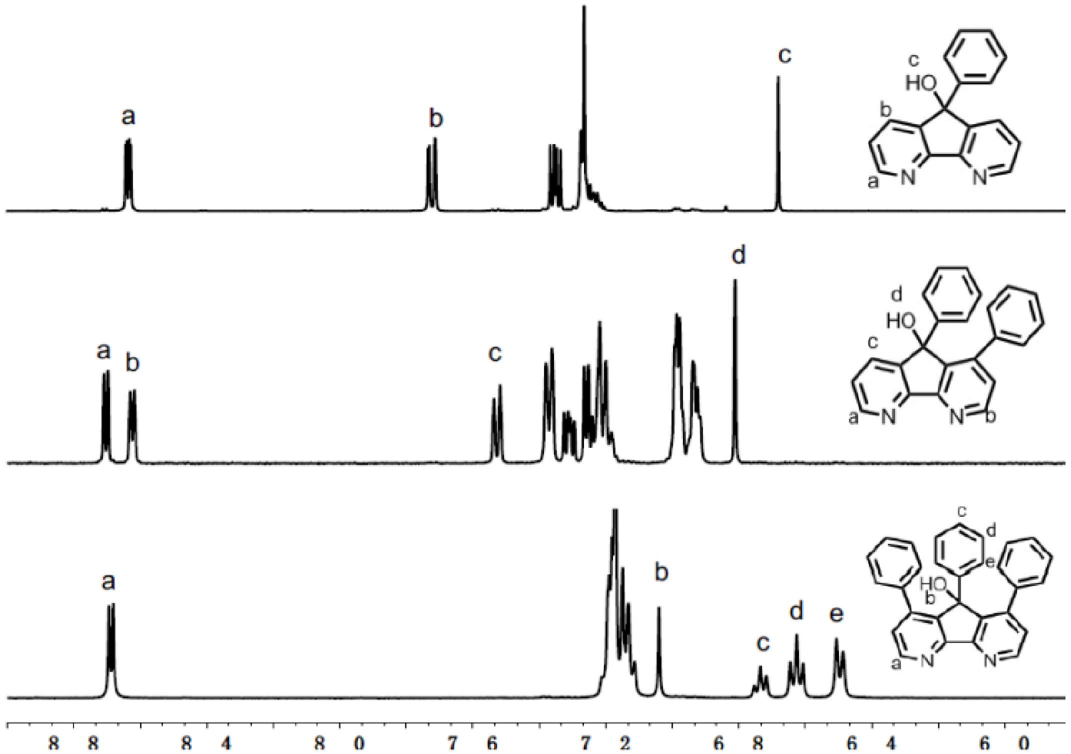

Figure. S20. Aromatic region of ^{1}H NMR spectra for nPDAFOH (n=1,2,3).

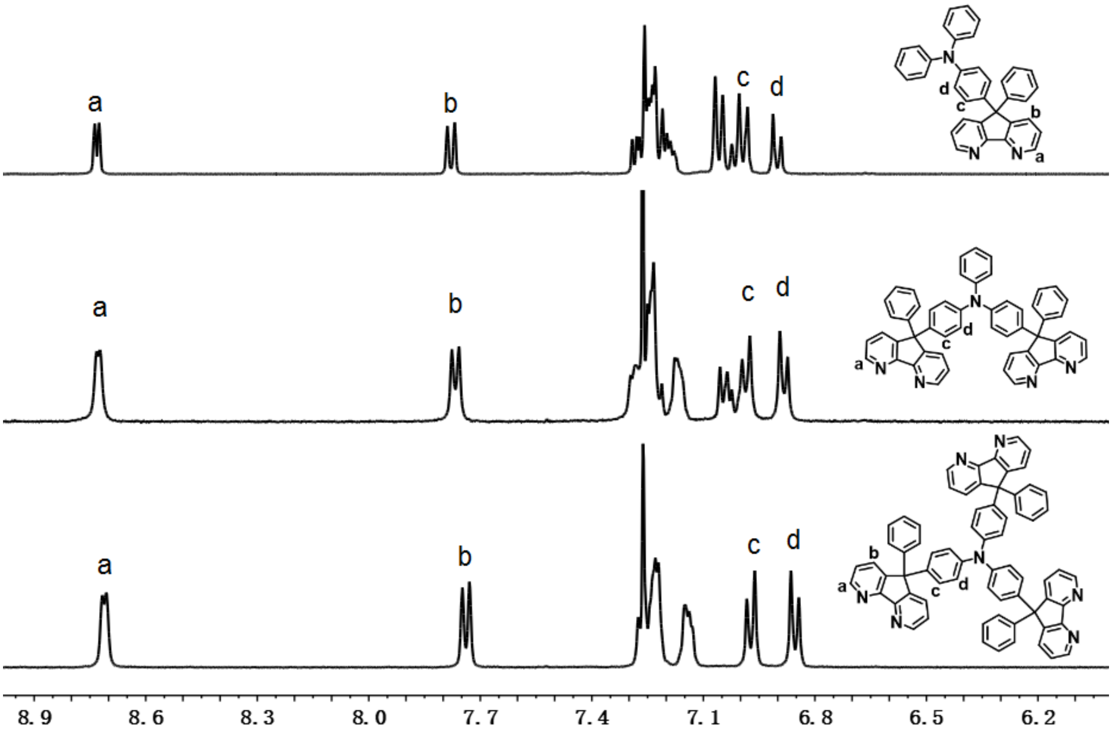

Figure. S21. Aromatic region of ^{1}H NMR spectra for TPA(PDAF)$_n$ (n=1,2,3).

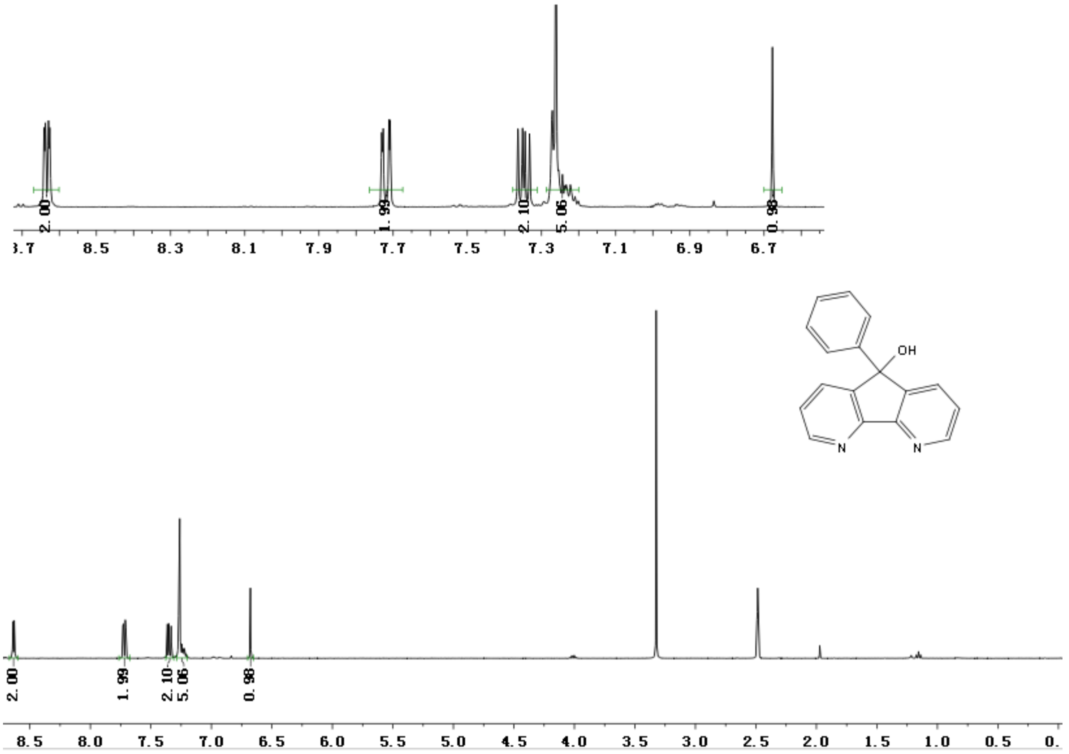

Figure. S22. ^{1}H NMR of **PDAFOH** in d_6-DMSO.

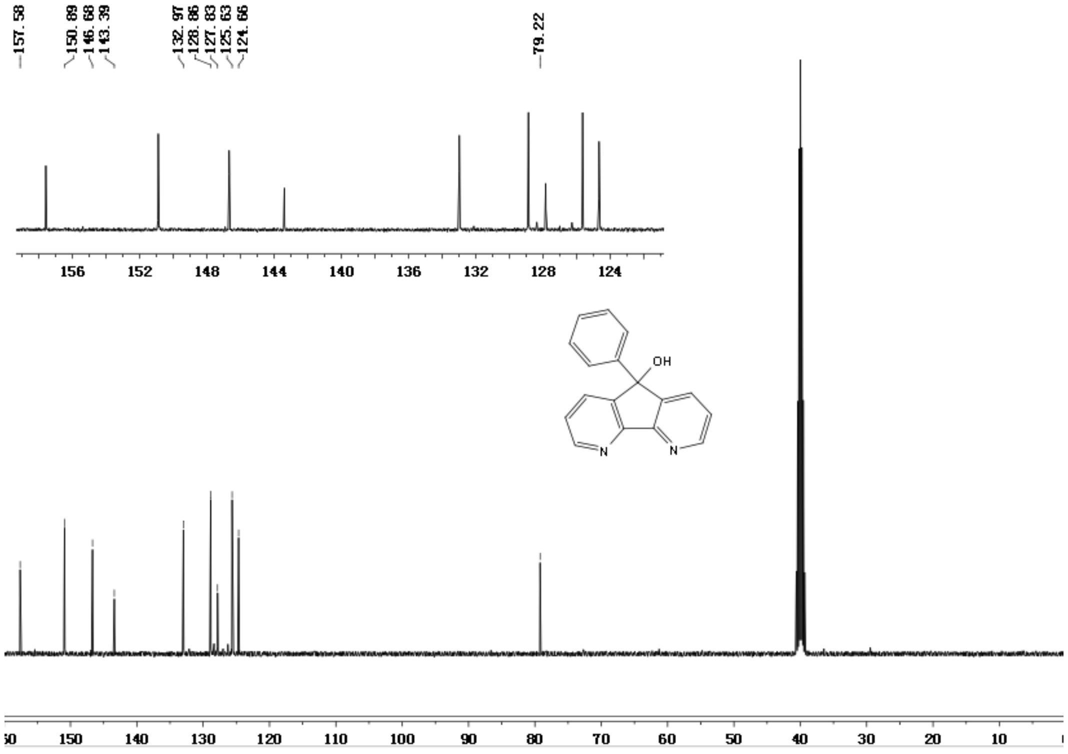

Figure. S23. ^{13}C NMR of **PDAFOH** in d_6-DMSO.

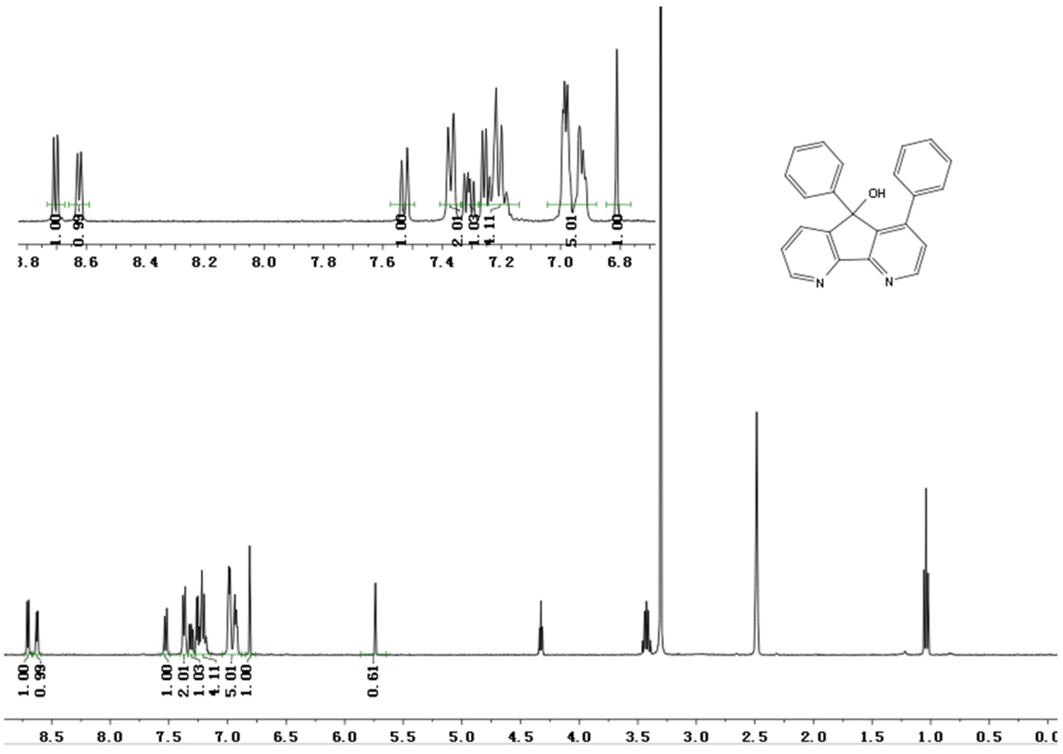

Figure. S24. ^{1}H NMR of **DPDAFOH** in d_6-DMSO.

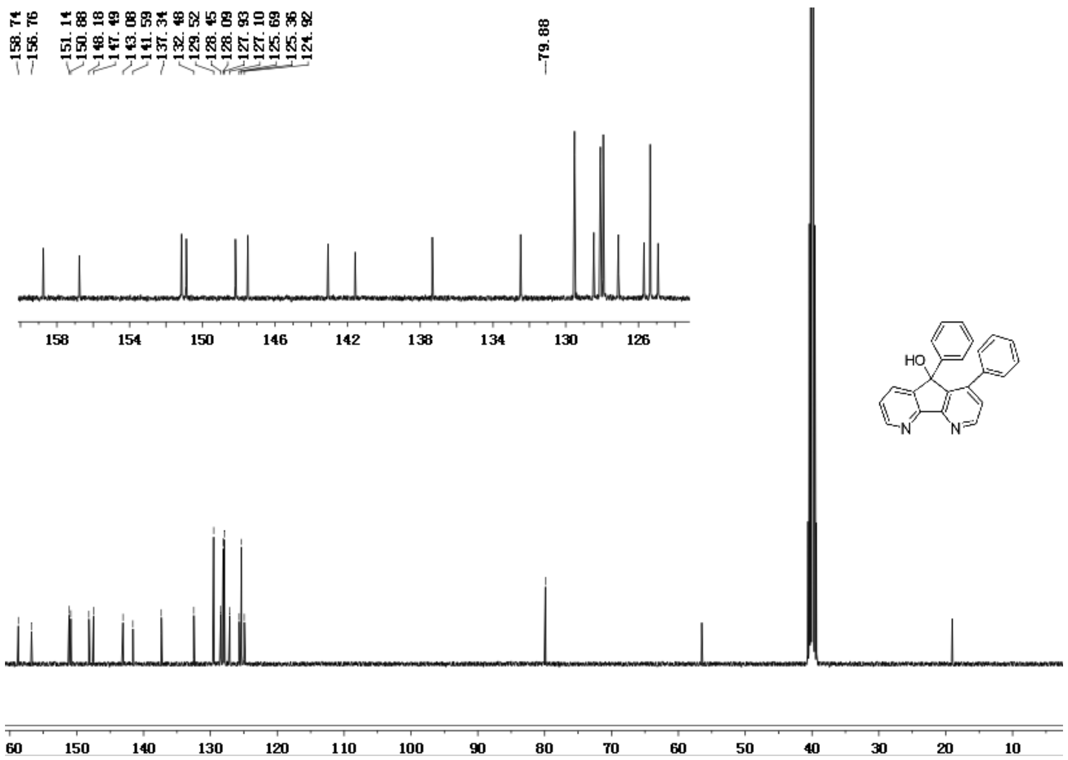

Figure. S25. ^{13}C NMR of **DPDAFOH** in d_6-DMSO.

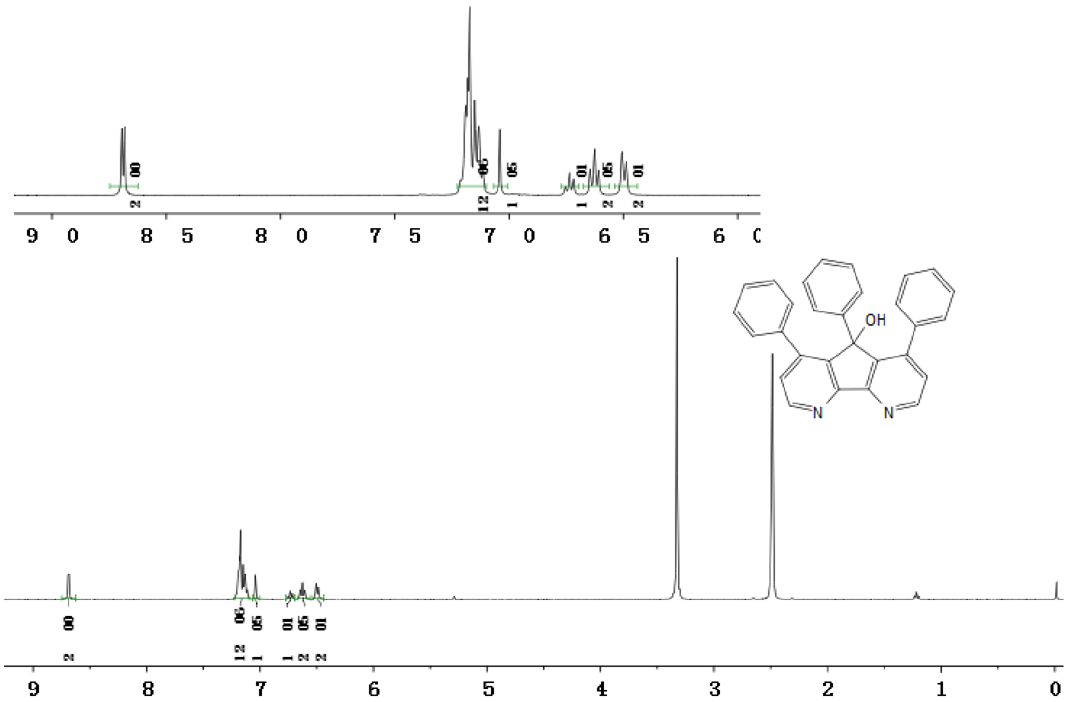

Figure. S26. ^{1}H NMR of **TPDAFOH** in d_6-DMSO.

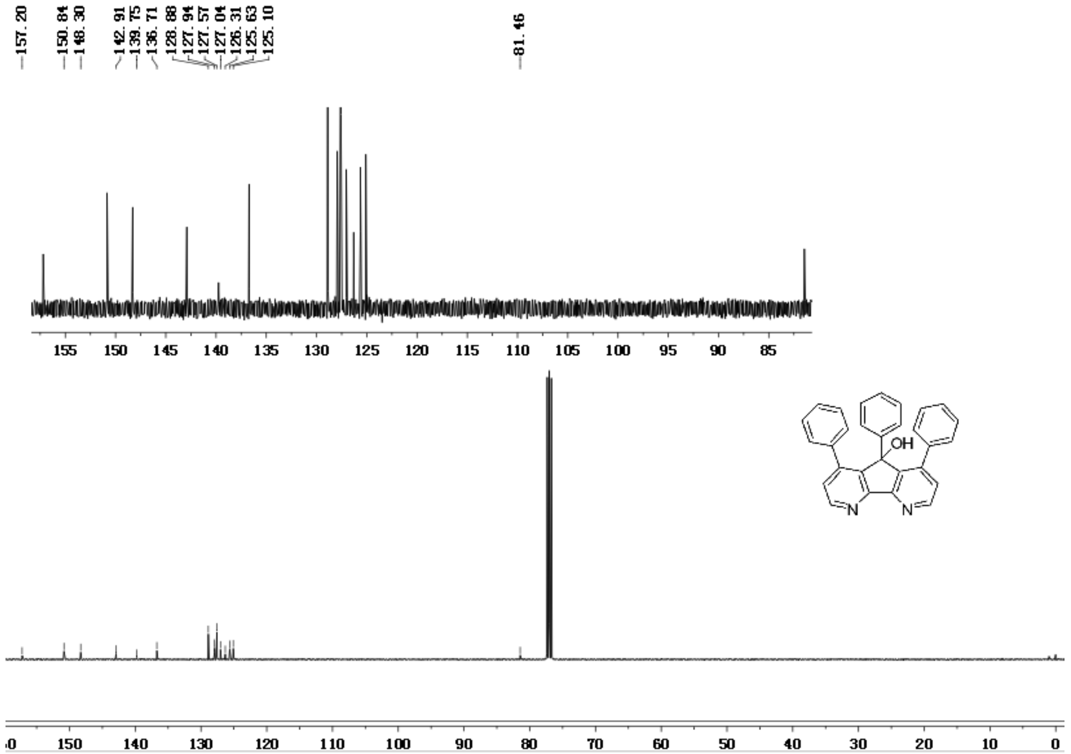

Figure. S27. ^{13}C NMR of **TPDAFOH** in $CDCl_3$.

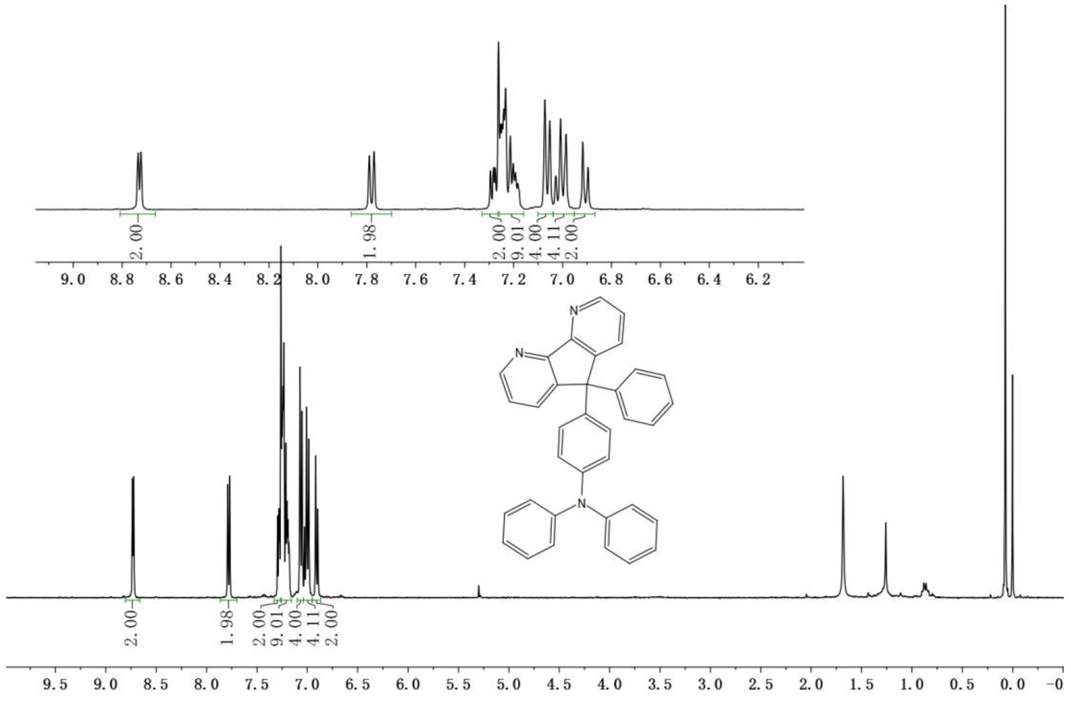

Fig. S28. ^{1}H NMR of **TPA(PDAF)$_1$** in $CDCl_3$.

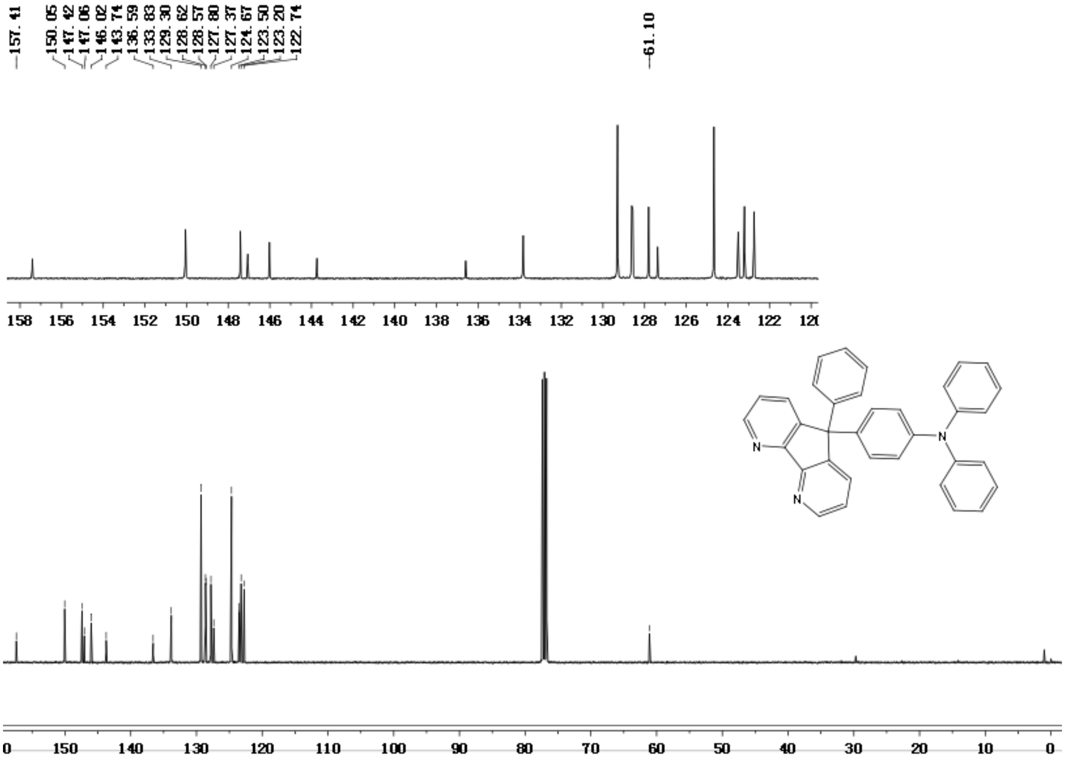

Fig. S29. ^{13}C NMR of **TPA(PDAF)$_1$** in $CDCl_3$.

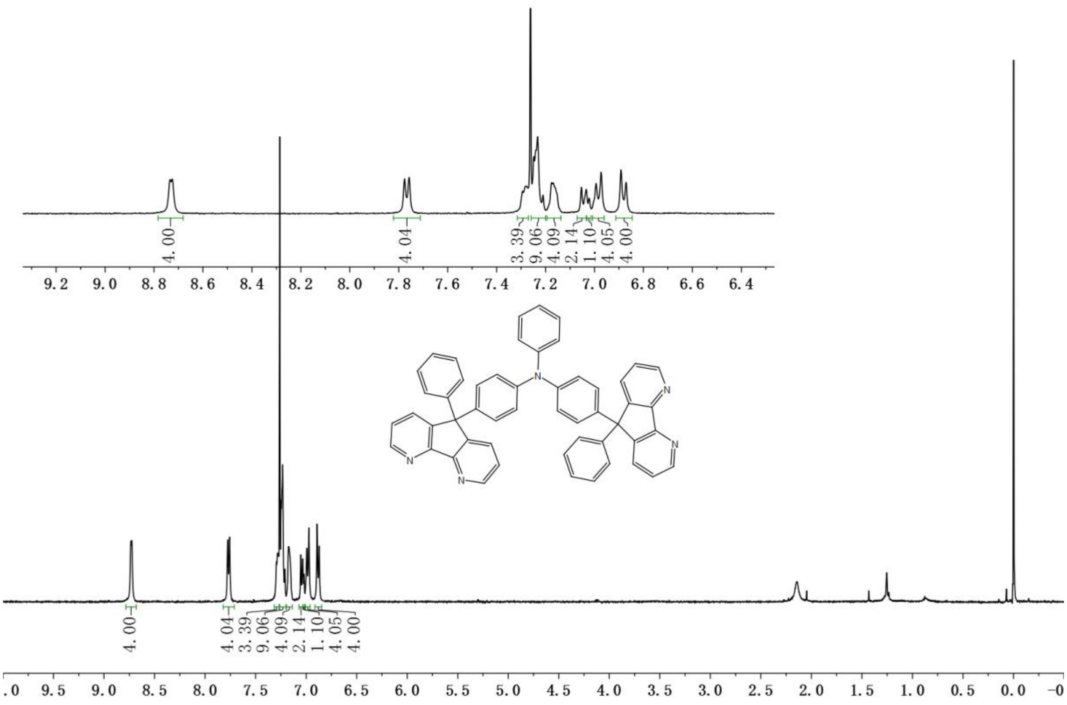

Fig. S30. ^1H NMR of **TPA(PDAF)$_2$** in $CDCl_3$.

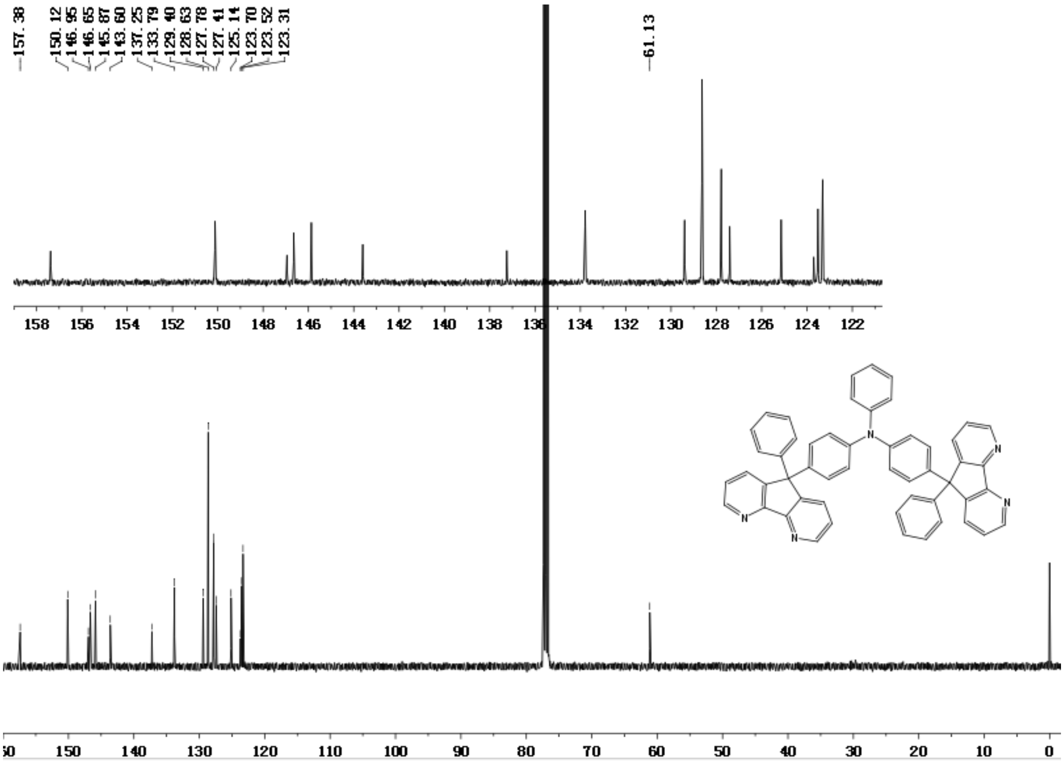

Fig. S31. ^{13}C NMR of **TPA(PDAF)$_2$** in $CDCl_3$.

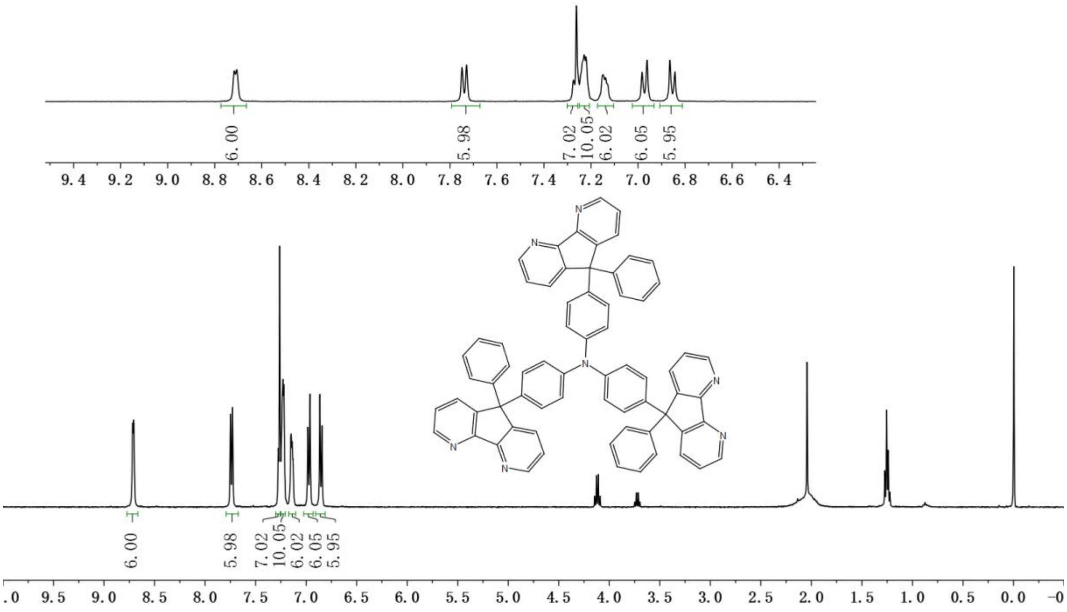

Fig. S32. ^{1}H NMR of **TPA(PDAF)$_3$** in $CDCl_3$.

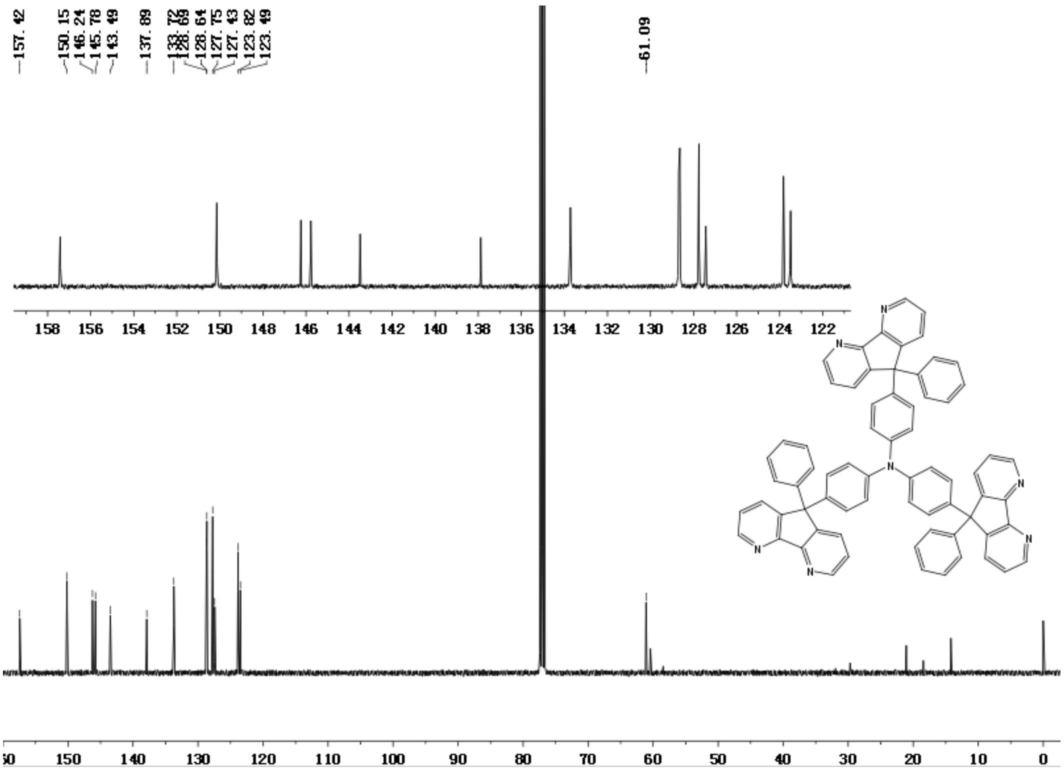

Fig. S33. ^{13}C NMR of **TPA(PDAF)$_3$** in $CDCl_3$.
